# Three-Component Reaction of 3-Arylidene-3*H*-Indolium Salts, Isocyanides, and Alcohols

**DOI:** 10.3389/fchem.2019.00345

**Published:** 2019-05-16

**Authors:** Nikita E. Golantsov, Hung M. Nguyen, Alexandra S. Golubenkova, Alexey V. Varlamov, Erik V. Van der Eycken, Leonid G. Voskressensky

**Affiliations:** ^1^Department of Organic Chemistry, Faculty of Science, Peoples' Friendship University of Russia (RUDN University), Moscow, Russia; ^2^Laboratory for Organic & Microwave-Assisted Chemistry (LOMAC), Department of Chemistry, University of Leuven (KU Leuven), Leuven, Belgium

**Keywords:** multicomponent reactions, isocyanides, indoleninium ions, aryl(indol-3-yl)methylium ions, imidates

## Abstract

A novel isocyanide-based multicomponent synthesis of alkyl aryl(indol-3-yl)acetimidates has been established. Starting from aryl(indol-3-yl)methylium tetrafluoroborates, aromatic isocyanides and alcohols, the imidates were obtained in moderate to very good yields. Consecutive four-component synthesis of the above mentioned imidates from N-alkylindoles, aromatic aldehydes, aromatic isocyanides and alcohols was also proposed. In addition, it was shown that in the presence of water, aryl(indol-3-yl)methylium tetrafluoroborates reacted with isocyanides to furnish aryl(indol-3-yl)acetamides.

## Introduction

Multicomponent reactions (MCRs) serve as a powerful and widely used instrument in organic synthesis (Shiri, 2012; Müller, 2014; Zarganes-Tzitzikas et al., 2015; Zhu et al., 2015; Levi and Müller, 2016). A special place among them is occupied by transformations with the participation of isocyanides, unique reagents where nucleophiles and electrophiles attack the same atom (for a book on isocyanides, see Nenajdenko, 2012; for selected reviews on isocyanide-based MCR, see Dömling and Ugi, 2000; Dömling, 2006; Sadjadi et al., 2016). Common reaction partners in MCR with isocyanides are iminium salts, carbonyl compounds (or oxocarbenium ions), and electron-deficient alkynes (Ugi, 1962; Banfi and Riva, 2005; De Moliner et al., 2011). Several examples of interaction between isocyanides and activated alkenes were reported (Saegusa et al., 1972; Person et al., 1980; Shaabani et al., 2002; Maltsev et al., 2006; Mironov et al., 2006; Jing et al., 2010; Soleimani and Zainali, 2011; Soleimani et al., 2011, 2013). Reactions of isocyanides with α,β-unsaturated imines or the corresponding iminium salts with the possibility of 1,4-addition are still rare (Marchand et al., 1999; Fontaine et al., 2009; Shimizu et al., 2009). Particularly, such imine and iminium salts were used in a formal [4+1] cycloaddition reaction with the formation of a pyrrole ring (Marchand et al., 1999; Fontaine et al., 2009; Kaur et al., 2016). In our opinion, the interaction of isocyanides with α,β-unsaturated imines could well-evolve into a novel multicomponent reaction.

**Figure 1:**
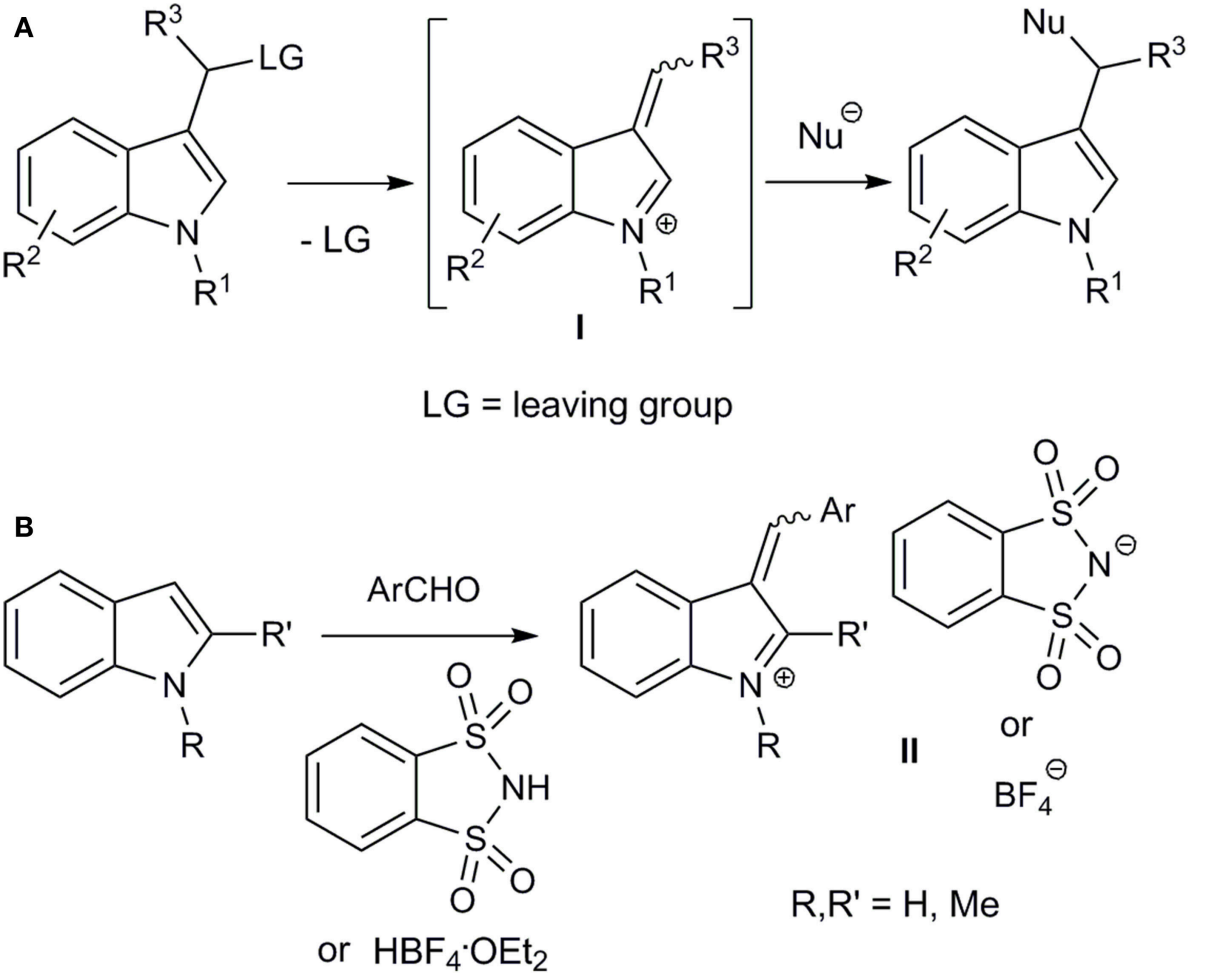
*In situ* generation of alkylideneindoleninium intermediates **(A)** and synthesis of stable aryl(indol-3-yl)methylium salts **(B)**.

Considering that the indole scaffold is privileged from medicinal chemistry point of view (Barreiro, 2016), we decided to use 3-arylidene-3*H*-indolium salts as simple vinylogues of iminium ions. Alkylideneindoleninium (3-alkylidene-3*H*-indolium) ions (**I**) can be formed as intermediates by cleaving the leaving group from the α-position (“benzylic”) of the substituent at the indole 3-position (Figure 1A) (For reviews, concerning generation and reactivity of alkylideneindolenine intermediates, see Enders et al., 2008; Shaikh et al., 2008; Palmieri et al., 2010; Wang et al., 2014; Zhuo et al., 2014; Jin et al., 2015; Palmieri and Petrini, 2016; Deb et al., 2017; Mei and Shi, 2017). Such intermediates are usually unstable and can react *in situ* with nucleophiles. This approach has been widely used in the syntheses of various indole derivatives (Enders et al., 2008; Shaikh et al., 2008; Palmieri et al., 2010; Wang et al., 2014; Zhuo et al., 2014; Jin et al., 2015; Palmieri and Petrini, 2016; Deb et al., 2017; Mei and Shi, 2017).

Moreover, relatively stable 3-arylidene-3*H*-indolium (aryl(indol-3-yl)methylium) ions **II** were obtained by acid-catalyzed coupling of indoles and aryl aldehydes, and were isolated and characterized as *o*-benzenedisulfonimide salts (Figure 1B) (Barbero et al., 2012). The corresponding diarylmethanes were obtained by reduction of these salts (Barbero et al., 2012), and an organocatalytic addition of aliphatic aldehydes to such compounds was developed (Armenise et al., 2015). Recently, an efficient synthesis for a bench-stable aryl(indol-3-yl)methyl tetrafluoroborate has been proposed (Figure 1B) (Barbero et al., 2015; Follet et al., 2015). Lewis acidity of such obtained aryl(indol-3-yl)methylium ions and kinetics of their interaction with different nucleophiles, including allylsilanes, enol silyl ethers, triarylphosphines, pyridines, secondary amines, have been studied (Follet et al., 2015, 2016). It should also be mentioned that the vinylogous iminium character of salts **II** was confirmed by single-crystal X-ray diffraction analysis (Barbero et al., 2015; Follet et al., 2015).

To the best of our knowledge, reactions of alkylideneindolenines or the corresponding salts with isocyanides have not been published yet. Herein, we report the three-component reaction of 3-arylidenindolium salts with isocyanides and alcohols to form alkyl aryl(indol-3-yl)acetimidates.

## Results and Discussion

**Figure 2:**
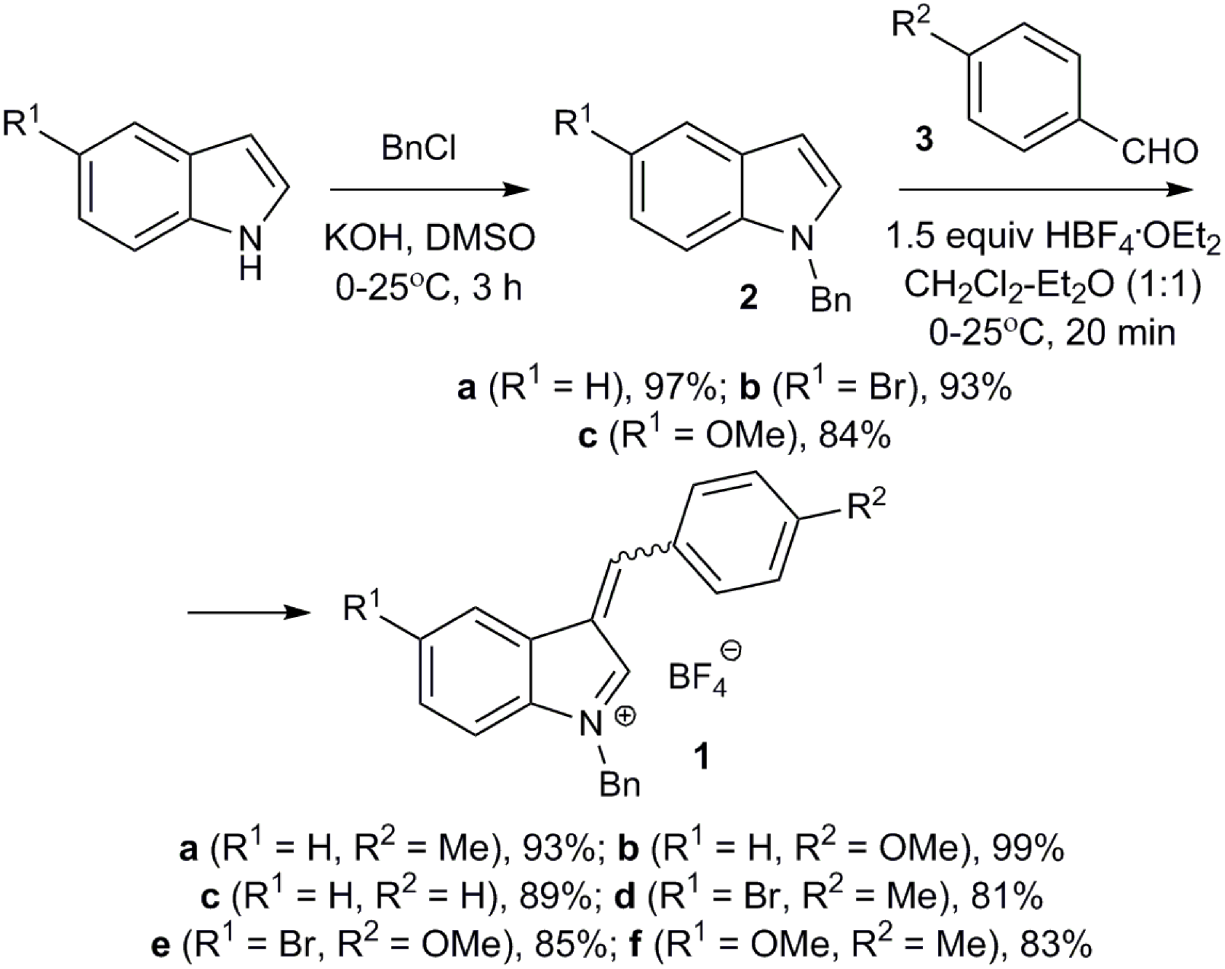
Synthesis of 3-arylidene-*3H*-indolium salts **1**.

Starting salts **1a-f** were obtained by alkylation of the corresponding indoles followed by reaction of *N*-benzylindoles **2a-c** with aromatic aldehydes **3a-c** under conditions similar to a previously published procedure (Figure 2) (Follet et al., 2015). Tetrafluoroborates **1a-f** containing a benzyl group in the indole 1-position remained unchanged after being stored at room temperature for several weeks and turned out to be more stable than their methyl analogs, whose lability was observed previously (Follet et al., 2015). In solution salts **1a-f** exist as a mixture of *E*- and *Z*-isomers (for copies of NMR spectra of obtained compounds, see Supplementary Material), what was also noted for 2-unsubstituted derivatives earlier (Follet et al., 2015).

**Figure 3:**
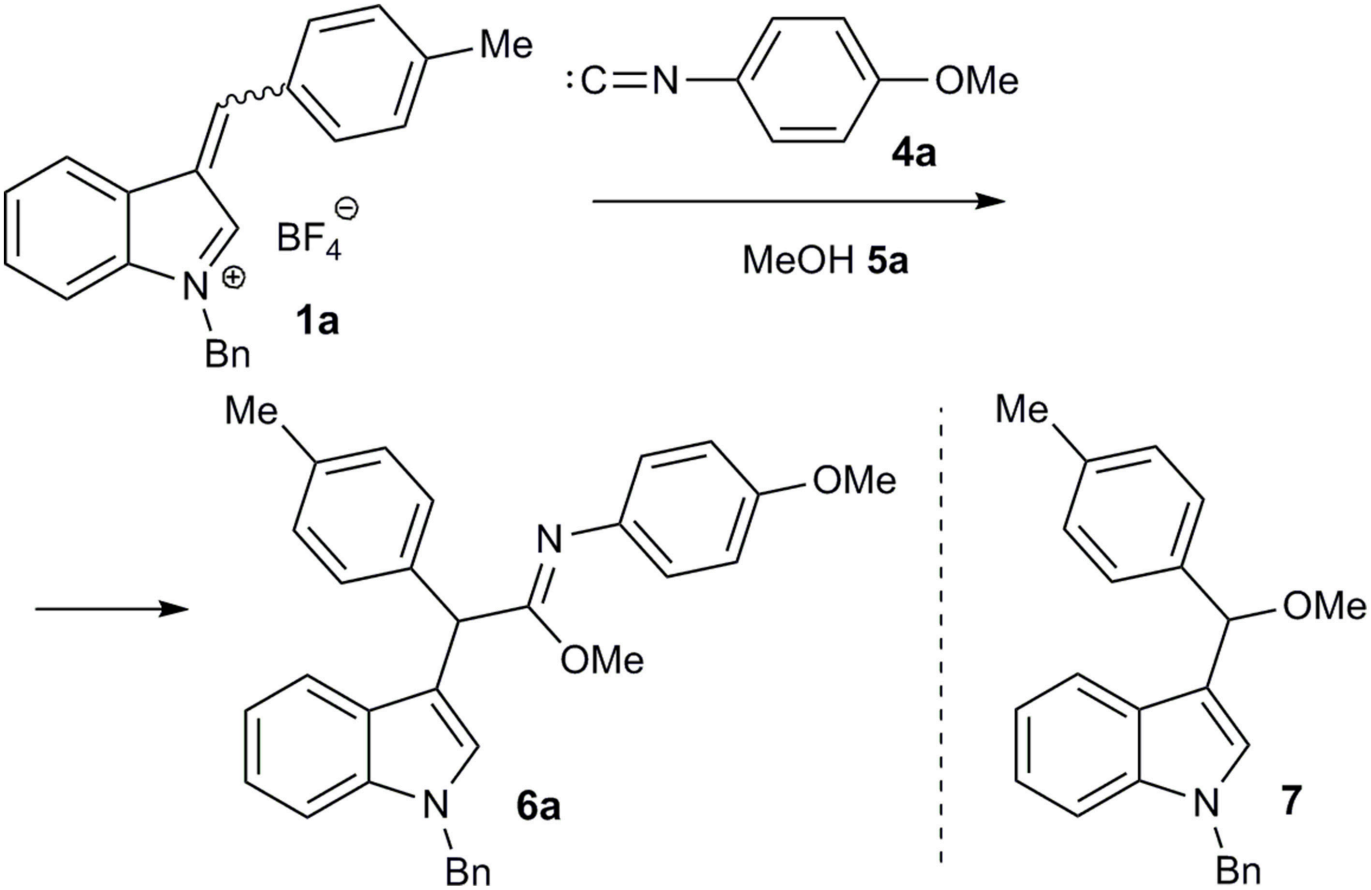
Reaction optimisation for imidate **6a** synthesis.

Next, the interaction of salt **1a** with *p*-methoxyphenyl isocyanide (**4a**) in methanol (**5a**) was performed at room temperature. After treating the reaction mixture with sodium bicarbonate solution, the imidate **6a** was isolated in moderate yield as a result of the expected three component interaction (Figure 3; Table 1, entry 1). This was stable upon chromatography on silica gel and further storage for several weeks. Unfortunately, our attempt to facilitate the reaction by heating was unsuccessful, due to significant tar formation (Table 1, entry 2). The addition of potassium carbonate as a base increased the yield (Table 1, entries 4, 7, 8), but led to the formation of ether **7**, resulting from the two-component reaction with methanol. Sodium or cesium carbonate had nearly the same effect, while Et_3_N was less effective (Table 1, entries 3, 5, 6).

**Table 1:** Reaction optimisation for imidate **6a** synthesis.

**Entry**	**MeOH (equiv.)**	**Solvent**	**Base (equiv.)**	**Temperature (^**°**^C)**	**Time (h)**	**Yield (%)^^d^^**
1^a^	80	MeOH	-	20	24 h	30
2^a^	80	MeOH	-	65	3 h	25
3^a^	80	MeOH	Na_2_CO_3_ (1.0)	20	12 h	45 (traces)
4^a^	80	MeOH	K_2_CO_3_ (1.0)	20	12 h	47 (traces)
5^a^	80	MeOH	Cs_2_CO_3_ (1.0)	20	12 h	43 (traces)
6^a^	80	MeOH	Et_3_N (1.0)	20	12 h	36 (18)
7^a^	80	MeOH	K_2_CO_3_ (1.5)	20	12 h	51 (12)
8^a^	80	MeOH	K_2_CO_3_ (2.0)	20	12 h	48 (15)
9^b^	80	MeOH	K_2_CO_3_ (1.5)	20	12 h	28
10^a^	4	MeCN	K_2_CO_3_ (1.5)	20	12 h	48
11^a^	40	MeCN – MeOH (1:1)	K_2_CO_3_ (1.5)	20	6 h	63
**12**^**a**^	**40**	**MeCN – MeOH (1:1)**	**K**_**2**_**CO**_**3**_ **(1.5)**	**20**	**12 h**	**65**
13^c^	80	MeOH	K_2_CO_3_ (1.5)	20	12 h	(91)
14^c^	80	MeOH	-	20	24 h	(traces)

a *1.3 equiv. **4a***.

b *2.0 equiv. **4a***.

c *0 equiv. **4a***.

d *the yield of **7** is indicated in parentheses*.

The formation of methoxy derivative **7** was not observed when the reaction was carried out in acetonitrile with 4 equiv of methanol and 1.5 equiv. of K_2_CO_3_, (Table 1, entry 10). However, the highest yield of the target product **6a** was achieved by using a mixture of equal amounts of methanol and acetonitrile as solvent (Table 1, entry 12).

In these optimized conditions, the reaction was showing significant progress by TLC during the first 3 h. After 6 h, the yield did not change substantially (Table 1, entries 11 and 12). It is worth noting that increasing the amount of isocyanide resulted in a drop of the yield (Table 1, entry 9 vs. 7), whereas, performing the reaction without the addition of isocyanide in the presence of a base led to the formation of two-component reaction product **7**. Without the addition of base, this product was only formed in trace amounts (Table 1, entries 13, 14).

**Figure 4:**
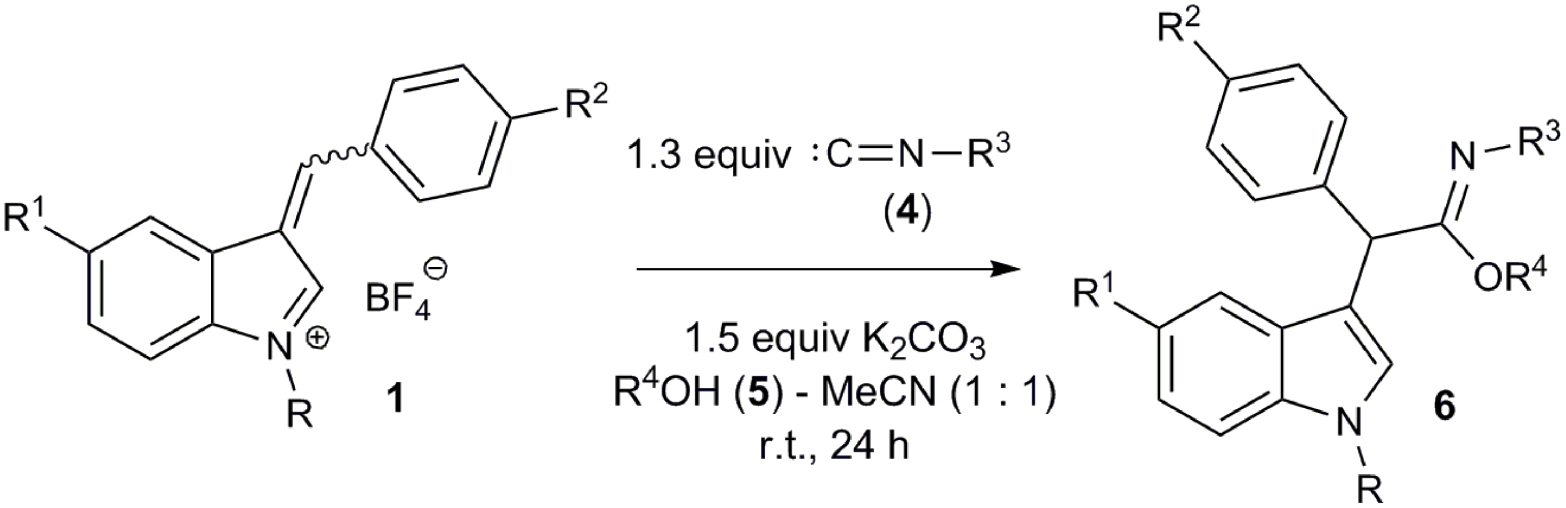
Synthesis of imidates **6**.

With the optimized conditions in hand, the interaction of salts **1a-f** with isocyanides **4a-d** and methanol was investigated (Figure 4; Table 2). Moreover, salts **1g-j** containing a methyl group at the indole nitrogen atom, which were obtained by a previously described procedure (Follet et al., 2015), were also involved. Reactions of aromatic isocyanides **4a-c** led to the corresponding imidates **6** in moderate to good yields. The best results were observed for salts derived from anisaldehyde (Table 2, entries 2, 5). Such results can be explained by stabilization of the 3-arylidene-3*H*-indolium salts **1b**,**h** with the donor substituent (MeO) and, consequently, by reducing the rate of the starting compound degradation under the reaction conditions, in comparison with more electrophilic salts **1a**,**c**,**g**,**i**. At the same time, the stronger electron-donating dimethylamino group notably reduced the electrophilicity of the substrate, which led to lowering the yield of the reaction product with isonitrile **4a** (Table 2, entry 7) (for electrophilicity parameters of salts **1j-g**, see Follet et al., 2015). Similarly, the presence of an electron-donating methoxy group at the indole 5-position slightly increased the yield of the corresponding imidate **6j** (Table 2, entry 1 vs. entry 10). The presence of a bromine atom in the same position decreased the yield (Table 2, entry 8, 9).

**Table 2:** Synthesis of imidates **6**.

**Entry**	**Salt**	**R**	**R^**1**^**	**R^**2**^**	**Isocyanide**	**R^**3**^**	**Alcohol**	**R^**4**^**	**Product**	**Yield, %**
1	**1a**	Bn	H	Me	**4a**	4-MeOC_6_H_4_	**5a**	Me	**6a**	65
2	**1b**	Bn	H	MeO	**4a**	4-MeOC_6_H_4_	**5a**	Me	**6b**	84
3	**1c**	Bn	H	H	**4a**	4-MeOC_6_H_4_	**5a**	Me	**6c**	56
4	**1g**	Me	H	Me	**4a**	4-MeOC_6_H_4_	**5a**	Me	**6d**	51
5	**1h**	Me	H	MeO	**4a**	4-MeOC_6_H_4_	**5a**	Me	**6e**	87
6	**1i**	Me	H	H	**4a**	4-MeOC_6_H_4_	**5a**	Me	**6f**	78
7	**1j**	Me	H	Me_2_N	**4a**	4-MeOC_6_H_4_	**5a**	Me	**6g**	51
8	**1d**	Bn	Br	Me	**4a**	4-MeOC_6_H_4_	**5a**	Me	**6h**	47
9	**1e**	Bn	Br	MeO	**4a**	4-MeOC_6_H_4_	**5a**	Me	**6i**	52
10	**1f**	Bn	MeO	Me	**4a**	4-MeOC_6_H_4_	**5a**	Me	**6j**	71
11	**1a**	Bn	H	Me	**4a**	4-MeOC_6_H_4_	**5b**	Et	**6k**	53
12	**1b**	Bn	H	MeO	**4a**	4-MeOC_6_H_4_	**5b**	Et	**6l**	47
13	**1b**	Bn	H	MeO	**4a**	4-MeOC_6_H_4_	**5c**	*i*-Pr	**6m**	56
14	**1a**	Bn	H	Me	**4b**	Ph	**5a**	Me	**6n**	51
15	**1b**	Bn	H	MeO	**4b**	Ph	**5a**	Me	**6o**	63
16	**1g**	Me	H	Me	**4b**	Ph	**5a**	Me	**6p**	54
17	**1b**	Bn	H	MeO	**4c**	4-ClC_6_H_4_	**5a**	Me	**6q**	43
18	**1f**	Bn	MeO	Me	**4c**	4-ClC_6_H_4_	**5a**	Me	**6r**	37
19	**1a**	Bn	H	Me	**4d**	Bn	**5a**	Me	**6s**	0^a^

a * The corresponding amide **8a** (see Table 4) was isolated in 17 % yield along with compound **7** in 25 % yield*.

Among aromatic isocyanides, the best results were also achieved for isocyanide **4a** containing an electron-donating methoxy group. Furthermore, the alcohol component of this MCR could be varied and the reaction was carried out in a mixture of acetonitrile with alcohols **5b,c** (Table 2, entries 11-13).

It should be mentioned that, in case of the non-conjugated benzyl isocyanide **4d**, it was not possible to isolate the corresponding imidate **6s**. Within 12 h, the reaction did not show significant progress according to TLC. After work-up and purification by chromatography on silica gel, amide **8a** was isolated in a low yield along with compound **7** (Table 2, entry 19). Apparently, the amide **8a** was formed by hydrolysis of the corresponding imidate **6s**.

**Figure 5:**
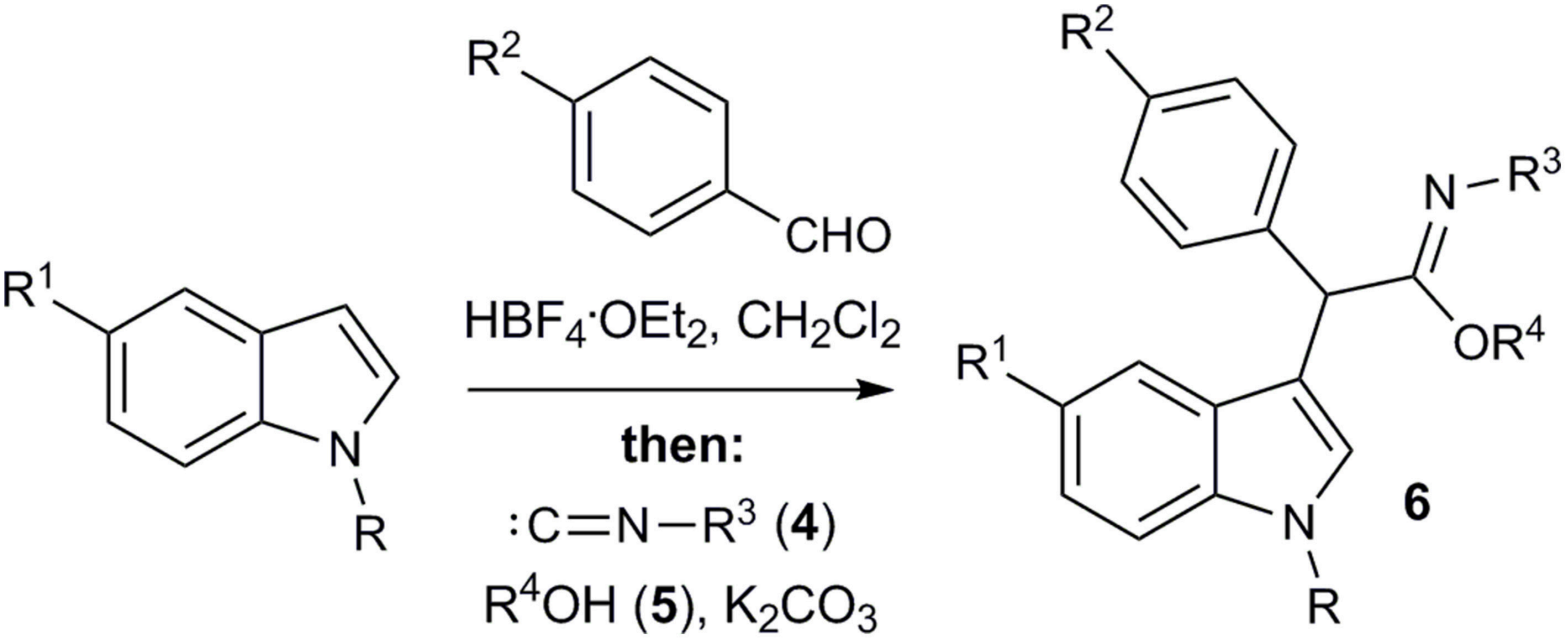
Consecutive four-component synthesis of imidates **6**.

Next, the possibility of imidate synthesis from the indole and the aldehyde by a sequential one-pot four-component process, without isolation of the corresponding 3-arylidene-3*H*-indolium salt, was investigated (Figure 5; Table 3). After acid-catalyzed condensation of the aldehyde with 1-alkylindole, an excess of K_2_CO_3_ and a solution of isocyanide in the appropriate alcohol were added to the reaction mixture. To our great satisfaction, target imidate **6** was obtained in moderate to good yields.

**Table 3:** Consecutive four-component synthesis of imidates **6**^a^.

**Entry**	**Product**	**R**	**R^**1**^**	**R^**2**^**	**R^**3**^**	**R^**4**^**	**Yield, %**
1	**6b**	Bn	H	MeO	4-MeOC_6_H_4_	Me	74
2	**6e**	Me	H	MeO	4-MeOC_6_H_4_	Me	77
3	**6f**	Me	H	H	4-MeOC_6_H_4_	Me	68
4	**6g**	Me	H	Me_2_N	4-MeOC_6_H_4_	Me	24
5	**6i**	Bn	Br	MeO	4-MeOC_6_H_4_	Me	40
6	**6j**	Bn	MeO	Me	4-MeOC_6_H_4_	Me	56
7	**6l**	Bn	H	MeO	4-MeOC_6_H_4_	Et	37
8	**6m**	Bn	H	MeO	4-MeOC_6_H_4_	*i*-Pr	46
9	**6n**	Bn	H	Me	Ph	Me	41
10	**6q**	Bn	H	MeO	4-ClC_6_H_4_	Me	36

a * Reaction conditions: 1.0 equiv. of aldehyde, 1.5 equiv. HBF_4_OEt_2_, CH_2_Cl_2_, 0 – 20°C then, 1.3 equiv. 4, 2.0 equiv. K_2_CO_3_, R^4^OH (1:1 to CH_2_Cl_2_), rt, 12 h*.

**Figure 6:**
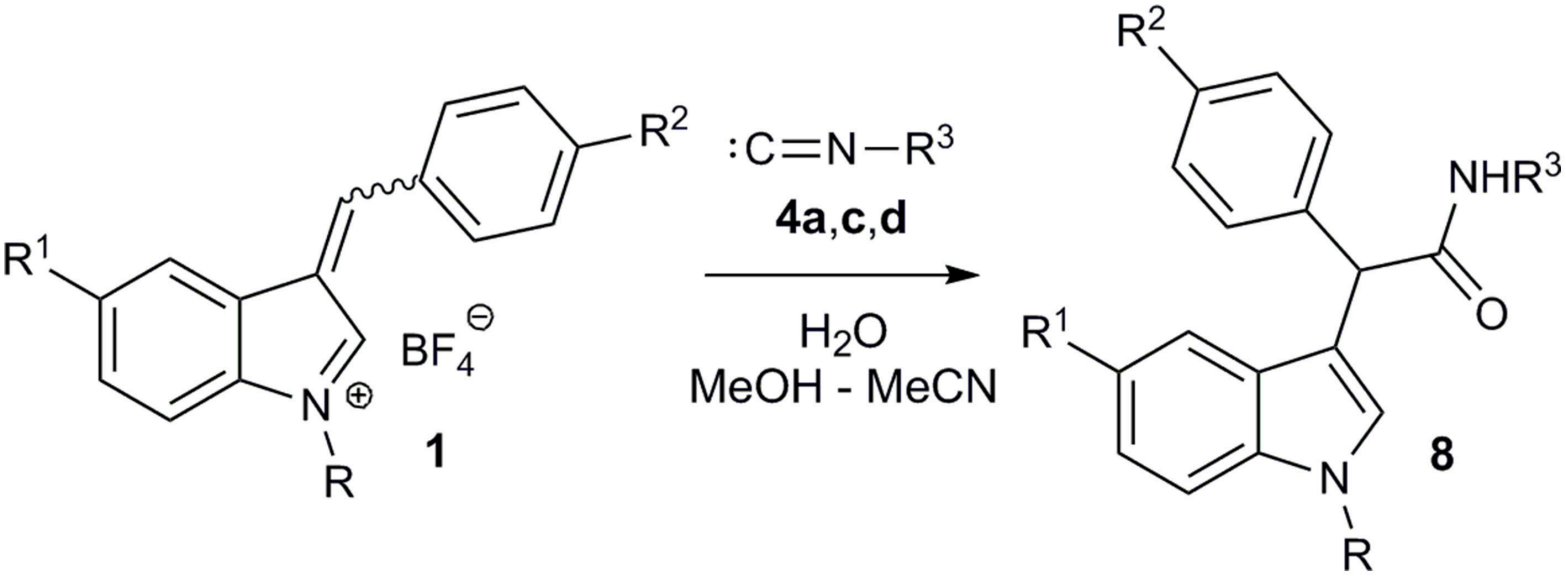
Synthesis of amides **8**.

Then we decided to extend our three-component imidate synthesis with the aim to obtain amides **8** (Figure 6; Table 4). As it turned out, addition of 10 volume % of water to the reaction mixture delivered the desired amides. Moreover, under these conditions, the reaction with benzyl isocyanide **4d** also proceeded very quickly. A white precipitate of the corresponding amide **8a** appeared almost immediately after isocyanide **4d** addition. The interaction with aromatic isocyanides **4a,c** was slightly slower but also efficient (Table 4, entries 7 and 8). Thus, 8 examples of amides **8** were obtained in a moderate to good yields employing this procedure (Table 4).

**Table 4:** Synthesis of amides **8**^a^.

**Entry**	**Product**	**R**	**R^**1**^**	**R^**2**^**	**R^**3**^**	**Yield (%)**
1	**8a**	Bn	H	Me	Bn	59
2	**8b**	Bn	H	MeO	Bn	63
3	**8c**	Me	H	MeO	Bn	53
4	**8d**	Me	H	H	Bn	50
5	**8e**	Bn	Br	Me	Bn	51
6	**8f**	Bn	MeO	Me	Bn	57
7	**8g**	Bn	H	MeO	4-MeOC_6_H_4_	44
8	**8h**	Bn	H	MeO	4-ClC_6_H_4_	54

a * Reaction conditions: 1.3 equiv. **4**, 1.5 equiv. K_2_CO_3_, MeOH – MeCN – H_2_O (1:1:0.2), r.t., 12 h*.

**Figure 7:**
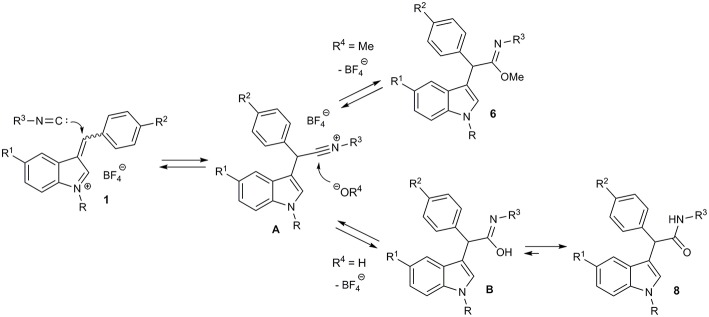
Proposed mechanism of imidates **6** and amides **8** formation.

Previously, low yields were observed in formation of imidates with the conjugate addition of alkyl isocyanides to methyl acrylate and acrylonitrile in methanol (Saegusa et al., 1971). Amides were reported to be formed in reaction of dicyanoethylenes with isocyanides (Soleimani et al., 2011). Based on these facts and on the reported reactivity of 3-arylidene-3*H*-indolium salts (Follet et al., 2015, 2016), as well as on our own experiments, we suggest the following pathway for the multicomponent transformation of 3-arylidene-3*H*-indolium salts (Figure 7). The conjugate nucleophilic addition of isocyanide to the vinylogous iminium ion **1** leads to nitrilium salt **A**, which is further attacked by the nucleophile. This could be an alcoholate ion (or the corresponding alcohol). As a result, the imidate **4** is formed. Therefore, the role of the base in this process is to generate a small concentration of alcoholate ion as a strong nucleophile and to bind the released HBF_4_.

The unsuccessful attempt of the three-component reaction with non-conjugated isocyanide **4d** can be explained by the reversibility of the process and the lower stability of the corresponding imidate. In the case that water is present, the hydroxide ion acts as nucleophile leading to the imidic acid **B**, which is in equilibrium with the corresponding amide **8**. This last step makes the entire sequence of reactions almost irreversible.

## Conclusions

We have elaborated a three-component reaction of a 3-arylidene-3*H*-indolium salt, an aromatic isocyanide and an alcohol, leading to a series of alkyl aryl(indol-3-yl)acetimidates with yields up to 87%. We have also established a consecutive four-component synthesis of the above mentioned imidates from a *N*-alkylindole, an aromatic aldehyde, an aromatic isocyanide and an alcohol. By using aqueous acetonitrile-methanol media we have expanded our method for the synthesis of aryl(indol-3-yl)acetamides. These reactions present a new practical synthetic approach to a series of compounds possessing a privileged indole scaffold and then also extend isocyanide-based MCRs by using vinylogous iminium ions.

## Materials and Methods

### General

Starting reagents were purchased from commercial sources and were used without any additional purification or were prepared according to literature procedures. ^1^H and ^13^C NMR spectra were acquired on a Jeol JNM-ECA 600 spectrometer (with operating frequencies of 600 and 150 MHz, respectively) at room temperature and referenced to the residual signals of the solvent. The solvents used for NMR were DMSO-*d*_6_ and CDCl_3_. Chemical shifts are reported in parts per million (δ/ppm). Coupling constants are reported in Hertz (J/Hz). The peak patterns are indicated as follows: s, singlet; d, doublet; t, triplet; q, quadruplet; m, multiplet; dd, doublet of doublets and br s, broad singlet. Infrared spectra were measured on an Infralum FT-801 FT/IR instrument. The wavelengths are reported in reciprocal centimeters (νmax /cm^−1^). Mass spectra were recorded with LCMS-8040 Triple quadrupole liquid chromatograph mass-spectrometer from Shimadzu (ESI) and Kratos MS-30 mass-spectrometer (EI, 70 eV). HRMS spectra were recorded on a Bruker MicrOTOF-Q II. Elemental analysis was performed on an Euro Vector EA-3000 elemental analyzer. The reaction progress was monitored by TLC and the spots were visualized under UV light (254 or 365 nm). Column chromatography was performed using silica gel (230–400 mesh). Melting points were determined on a SMP-10 apparatus and were uncorrected. Solvents were distilled and dried according to standard procedures.

### Synthesis of Tetrafluoroborates 1a-f

#### 1-Benzyl-3-(4-Methylbenzylidene)-3H-Indolium Tetrafluoroborate (1a); Typical Procedure

1*H*-Indole (5.00 g, 42.7 mmol) and benzyl chloride (7.30 ml, 63.4 mmol) were dissolved in 10 ml of dry DMSO and finely ground KOH (3.5 g, 62.5 mmol) was added at 0–5°C in one portion. The reaction mixture was stirred at r.t. for 3 h and then poured into 100 mL of cold water. The product was extracted with ether (2 × 50 mL). The combined organic layers were washed with water (50 mL), brine (30 mL) and dried over anhydrous Na_2_SO_4_, after which the solvent was removed in vacuo. The residue was purified by flash chromatography on short column of silica gel (EtOAc – hexane, 20:1) to afford 1-benzyl-3*H*-indole (**2a**) (8.6 g, 97%) as white crystals.

1-Benzyl-1*H*-indole (**2a**) (1.00 g, 4.82 mmol) and *p*-tolualdehyde (**3a**) (4.82 mmol) were dissolved in a mixture of dry CH_2_Cl_2_ (5 mL) and Et_2_O (5 mL). Then HBF_4_·OEt_2_ (1.00 ml, 7.35 mmol) was added dropwise at 0–5°C over a period of 2 min. The reaction mixture was allowed to warm to r.t. and stirred for 20 min. The resulting precipitate was filtered, washed thoroughly with Et_2_O (5 × 20 mL) to give 1-benzyl-3-(4-methylbenzylidene)-3*H*-indolium tetrafluoroborate.

Yield: 1.78 g (93%); bright orange solid; mp 201–203°C (dec.).

IR (film): 3438, 3127, 1736, 1621, 1587, 1527, 1447, 1369, 1299, 1257, 1186, 1100, 1073, 1048, 813, 761, 711 cm^−1^.

^1^H NMR (600 MHz, CDCl_3_+TFA; ~ 3:1 *Z*/*E* diastereomeric mixture): δ = 9.17 (s, 0.67 H), 8.91 (s, 0.33 H), 8.82 (s, 0.67 H), 8.71 (s, 0.33 H), 8.32 (d, *J* = 8.1 Hz, 0.33 H), 8.01 (d, *J* = 8.0 Hz, 0.67 H), 7.97 (d, *J* = 8.1 Hz, 0.67 H), 7.91–7.85 (m, 1.33 H), 7.65–7.52 (m, 3 H), 7.50–7.36 (m, 7 H), 5.70 (s, 1.33 H), 5.60 (s, 0.67 H), 2.54 (s, 1 H), 2.49 (s, 2 H).

^13^C NMR (150 MHz, CDCl_3_+TFA): δ = 165.3, 160.6, 159.7, 153.4, 150.6, 149.7, 141.6, 139.9, 134.8, 134.2, 131.7, 131.4, 131.0, 130.95, 130.90, 130.6, 130.1, 129.97, 129.94, 129.86, 129.7, 129.64, 129.60, 129.2, 128.5, 128.45, 128.3, 127.09, 127.07, 125.2, 124.1, 121.2, 115.1, 115, 54.7, 54.2, 22.5, 22.3.

HRMS (TOF ES^+^): *m*/*z* [M – ${\text{BF}}_{4}^{-}$]^+^ calcd for C_23_H_20_N^+^: 310.1590; found: 310.1596.

#### 1-Benzyl-3-(4-Methoxylbenzylidene)-3H-Indolium Tetrafluoroborate (1b)

Red solid; yield 1.97 g (99% from 1.00 g 1-benzyl-1*H*-indole (**2a**)); mp 214-216°C (dec.).

IR (film): 3130, 2956, 2917, 2847, 1736, 1581, 1556, 1520, 1441, 1370, 1281, 1262, 1173, 1098, 1062, 835, 760, 710, 587 cm^−1^.

^1^H NMR (600 MHz, CDCl_3_+TFA; ~ 2.3:1 *Z*/*E* diastereomeric mixture): δ = 9.06 (s, 0.7 H), 8.75 (s, 0.7 H), 8.72 (s, 0.3 H), 8.62 (s, 0.3 H), 8.37 – 8.32 (m, 0.3 H), 8.16 (d, *J* = 9.0 Hz, 0.6 H), 8.04 (d, *J* = 8.6 Hz, 1.4 H), 7.99 (d, *J* = 8.1 Hz, 0.7 H), 7.66 – 7.52 (m, 3 H), 7.48 – 7.34 (m, 5 H), 7.22 – 7.15 (m, 2 H), 5.68 (s, 1.4 H), 5.58 (s, 0.6 H), 4.04 (s, 0.9 H), 4.01 (s, 2.1 H).

^13^C NMR (150 MHz, CDCl_3_+TFA): δ = 169.2, 168.3, 164.5, 159.0, 158.6, 150.9, 142.4, 139.5, 138.7, 138.5, 131.9, 131.3, 130.1, 130.0, 129.97, 129.95, 129.90, 129.13, 129.10, 129.0, 128.9, 128.5, 128.1, 127.5, 127.0, 126.9, 125.3, 124.4, 123.7, 120.7, 117.2, 116.3, 114.8, 114.5, 56.6, 56.5, 54.4, 53.9.

HRMS (TOF ES^+^): *m*/*z* [M – ${\text{BF}}_{4}^{-}$]^+^ calcd for C_23_H_20_NO^+^: 326.1539; found: 326.1550.

#### 1-Benzyl-3-Benzylidene-3H-indolium Tetrafluoroborate (1c)

Bright orange solid; yield 1.64 g (89% from 1.00 g 1-benzyl-1*H*-indole (**2a**)); mp 163-165°C (dec.).

IR (film): 3416, 3115, 1619, 1605, 1587, 1568, 1531, 1447, 1370, 1296, 1253, 1192, 1099, 1050, 826, 759, 710, 678, 599, 570 cm^−1^.

^1^H NMR (600 MHz, CDCl_3_+TFA; ~ 1.7:1 *Z*/*E* diastereomeric mixture): δ = 9.15 (s, 0.63 H), 8.93 (s, 0.37 H), 8.90 (s, 0.63 H), 8.74 (s, 0.37 H), 8.29 (d, *J* = 7.7 Hz, 0.37 H), 8.04 (d, *J* = 7.7 Hz, 0.63 H), 8.02 (d, *J* = 8.0 Hz, 0.76 H), 7.92 (d, *J* = 7.7 Hz, 1.26 H), 7.80 – 7.72 (m, 1 H), 7.69 – 7.56 (m, 5 H), 7.48 – 7.36 (m, 5 H), 5.72 (s, 1.26 H), 5.62 (s, 0.76 H).

^13^C NMR (150 MHz, CDCl_3_+TFA): δ = 165.0, 161.4, 159.6, 154.5, 142.9, 140.4, 136.9, 136.4, 134.1, 133.72, 133.70, 133.4, 133.3, 131.03, 131.0, 130.7, 130.6, 130.5, 130.4, 130.3, 130.2, 130.1, 130.03, 130.0, 129.97, 128.7, 128.6, 125.4, 128.2, 125.1, 124.3, 121.6, 115.3, 115.2, 55.0, 54.5.

HRMS (TOF ES^+^): *m*/*z* [M – ${\text{BF}}_{4}^{-}$]^+^ calcd for C_22_H_18_N^+^: 296.1434; found: 296.1441.

#### 1-Benzyl-5-Bromo-3-(4-Methylbenzylidene)-3H-Indolium Tetrafluoroborate (1d)

Bright orange solid; yield 1.35 g (81% from 1.00 g 1-benzyl-5-bromo-1*H*-indole (**2b**)); mp 220-222°C (dec.).

IR (film): 3126, 1736, 1620, 1587, 1526, 1448, 1424, 1372, 1319, 1259, 1188, 1104, 1051, 802, 772, 743, 714, 653, 629 cm^−1^.

^1^H NMR (600 MHz, CDCl_3_+TFA; ~ 2.3:1 *Z*/*E* diastereomeric mixture): δ = 9.13 (s, 0.70 H), 8.85 (s, 0.70 H), 8.83 (s, 0.30 H), 8.76 (s, 0.30 H), 8.49 (d, *J* = 2.0 Hz, 0.30 H), 8.18 (d, *J* = 2.0 Hz, 0.70 H), 7.99 (d, *J* = 8.1 Hz, 0.60 H), 7.90 (d, *J* = 8.1 Hz, 1.40 H), 7.75 (dd, *J* = 8.7 Hz, 1.7 Hz, 0.30 H), 7.71 (dd, *J* = 8.7 Hz, 1.7 Hz, 0.70 H), 7.56 (d, *J* = 8.1 Hz, 0.60 H), 7.52 (d, *J* = 8.1 Hz, 1.70 H), 7.50 – 7.42 (m, 3.70 H), 7.42 – 7.36 (m, 2 H), 5.71 (s, 1.40 H), 5.62 (s, 0.60 H), 2.59 (s, 0.90 H), 2.54 (s, 2.10 H).

^13^C NMR (150 MHz, CDCl_3_+TFA): δ = 167.1, 161.3, 160.3, 153.2, 152.3, 151.4, 141.6, 138.9, 135.4, 134.8, 133.7, 133.0, 132.2, 131.7, 131.58, 131.57, 131.5, 131.1, 130.7, 130.5, 130.4, 130.24, 130.2, 128.7, 128.4, 128.2, 127.2, 126.1, 124.5, 124.4, 124.2, 117.5, 116.5, 116.4, 55.3, 54.7, 22.4, 22.2.

HRMS (TOF ES^+^): *m*/*z* [M – ${\text{BF}}_{4}^{-}$]^+^ calcd for C_23_H_19_BrN^+^: 388.0695; found: 388.0701.

#### 1-Benzyl-5-Bromo-3-(4-Methoxylbenzylidene)-3*H*-Indolium Tetrafluoroborate (1e)

Red orange solid; yield 1.46 g (85% from 1.00 g 1-benzyl-5-bromo-1*H*-indole (**2b**)); mp 226-228°C (dec.).

IR (film): 3464, 3129, 2841, 1582, 1554, 1520, 1447, 1429, 1375, 1319, 1281, 1178, 1103, 1051, 888, 835, 799, 773, 716, 629, 595, 556 cm^−1^.

^1^H NMR (600 MHz, CDCl_3_+TFA; ~ 3.3:1 *Z*/*E* diastereomeric mixture): δ = 9.07 (s, 0.77 H), 8.73 (s, 0.23 H), 8.72 (s, 0.77 H), 8.68 (s, 0.23 H), 8.45 (d, *J* = 1.5 Hz, 0.23 H), 8.16 (d, *J* = 9.1 Hz, 0.46 H), ), 8.13 (d, *J* = 1.5 Hz, 0.77 H), ), 8.08 (d, *J* = 9.1 Hz, 1.54 H), 7.71 (dd, *J* = 8.6 Hz, 1.5 Hz, 0.23 H), 7.64 (dd, *J* = 8.6 Hz, 1.5 Hz, 0.77 H), 7.48 (d, *J* = 8.6 Hz, 0.23 H), 7.46 – 7.39 (m, 3.77 H), 7.38 – 7.31 (m, 2 H), 7.24 (d, *J* = 8.9 Hz, 0.46 H), 7.21 (d, *J* = 8.9 Hz, 0.46 H), 5.67 (s, 1.54 H), 5.57 (s, 0.46 H), 4.07 (s, 0.69 H), 4.04 (s, 2.31 H).

^13^C NMR (150 MHz, CDCl_3_+TFA): δ = 170.3, 169.3, 165.8, 159.9, 158.3, 150.6, 141.0, 139.5, 139.1, 138.1, 132.8, 132.0, 131.7, 131.2, 130.7, 130.6, 130.3, 130.1, 130.0, 128.4, 128.2, 128.1, 127.5, 127.1, 127.0, 126.5, 125.5, 123.8, 123.2, 123.0, 117.6, 116.8, 116.0, 115.9, 56.8, 56.7, 54.7, 54.1.

HRMS (TOF ES^+^): *m*/*z* [M – ${\text{BF}}_{4}^{-}$]^+^ calcd for C_23_H_19_BrNO^+^: 404.0645; found: 404.0651.

#### 1-Benzyl-5-Methoxy-3-(4-Methylbenzylidene)-3*H*-Indolium Tetrafluoroborate (1f)

Dark violet solid; yield 1.50 g (83% from 1.00 g 1-benzyl-5-methoxy-1*H*-indole (**2c**)); mp 212-214°C (dec.).

IR (film): 3130, 2947, 1619, 1587, 1526, 1482, 1444, 1372, 1298, 1263, 1237, 1189, 1099, 1051, 916, 858, 808, 754, 705, 645 cm^−1^.

^1^H NMR (600 MHz, CDCl_3_+TFA; ~ 2.7:1 *Z*/*E* diastereomeric mixture): δ = 8.89 (s, 0.73 H), 8.78 (s, 0.73 H), 8.64 (s, 0.27 H), 8.59 (s, 0.27 H), 7.95 (d, *J* = 8.1 Hz, 0.54 H), 7.87 (d, *J* = 2.5 Hz, 0.27 H), ), 7.82 (d, *J* = 8.1 Hz, 1.46 H), ), 7.54 – 7.51 (m, 1 H), 7.50 – 7.41 (m, 5.73 H), 7.41 – 7.34 (m, 2 H), 7.14 (dd, *J* = 9.1 Hz, 2.5 Hz, 0.27 H), 7.10 (dd, *J* = 9.1 Hz, 2.5 Hz, 0.73 H), 5.63 (s, 1.46 H), 5.55 (s, 0.54 H), 3.96 (s, 2.19 H), 3.89 (s, 0.81 H), 2.54 (s, 0.81 H), 2.50 (s, 2.19 H).

^13^C NMR (150 MHz, CDCl_3_+TFA): δ = 164.3, 158.9, 158.7, 151.3, 150.4, 149.6, 136.8, 134.6, 134.1, 133.8, 131.8, 131.4, 131.2, 131.0, 130.9, 130.8, 130.7, 130.6, 130.3, 130.2, 130.1, 130.0, 129.3, 128.6, 128.3, 127.3, 127.2, 127.0, 116.8, 116.2, 116.13, 116.09, 110.0, 106.0, 56.44, 56.36, 55.0, 54.4, 22.4, 22.2.

HRMS (TOF ES^+^): *m*/*z* [M – ${\text{BF}}_{4}^{-}$]^+^ calcd for C_24_H_22_NO^+^: 340.1696; found: 340.1701.

### Synthesis of Imidates 6

#### Three-Component Synthesis of Imidates 6; General Procedure (GP1)

An isocyanide **4** (0.65 mmol) was dissolved in a mixture of abs. alcohol (1 mL) and MeCN (1 mL). A salt **1** (0.5 mmol) and K_2_CO_3_ (0.1 g, 0.75 mmol) were then added. The reaction mixture was stirred at r.t. for 12 h and concentrated in vacuo. The residue was dissolved in EtOAc (50 mL), washed with NaHCO_3_ (2 × 25 mL), brine (20 mL), and dried over anhydrous Na_2_SO_4_. The EtOAc was evaporated in vacuo. The residue was chromatographed on a column with silica gel with EtOAc–hexane.

#### Consecutive Four-Component Synthesis of Imidates 6; General Procedure (GP2)

*N*-Alkylindole (0.5 mmol) and aromatic aldehyde (0.5 mmol) were dissolved in dry CH_2_Cl_2_ (3 mL). HBF_4_·OEt_2_ (0.1 ml, 0.75 mmol) was then added dropwise at 0–5°C over a period of 2 min. The reaction mixture was allowed to warm to r.t. and stirred for additional 20 min. Then K_2_CO_3_ (0.14 g, 1.0 mmol), anhydrous alcohol (3 ml) and isocyanide **4** (0.65 mmol) were added. The reaction mixture was stirred at r.t. for 12 h and concentrated in vacuo. The residue was dissolved in EtOAc (50 mL), washed with NaHCO_3_ (2 × 25 mL), brine (20 mL) and dried over anhydrous Na_2_SO_4_. The EtOAc was evaporated in vacuo. The residue was chromatographed on a column with silica gel with EtOAc–hexane.

#### Methyl 2-(1-Benzyl-1*H*-Indol-3-yl)-*N*-(4-Methoxyphenyl)-2-(*p-*tolyl)acetimidate (6a)

Yield, obtained by following GP1: 154 mg (65%).

Yellowish oil; *R*_*f*_ = 0.47 (EtOAc–hexane, 1:6).

IR (film): 3028, 2925, 2834, 1733, 1661, 1609, 1583, 1506, 1465, 1354, 1303, 1238, 1178, 1032, 909, 831, 740, 696, 636 cm^−1^.

^1^H NMR (600 MHz, CDCl_3_): δ = 7.36 – 7.23 (m, 5H), 7.19 – 7.13 (m, 3H), 7.12 – 7.07 (m, 4H), 7.05 – 6.99 (m, 2H), 6.84 (d, *J* = 9.1 Hz, 2H), 6.74 (d, *J* = 9.1 Hz, 2H), 5.42 (s, 1H), 5.37 – 5.29 (m, 2H), 3.84 (s, 3H), 3.80 (s,3H), 2.33 (s, 3H).

^13^C NMR (150 MHz, CDCl_3_): δ = 163.6, 155.8, 141.8, 137.7, 137.1, 136.7, 136.4, 129.1 (2C), 128.8 (2C), 128.4 (2C), 128.0, 127.7, 127.4, 126.7 (2C), 122.4 (2C), 122.0, 119.7, 119.4, 114.4 (2C), 114.0, 109.9, 55.6, 53.7, 50.1, 42.7, 21.2.

MS (ESI): *m*/z = 475 [M + H]^+^

HRMS (TOF ES^+^): *m*/*z* [M + H]^+^ calcd for C_32_H_31_N_2_${\text{O}}_{2}^{+}$: 475.2380; found: 475.2375.

#### Methyl 2-(1-Benzyl-1*H*-indol-3-yl)-*N*,2-bis(4-methoxyphenyl)acetimidate (6b)

Yield, obtained by following GP1: 206 mg (84%).

Yield, obtained by following GP2: 181 mg (74%).

Yellowish oil; *R*_*f*_ = 0.33 (EtOAc–hexane, 1:5).

IR (film): 3029, 2997, 2939, 2834, 1734, 1656, 1609, 1583, 1508, 1465, 1301, 1238, 1177, 1104, 1034, 908, 834, 741, 696, 636 cm^−1^.

^1^H NMR (600 MHz, CDCl_3_): δ = 7.36 – 7.23 (m, 5H), 7.21 – 7.14 (m, 3H), 7.12 (d, *J* = 7.0 Hz, 2H), 7.07 – 7.00 (m, 2H), 6.88 – 6.82 (m, 4H), 6.79 – 6.74 (m, 2H), 5.41 (s, 1H), 5.37 – 5.28 (m, 2H), 3.86 (s, 3H), 3.81 (s, 3H), 3.80 (s, 3H).

^13^C NMR (150 MHz, CDCl_3_): δ = 163.7, 158.5, 155.8, 141.8, 137.7, 136.78, 132.2, 129.6 (2C), 128.9 (2C), 128.0, 127.7, 127.4, 126.7 (2C), 122.4 (2C), 122.0, 119.7, 119.4, 114.5(2C), 114.3, 113.8 (2C), 109.9, 55.6, 55.3, 53.67, 50.2, 42.4.

MS (ESI): *m*/z = 491 [M + H]^+^

HRMS (TOF ES^+^): *m*/*z* [M + H]^+^ calcd for C_32_H_31_N_2_${\text{O}}_{3}^{+}$: 491.2329; found: 491.2337.

#### Methyl 2-(1-Benzyl-1*H*-Indol-3-yl)-*N*-(4-Methoxyphenyl)-2-Phenylacetimidate (6c)

Yield, obtained by following GP1: yield 129 mg (56%).

Yellowish oil; *R*_*f*_ = 0.43 (EtOAc–hexane, 1:5).

IR (film): 3058, 3027, 2931, 2833, 2391, 1735, 1662, 1605, 1506, 1466, 1334, 1301, 1238, 1178, 1031, 908, 833, 738, 698, 627 cm^−1^.

^1^H NMR (600 MHz, CDCl_3_): δ = 7.32 – 7.29 (m, 2H), 7.28 – 7.25 (m, 5H), 7.24 – 7.22 (m, 3H), 7.15 (dd, *J* = 8.0, 7.2 Hz, 1H), 7.10 (d, *J* = 7.6 Hz, 2H), 7.03 – 6.99 (m, 2H), 6.82 (d, *J* = 8.6 Hz, 2H), 6.73 (d, *J* = 8.6 Hz, 2H), 5.44 (s, 1H), 5.35 – 5.27 (m, 2H), 3.83 (s, 3H), 3.79 (s, 3H).

^13^C NMR (150 MHz, CDCl_3_): δ = 163.4, 155.8, 141.8, 140.2, 137.7, 136.7, 128.9 (2C), 128.5 (2C), 128.4 (2C), 128.1, 127.7, 127.4, 126.8, 126.7 (2C), 122.4 (2C), 122.0, 119.7, 119.4, 114.5 (2C), 113.8, 109.9, 55.6, 53.7, 50.2, 43.1.

MS (ESI): *m*/z = 461 [M + H]^+^

HRMS (TOF ES^+^): *m*/*z* [M + H]^+^ calcd for C_31_H_29_N_2_${\text{O}}_{2}^{+}$: 461.2224; found: 461.2217.

#### Methyl *N*-(4-Methoxyphenyl)-2-(1-Methyl-1*H*-Indol-3-yl)-2-(*p*-tolyl)acetimidate (6d)

Yield, obtained by following GP1: yield 101 mg (51%)

Brownish oil; *R*_*f*_ = 0.50 (EtOAc–hexane, 1:5).

IR (film): 3048, 3000, 2941, 2833, 1663, 1505, 1465, 1372, 1237, 1179, 1031, 909, 833, 773, 740, 644 cm^−1^.

^1^H NMR (600 MHz, CDCl_3_): δ = 7.29 (d, *J* = 8.1 Hz, 1H), 7.24 (d, *J* = 8.1 Hz, 1H), 7.22 – 7.18 (m, 1H), 7.12 (d, *J* = 8.0 Hz, 2H), 7.08 (d, *J* = 8.0 Hz, 2H), 7.01 (m, 1H), 6.89 (s, 1H), 6.83 (d, *J* = 8.9 Hz, 2H), 6.73 (d, *J* = 8.9 Hz, 2H), 5.37 (s, 1H), 3.84 (s, 3H), 3.79 (s,3H), 3.76 (s, 3H), 2.32 (s, 3H).

^13^C NMR (150 MHz, CDCl_3_): δ = 163.6, 155.8, 141.8, 137.3, 137.0, 136.3, 129.1 (2C), 128.39, 128.37 (2C), 127.1, 122.4 (2C), 121.7, 119.4, 119.1, 114.4 (2C), 113.4, 109.3, 55.6, 53.7, 42.6, 32.9, 21.2.

MS (ESI): *m*/z = 399 [M + H]^+^

HRMS (TOF ES^+^): *m*/*z* [M + H]^+^ calcd for C_26_H_27_N_2_${\text{O}}_{2}^{+}$: 399.2067; found: 399.2035.

#### Methyl *N*,2-bis(4-Methoxyphenyl)-2-(1-Methyl-1*H*-Indol-3-yl)acetimidate (6e)

Yield, obtained by following GP1: 180 mg (87%).

Yield, obtained by following GP2: 160 mg (77%).

Brownish oil; *R*_*f*_ = 0.37 (EtOAc–hexane, 1:5).

IR (film): 3048, 2998, 2943, 2834, 2391, 1737, 1661, 1609, 1583, 1508, 1464, 1440, 1329, 1239, 1178, 1032, 908, 834, 740, 634 cm^−1^.

^1^H NMR (600 MHz, CDCl_3_): δ = 7.29 (d, *J* = 8.1 Hz, 1H), 7.23 (d, *J* = 8.1 Hz, 1H), 7.22 −7.19 (m, 1H), 7.14 (d, *J* = 9.1 Hz, 2H), 7.04 – 6.99 (m, 1H), 6.87 (s, 1H), 6.82 (d, *J* = 8.9 Hz, 2H), 6.80 (d, *J* = 8.9 Hz, 2H), 6.72 (d, *J* = 9.1 Hz, 2H), 5.34 (s, 1H), 3.84 (s, 3H), 3.79 (s, 3H), 3.78 (s, 3H), 3.76 (s, 3H).

^13^C NMR (150 MHz, CDCl_3_): δ = 163.7, 158.4, 155.7, 141.8, 137.1, 132.4, 129.5 (2C), 128.3, 127.1, 122.4 (2C), 121.7, 119.4, 119.1, 114.4 (2C), 113.7 (2C), 113.6, 109.3, 55.6, 55.3, 53.7, 42.3, 32.9.

MS (ESI): *m*/z = 415 [M + H]^+^

HRMS (TOF ES^+^): *m*/*z* [M + H]^+^ calcd for C_26_H_27_N_2_${\text{O}}_{3}^{+}$: 415.2016; found: 415.2025.

#### Methyl *N*-(4-Methoxyphenyl)-2-(1-Methyl-1*H*-Indol-3-yl)-2-Phenylacetimidate (6f)

Yield, obtained by following GP1: 150 mg (78%).

Yield, obtained by following GP2: 131 mg (68%).

Brownish oil; *R*_*f*_ = 0.50 (EtOAc–hexane, 1:3).

IR (film): 3108, 2996, 2938, 2901, 2834, 1738, 1664, 1604, 1547, 1503, 1439, 1372, 1304, 1239, 1180, 1155, 1105, 1032, 905, 845, 799, 743, 718, 650 cm^−1^.

^1^H NMR (600 MHz, CDCl_3_): δ = 7.30 (d, *J* = 8.2 Hz, 1H), 7.29 – 7.25 (m, 2H), 7.24 – 7.19 (m, 5H), 7.01 (m, 1H), 6.90 (s, 1H), 6.83 (d, *J* = 8.6 Hz, 2H), 6.73 (d, *J* = 8.6 Hz, 2H), 5.41 (s, 1H), 3.85 (s, 3H), 3.79 (s, 3H), 3.76 (s, 3H).

^13^C NMR (150 MHz, CDCl_3_): δ = 163.4, 155.8, 141.8, 140.3, 137.1, 128.49 (2C), 128.45, 128.4 (2C), 127.1, 126.8, 122.4 (2C), 121.8, 119.4, 119.1, 114.4 (2C), 113.2, 109.3, 55.6, 53.7, 43.0, 32.9.

MS (ESI): *m*/z = 385 [M + H]^+^

HRMS (TOF ES^+^): *m*/*z* [M + H]^+^ calcd for C_25_H_25_N_2_${\text{O}}_{2}^{+}$: 385.1911; found: 385.1911.

#### Methyl 2-(4-(Dimethylamino)phenyl)-N-(4-Methoxyphenyl) -2-(1-Methyl-1H-indol-3-yl) acetimidate (6g)

Yield, obtained by following GP1: 109 mg (51%).

Yield, obtained by following GP2: 51 mg (24%).

Brownish oil; *R*_*f*_ = 0.44 (EtOAc–hexane, 1:5).

IR (film): 3059, 2927, 2852, 1759, 1703, 1661, 1602, 1512, 1464, 1351, 1237, 1157, 1031, 945, 825, 740, 607 cm^−1^.

^1^H NMR (600 MHz, CDCl_3_): δ = 7.29 – 7.26 (m, 2H), 7.22 – 7.18 (m, 1H), 7.12 (d, *J* = 8.6 Hz, 2H), 6.99 – 7.04 (m, 1H), 6.88 (s, 1H), 6.83 (d, *J* = 8.6 Hz, 2H), 6.74 (d*, J* = 8.6 Hz, 2H), 6.73 – 6.66 (m, 2H), 5.31 (s, 1H), 3.83 (s, 3H), 3.79 (s, 3H), 3.75 (s, 3H), 2.93 (s, 6H).

^13^C NMR (150 MHz, CDCl_3_): δ = 164.0, 155.8, 141.9, 137.1, 129.5, 129.3, 128.4 (2C), 127.2, 122.5 (2C), 121.8, 121.7, 119.5, 119.0, 114.4 (2C), 114.0, 112.8 (2C), 109.3, 55.6, 53.7, 42.2, 41.0 (2C), 32.9.

MS (ESI): *m*/z = 428 [M + H]^+^

HRMS (TOF ES^+^): *m*/*z* [M + H]^+^ calcd for C_27_H_30_N_3_${\text{O}}_{2}^{+}$: 428.2333; found: 428.2208.

#### Methyl 2-(1-Benzyl-5-Bromo-1H-Indol -3-yl)-N-(4-Methoxyphenyl)-2-(p-tolyl)acetimidate (6h)

Yield, obtained by following GP1: 130 mg (47%)

Brown oil; *R*_*f*_ = 0.47 (EtOAc–hexane, 1:7).

IR (film): 3030, 2941, 2833, 1733, 1661, 1607, 1505, 1468, 1282, 1237, 1176, 1103, 1033, 909, 874, 828, 793, 765, 732, 697, 641 cm^−1^.

^1^H NMR (600 MHz, CDCl_3_): δ = 7.36 (d, *J* = 2.0 Hz, 1H), 7.34 – 7.27 (m, 3H), 7.21 (dd, *J* = 8.6, 2.0 Hz, 1H), 7.12 – 7.07 (m, 5H), 7.06 (d, *J* = 7.7 Hz, 2H), 7.02 (s, 1H), 6.85 (d, *J* = 9.1 Hz, 2H), 6.71 (d, *J* = 9.1 Hz, 2H), 5.33 (s, 1H), 5.32 – 5.24 (m, 2H), 3.83 (s, 3H), 3.81 (s, 3H), 2.33 (s, 3H).

^13^C NMR (150 MHz, CDCl_3_): δ = 163.3, 155.9, 141.6, 137.2, 136.7, 136.6, 135.3, 129.3 (2C), 129.2, 129.1, 128.9 (2C), 128.3 (2C), 127.9, 126.6 (2C), 124.9, 122.4 (2C), 122.3, 114.5 (2C), 113.6, 112.9, 111.5, 55.6, 53.8, 50.4, 42.5, 21.2.

MS (ESI): *m*/z = 553 [M + H]^+^

HRMS (TOF ES^+^): *m*/*z* [M + H]^+^ calcd for C_32_H_30_BrN_2_${\text{O}}_{2}^{+}$: 553.1485; found: 553.1505.

#### Methyl 2-(1-Benzyl-5-Bromo-1H -Indol-3-yl)-N,2-bis(4-Methoxyphenyl)acetimidate (6i)

Yield, obtained by following GP1: 148 mg (52%).

Yield, obtained by following GP2: 114 mg (40%).

Brownish oil; *R*_*f*_ = 0.60 (EtOAc–hexane, 1:5).

IR (film): 3030, 2996, 2943, 2834, 1734, 1662, 1608, 1508, 1467, 1354, 1238, 1176, 1104, 1033, 908, 832, 734, 697, 634 cm^−1^.

^1^H NMR (600 MHz, CDCl_3_): δ = 7.37 (d, *J* = 1.8 Hz, 1H), 7.33 – 7.25 (m, 3H), 7.20 (dd, *J* = 8.6, 1.8 Hz, 1H), 7.13 (d, *J* = 8.6 Hz, 2H), 7.08 (d, *J* = 8.6 Hz, 1H), 7.06 – 7.03 (m, 2H), 7.00 (s, 1H), 6.84 (d, *J* = 8.6 Hz, 2H), 6.82 (d, *J* = 9.1 Hz, 2H), 6.71 (d, *J* = 9.1 Hz, 2H), 5.31 (s, 1H), 5.31 – 5.23 (m, 2H), 3.82 (s, 3H), 3.80 (s, 3H), 3.78 (s, 3H).

^13^C NMR (150 MHz, CDCl_3_): δ = 163.3, 158.6, 155.9, 141.6, 137.2, 135.4, 131.7, 129.4 (2C), 129.1, 129.0, 128.9 (2C), 127.9, 126.6 (2C), 124.9, 122.3 (2C), 122.2, 114.5 (2C), 113.9 (2C), 113.8, 112.9, 111.4, 55.6, 55.3, 53.7, 50.4, 42.1.

MS (ESI): *m*/z = 569 [M + H]^+^

HRMS (TOF ES^+^): *m*/*z* [M + H]^+^ calcd for C_32_H_30_BrN_2_${\text{O}}_{3}^{+}$: 569.1434; found: 569.1412.

#### Methyl 2-(1-Benzyl-5-Methoxy-1H-Indol-3-yl)-N-(4-Methoxyphenyl)-2-(p-tolyl)acetimidate (6j)

Yield, obtained by following GP1: 179 mg (71%).

Yield, obtained by following GP2: 141 mg (56%).

Brown oil; yield; *R*_*f*_ = 0.56 (EtOAc–hexane, 1:5).

IR (film): 3030, 2994, 2939, 2833, 1734, 1661, 1621, 1578, 1506, 1487, 1452, 1288, 1238, 1176, 1103, 1030, 901, 830, 774, 704 cm^−1^.

^1^H NMR (600 MHz, CDCl_3_): δ = 7.31 – 7.25 (m, 3H), 7.15 (d, *J* = 8.1 Hz, 2H), 7.12 – 7.05 (m, 5H), 6.97 (s, 1H), 6.83 (d, *J* = 9.1 Hz, 2H), 6.79 (dd, *J* = 9.1, 2.5 Hz, 1H), 6.75 (d, *J* = 9.1 Hz, 2H), 6.70 (d, *J* = 2.5 Hz, 1H), 5.36 (s, 1H), 5.30 – 5.22 (m, 2H), 3.83 (s, 3H), 3.79 (s, 3H), 3.72 (s, 3H), 2.33 (s, 3H).

^13^C NMR (150 MHz, CDCl_3_): δ = 163.6, 155.8, 154.0, 141.9, 137.8, 137.80, 137.1, 136.4, 131.9, 129.1 (2C), 128.8 (2C), 128.6, 128.4 (2C), 127.8, 126.6 (2C), 122.5 (2C), 114.4 (2C), 113.5, 112.2, 110.7, 101.2, 55.8, 55.6, 53.7, 50.4, 42.6, 21.2.

MS (ESI): *m*/z = 505 [M + H]^+^

HRMS (TOF ES^+^): *m*/*z* [M + H]^+^ calcd for C_33_H_33_N_2_${\text{O}}_{3}^{+}$: 505.2486; found: 505.2461.

#### Ethyl 2-(1-Benzyl-1H-Indol-3-yl)-N-(4-Methoxyphenyl)-2-(p-tolyl)acetimidate (6k)

Yield, obtained by following GP1: 129 mg (53%)

Brownish oil; *R*_*f*_ = 0.71 (EtOAc–hexane, 1:5).

IR (film): 3030, 2976, 2946, 2901, 2833, 1732, 1656, 1610, 1505, 1466, 1357, 1334, 1306, 1236, 1178, 1103, 1036, 958, 834, 741, 696 cm^−1^.

^1^H NMR (600 MHz, CDCl_3_): δ = 7.34 – 7.23 (m, 5H), 7.17 – 7.13 (m, 3H), 7.11 (d, *J* = 8.1 Hz, 2H), 7.08 (d, *J* = 8.1 Hz, 2H), 7.05 (s, 1H), 6.99 – 7.00 (m, 1H), 6.83 (d, *J* = 9.1 Hz, 2H), 6.73 (d, *J* = 9.1 Hz, 2H), 5.40 (s, 1H), 5.31 (s, 2H), 4.34 – 4.27 (m, 2H), 3.80 (s, 3H), 2.33 (s, 3H), 1.26 (t, *J* = 7.1 Hz, 3H).

^13^C NMR (150 MHz, CDCl_3_): δ = 162.9, 155.7, 142.1, 137.7, 137.4, 136.7, 136.2, 129.0 (2C), 128.8 (2C), 128.4 (2C), 127.9, 127.7, 127.5, 126.8 (2C), 122.4 (2C), 121.9, 119.8, 119.2, 114.4 (2C), 114.2, 109.7, 61.8, 55.6, 50.1, 42.6, 21.1, 14.2.

MS (ESI): *m*/z = 489 [M + H]^+^

HRMS (TOF ES^+^): *m*/*z* [M + H]^+^ calcd for C_33_H_33_N_2_${\text{O}}_{2}^{+}$: 489.2537; found: 489.2553.

#### Ethyl 2-(1-Benzyl-1H-Indol-3-yl)-N,2-bis(4-Methoxyphenyl)acetimidate (6l)

Yield, obtained by following GP1: 118 mg (47%).

Yield, obtained by following GP2: 93 mg (37%).

Brownish oil; *R*_*f*_ = 0.71 (EtOAc–hexane, 1:3).

IR (film): 3031, 2930, 2834, 1727, 1658, 1609, 1508, 1465, 1301, 1238, 1177, 1104, 1036, 958, 834, 741, 696, 637 cm^−1^.

^1^H NMR (600 MHz, CDCl_3_): δ = 7.32 – 7.28 (m, 2H), 7.27 – 7.25 (m, 2H), 7.23 (d, *J* = 8.6 Hz, 1H), 7.16 (d, *J* = 8.4 Hz, 2H), 7.16 – 7.12 (m, 1H), 7.09 (d, *J* = 8.1 Hz, 2H), 7.02 (s, 1H), 7.02 – 6.98 (m, 1H), 6.81 (d, *J* = 8.6 Hz, 2H), 6.79 (d, *J* = 8.6 Hz, 2H), 6.71 (d, *J* = 8.6 Hz, 2H), 5.35 (s, 1H), 5.30 (s, 2H), 4.32 – 4.21 (m, 2H), 3.79 (s, 3H), 3.77 (s, 3H), 1.24 (t, *J* = 7.1 Hz, 3H).

^13^C NMR (150 MHz, CDCl_3_): δ = 163.0, 158.4, 155.7, 142.0, 137.7, 136.7, 132.5, 129.5 (2C), 128.8 (2C), 127.8, 127.7, 127.4, 126.7 (2C), 122.4 (2C), 121.9, 119.8, 119.2, 114.4 (2C), 114.3, 113.7 (2C), 109.8, 61.8, 55.6, 55.3, 50.1, 42.3, 14.2.

MS (ESI): *m*/z = 505 [M + H]^+^

HRMS (TOF ES^+^): *m*/*z* [M + H]^+^ calcd for C_33_H_33_N_2_${\text{O}}_{3}^{+}$: 505.2486; found: 505.2506.

#### Isopropyl 2-(1-Benzyl-1*H*-Indol-3-yl)-*N*,2-bis(4-Methoxyphenyl)acetimidate (6m)

Yield, obtained by following GP1: 145 mg (56%).

Yield, obtained by following GP2: 119 mg (46%).

Brownish oil; yield; *R*_*f*_ = 0.43 (EtOAc–hexane, 1:8).

IR (film): 3056, 2933, 2837, 2736, 1683, 1654, 1600, 1577, 1509, 1465, 1302, 1250, 1160, 1178, 1108, 1030, 976, 834, 742, 698 cm^−1^.

^1^H NMR (600 MHz, CDCl_3_): δ = 7.35 – 7.29 (m, 2H), 7.28 – 7.25 (m, 2H), 7.23 (d, *J* = 8.2 Hz, 1H), 7.15 (d, *J* = 9.1 Hz, 2H), 7.13 – 7.08 (m, 3H), 7.03 (s, 1H), 7.02 – 6.98 (m, 1H), 6.81 (d*, J* = 8.6 Hz, 2H), 6.78 (d, *J* = 8.6 Hz, 2H), 6.70 (d*, J* = 9.1 Hz, 2H), 5.34 (s, 1H), 5.33 – 5.26 (m, 2H), 5.23 – 5.18 (m, 1H), 3.79 (s, 3H), 3.77 (s, 3H), 1.22 (d, *J* = 6.2 Hz, 3H), 1.17 (d, *J* = 6.2 Hz, 3H).

^13^C NMR (150 MHz, CDCl_3_): δ = 162.1, 158.3, 155.6, 142.2, 137.7, 136.6, 132.7, 129.5 (2C), 128.8 (2C), 127.8, 127.7, 127.5, 126.8 (2C), 126.5, 122.3 (2C), 121.8, 119.8, 119.1, 114.4 (2C), 113.6 (2C), 109.7, 68.0, 55.6, 55.3, 50.1, 42.2, 21.7, 21.6.

MS (ESI): *m*/z = 519 [M + H]^+^

HRMS (TOF ES^+^): *m*/*z* [M + H]^+^ calcd for C_34_H_35_N_2_${\text{O}}_{3}^{+}$: 519.2642; found: 519.2623.

#### Methyl 2-(1-Benzyl-1*H*-Indol-3-yl)-*N*-Phenyl-2-(*p*-tolyl)acetimidate (6n)

Yield, obtained by following GP1: 113 mg (51%).

Yield, obtained by following GP2: 91 mg (41%).

Orange oil; *R*_*f*_ = 0.41 (EtOAc–hexane, 1:10).

IR (film): 3055, 3028, 2941, 2861, 1665, 1595, 1546, 1511, 1466, 1334, 1249, 1178, 1072, 1017, 908, 834, 740, 695 cm^−1^.

^1^H NMR (600 MHz, CDCl_3_): δ = 7.34 – 7.29 (m, 2H), 7.28 – 7.25 (m, 3H), 7.25 – 7.22 (m, 2H), 7.15 – 7.11 (m, 3H), 7.10 – 7.03 (m, 5H), 7.02 – 6.98 (m, 2H), 6.79 (dd, *J* = 8.4, 1.1 Hz, 2H), 5.35 (s, 1H), 5.34 – 5.27 (m, 2H), 3.84 (s, 3H), 2.32 (s, 3H).

^13^C NMR (150 MHz, CDCl_3_): δ = 163.1, 148.6, 137.7, 137.0, 136.7, 136.40, 129.1 (2C), 129.1 (2C), 128.8 (2C), 128.4 (2C), 128.0, 127.6, 127.4, 126.7 (2C), 123.1, 121.9, 121.5 (2C), 119.7, 119.4, 113.9, 109.9, 53.7, 50.1, 42.9, 21.2.

MS (ESI): *m*/z = 445 [M + H]^+^

HRMS (TOF ES^+^): *m*/*z* [M + H]^+^ calcd for C_31_H_29_N_2_O^+^: 445.2274; found: 445.2250.

#### Methyl 2-(1-Benzyl-1*H*-Indol-3-yl)-2-(4-Methoxyphenyl)-*N*-Phenylacetimidate (6o)

Yield, obtained by following GP1: 145 mg (63%).

Orange oil; *R*_*f*_ = 0.66 (EtOAc–hexane, 1:5).

IR (film): 3030, 2928, 2837, 1738, 1664, 1594, 1510, 1465, 1303, 1247, 1175, 1031, 740 cm^−1^.

^1^H NMR (600 MHz, CDCl_3_): δ = 7.33 −7.29 (m, 2H), 7.28 – 7.21 (m, 5H), 7.16 – 7.12 (m, 3H), 7.10 – 7.07 (m, 2H), 7.07 – 7.03 (m, 1H), 7.00 (ddd, *J* = 8.1, 7.0, 1.0 Hz, 1H), 6.97 (s, 1H), 6.80 (d, *J* = 8.6 Hz, 2H), 6.79 (d, *J* = 8.6 Hz, 2H), 5.34 – 5.26 (m, 3H), 3.83 (s, 3H), 3.78 (s, 3H).

^13^C NMR (150 MHz, CDCl_3_): δ = 163.1, 158.5, 148.6, 137.7, 136.8, 132.1, 129.5 (2C), 129.1 (2C), 128.8 (2C), 127.9, 127.6, 127.3, 126.7 (2C), 123.1, 122.0, 121.5 (2C), 119.7, 119.4, 114.2, 113.8 (2C), 109.9, 55.3, 53.7, 50.1, 42.6.

MS (ESI): *m*/z = 461 [M + H]^+^

HRMS (TOF ES^+^): *m*/*z* [M + H]^+^ calcd for C_31_H_29_N_2_${\text{O}}_{2}^{+}$: 461.2224; found: 461.2239.

#### Methyl 2-(1-Methyl-1*H*-Indol-3-y l)-*N*-Phenyl-2-(*p*-tolyl)acetimidate (6p)

Yield, obtained by following GP1: 100 mg (54%)

Orange oil; *R*_*f*_ = 0.57 (EtOAc–hexane, 1:5).

IR (film): 3052, 3021, 2952, 2869, 2854, 1737, 1662, 1595, 1511, 1461, 1375, 1330, 1247, 1153, 1023, 900, 740, 698 cm^−1^.

^1^H NMR (600 MHz, CDCl_3_): δ = 7.30 – 7.26 (m, 3H), 7.23 – 7.17 (m, 2H), 7.12 (d, *J* = 8.1 Hz, 2H), 7.10 – 7.04 (m, 3H), 7.00 (ddd, *J* = 8.1, 7.0, 1.0 Hz, 1H), 6.88 (s, 1H), 6.79 (dd, *J* = 8.4, 1.2 Hz, 2H), 5.32 (s, 1H), 3.85 (s, 3H), 3.76 (s, 3H), 2.32 (s, 3H).

^13^C NMR (150 MHz, CDCl_3_): δ = 163.1, 148.6, 137.2, 137.0, 136.4, 129.1 (2C), 129.1 (2C), 128.4, 128.35 (2C), 127.1, 123.1, 121.7, 121.5 (2C), 119.4, 119.1, 113.3, 109.3, 53.8, 42.8, 32.9, 21.2.

MS (ESI): *m*/z = 369 [M + H]^+^

HRMS (TOF ES^+^): *m*/*z* [M + H]^+^ calcd for C_25_H_25_N_2_O^+^: 369.1961; found: 369.1961.

#### Methyl 2-(1-Benzyl-1*H*-Indol-3-yl)-*N*-(4-Chlorophenyl)-2-(4-Methoxyphenyl)acetimidate (6q)

Yield, obtained by following GP1: 106 mg (43%).

Yield, obtained by following GP2: 89 mg (36%).

Yellowish oil; yield; *R*_*f*_ = 0.53 (EtOAc–hexane, 1:5).

IR (film): 3028, 2948, 2836, 1736, 1666, 1610, 1510, 1466, 1302, 1242, 1176, 1092, 1031, 908, 835, 739 cm^−1^.

^1^H NMR (600 MHz, CDCl_3_): δ = 7.32 – 7.23 (m, 5H), 7.22 (d, *J* = 8.6 Hz, 2H), 7.19 – 7.12 (m, 3H), 7.08 (d, *J* = 7.2 Hz, 2H), 7.02 (ddd, *J* = 7.9, 7.0, 0.9 Hz, 1H), 6.95 (s, 1H), 6.81 (d, *J* = 9.1 Hz, 2H), 6.71 (d, *J* = 8.6 Hz, 2H), 5.34 – 5.22 (m, 3H), 3.83 (s, 3H), 3.78 (s, 3H).

^13^C NMR (150 MHz, CDCl_3_): δ = 163.1, 158.5, 147.2, 137.7, 136.8, 132.1, 129.5 (2C), 129.1 (2C), 128.8 (2C), 127.9, 127.6, 127.3, 126.7 (2C), 123.1, 121.95, 121.5 (2C), 119.7, 119.4, 114.2, 113.8 (2C), 109.9, 55.3, 53.7, 50.1, 42.6.

MS (ESI): *m*/z = 495 [M + H]^+^

HRMS (TOF ES^+^): *m*/*z* [M + H]^+^ calcd for C_31_H_28_ClN_2_${\text{O}}_{2}^{+}$: 495.1834; found: 495.1817.

#### Methyl 2-(1-Benzyl-5-Methoxy-1*H*-Indol-3-yl)-(4-Chlorophenyl)-2-(*p*-tolyl)acetimidate (6r)

Yield, obtained by following GP1: 94 mg (37%)

Brownish oil; *R*_*f*_ = 0.49 (EtOAc–hexane, 1:5).

IR (film): 3027, 2992, 2941, 2919, 2833, 1661, 1590, 1511, 1487, 1452, 1398, 1355, 1241, 1213, 1175, 1094, 1028, 903, 869, 829, 796, 732, 704, 643 cm^−1^.

^1^H NMR (600 MHz, CDCl_3_): δ = 7.31 – 7.28 (m, 2H), 7.28 – 7.25 (m, 1H), 7.23 (d, *J* = 8.6 Hz, 2H), 7.14 – 7.05 (m, 7H), 6.95 (s, 1H), 6.80 (dd, *J* = 8.6, 2.5 Hz, 1H), 6.73 (d, *J* = 8.6 Hz, 2H), 6.65 (d, *J* = 2.5 Hz, 1H), 5.29 – 5.22 (m, 3H), 3.83 (s, 3H), 3.72 (s, 3H), 2.32 (s, 3H).

^13^C NMR (150 MHz, CDCl_3_): δ = 163.7, 154.0, 147.3, 137.7, 136.7, 136.58, 131.9, 129.2 (2C), 129.1 (2C), 128.8 (2C), 128.6, 128.4, 128.3 (2C), 127.7, 127.7, 126.6 (2C), 122.9 (2C), 113.0, 112.3, 110.7, 101.0, 55.8, 53.9, 50.4, 42.9, 21.2.

MS (ESI): *m*/z = 509 [M + H]^+^

HRMS (TOF ES^+^): *m*/*z* [M + H]^+^ calcd for C_32_H_30_ClN_2_${\text{O}}_{2}^{+}$: 509.1990; found: 509.1962.

#### 1-Benzyl-3-(Methoxy(*p*-tolyl)Methyl)-1*H*-Indole (7)

According to GP1 without an isocyanide addition compound **7** (155 mg, 91%) was obtained as an orange oil; *R*_*f*_ = 0.71 (EtOAc–hexane, 1:5).

IR (film): 3029, 2922, 2816, 1717, 1613, 1550, 1511, 1495, 1466, 1453, 1355, 1335, 1306, 1249, 1174, 1084, 1028, 956, 810, 777, 740, 695, 639, 573 cm^−1^.

^1^H NMR (600 MHz, CDCl_3_): δ = 7.63 (d, *J* = 8.1 Hz, 1H), 7.37 (d, *J* = 8.1 Hz, 2H), 7.29 – 7.23 (m, 3H), 7.22 (d, *J* = 8.1 Hz, 1H), 7.18 – 7.12 (m, 3H), 7.09 – 7.05 (m, 3H), 6.88 (s, 1H), 5.56 (s, 1H), 5.28 – 5.20 (m, 2H), 3.42 (s, 3H), 2.34 (s, 3H).

^13^C NMR (150 MHz, CDCl_3_): δ = 138.8, 137.6, 137.161, 129.1, 128.8, 127.6, 127.3, 127.2, 127.1, 126.7, 122.1, 120.2, 119.6, 117.2, 109.9, 79.6, 56.7, 50.1, 21.3.

MS (EI, 70 eV): *m*/z (%) = 341 (11) [M]^+^, 310 (63) [M – OCH_3_]^+^, 250 (7), 218 (6), 204 (6), 91 [C_7_H_7_]^+^ (100).

### Synthesis of Amides 8

#### General Procedure

A salt **1** (0.5 mmol) and K_2_CO_3_ (100 mg, 0.75 mmol) were added to a solution of 0.65 mmol of isonitrile in a mixture of MeOH (1 mL), MeCN (1 mL) and H_2_O (0.2 mL). The reaction mixture was stirred at room temperature for 12 h. It was then diluted with 40 ml of EtOAc, washed with H_2_O (2 × 25 mL), brine (20 mL) and dried over anhydrous Na_2_SO_4_. The EtOAc was evaporated in vacuo. The residue was chromatographed on a column of silica gel with EtOAc–hexane.

#### *N*-Benzyl-2-(1-Benzyl-1*H*-Indol-3-yl)-2-(*p*-tolyl)acetamide (8a)

White solid; yield 132 mg (59%); mp 176–178°C; *R*_*f*_ = 0.42 (EtOAc–hexane, 1:3).

IR (film): 3299, 3087, 3029, 2949, 2920, 2874, 1645, 1612, 1532, 1496, 1433, 1356, 1334, 1222, 1030, 807, 768, 745, 696, 649 cm^−1^.

^1^H NMR (600 MHz, CDCl_3_): δ = 7.44 (d, *J* = 8.1 1H), 7.27 – 7.21 (m, 9H), 7.17 – 7.11 (m, 5H), 7.07 – 7.03 (m, 3H), 6.95 (d, *J* = 0.8 Hz, 1H), 6.12 (dd, *J* = 6.0, 5.7 Hz, 1H), 5.23 (s, 2H), 5.15 (s, 1H), 4.50 (dd, *J* = 15.1, 6.0 Hz, 1H), 4.41 (dd, *J* = 15.1, 5.7 Hz, 1H), 2.32 (s, 3H).

^13^C NMR (150 MHz, CDCl_3_): δ = 172.5, 138.4, 137.5, 137.0, 136.9, 136.5, 129.5 (2C), 128.8 (2C), 128.7 (2C), 128.5 (2C), 127.9, 127.7 (2C), 127.7, 127.5, 127.4, 126.7 (2C), 122.4, 119.8, 119.5, 114.0, 110.1, 50.9, 50.2, 43.8, 21.2.

MS (ESI): *m*/z = 445 [M + H]^+^

HRMS (TOF ES^+^): *m*/*z* [M + H]^+^ calcd for C_31_H_29_N_2_O^+^: 445.2274; found: 445.2301.

#### *N*-Benzyl-2-(1-Benzyl-1*H*-Indol-3-yl)-2-(4-Methoxyphenyl)acetamide (8b)

White solid; yield 144 mg (63%); mp 177–179°C; *R*_*f*_ = 0.41 (EtOAc–hexane, 1:3).

IR (film): 3328, 3062, 3028, 2930, 2837, 1735, 1642, 1610, 1510, 1466, 1354, 1303, 1243, 1179, 1029, 734, 698 cm^−1^.

^1^H NMR (600 MHz, CDCl_3_): δ = 7.42 (d, *J* = 8.1, 1H), 7.28 (d, *J* = 8.6 Hz, 2H), 7.26 – 7.21 (m, 7H), 7.19 – 7.13 (m, 3H), 7.07 – 7.02 (m, 3H), 6.95 (s, 1H), 6.85 (d, *J* = 8.6 Hz, 2H), 6.12 (dd, *J* = 6.1, 5.6 Hz, 1H), 5.24 (s, 2H), 5.13 (s, 1H), 4.50 (dd, *J* = 14.9, 6.1 Hz, 1H), 4.42 (dd, *J* = 14.9, 5.6 Hz, 1H), 3.78 (s, 3H).

^13^C NMR (150 MHz, CDCl_3_): δ = 172.6, 158.8, 138.4, 137.4, 137.0, 131.63, 129.7 (2C), 128.9 (2C), 128.7 (2C), 127.9, 127.7 (2C), 127.7, 127.4, 127.4, 126.7 (2C), 122.4, 119.8, 119.5, 114.2 (2C), 114.1, 110.1, 55.4, 50.5, 50.2, 43.8.

MS (ESI): *m*/z = 461 [M + H]^+^

HRMS (TOF ES^+^): *m*/*z* [M + H]^+^ calcd for C_31_H_29_N_2_${\text{O}}_{2}^{+}$: 461.2224; found: 461.2243.

#### *N*-Benzyl-2-(4-Methoxyphenyl)-2-(1-Methyl-1*H*-Indol-3-yl)acetamide (8c)

White solid; yield 101 mg (53%); mp 207–209°C; *R*_*f*_ = 0.26 (EtOAc–hexane, 1:3).

IR (film): 3280, 3088, 3006, 2920, 2835, 1647, 1607, 1557, 1507, 1473, 1419, 1342, 1248, 1223, 1174, 1031, 753 cm^−1^.

^1^H NMR (600 MHz, CDCl_3_): δ = 7.42 (d, *J* = 7.6, Hz, 1H), 7.30 – 7.20 (m, 7H), 7.18 −7.14 (m, 2H), 7.07 (ddd, *J* = 8.1, 7.0, 1.0 Hz, 1H), 6.86 (d, *J* = 9.1 Hz, 2H), 6.76 (s, 1H), 6.19 (dd, *J* = 6.1, 5.6 Hz, 1H), 5.12 (s, 1H), 4.51 (dd, *J* = 14.9, 6.1 Hz, 1H), 4.41 (dd, *J* = 14.9, 5.6 Hz, 1H), 3.79 (s, 3H), 3.70 (s, 3H)

^13^C NMR (150 MHz, CDCl_3_): δ = 173.0, 158.8, 138.3, 137.4, 131.6, 129.7 (2C), 128.7 (2C), 128.5, 127.8 (2C), 127.5, 127.1, 122.2, 119.5, 119.3, 114.2 (2C), 113.3, 109.5, 55.4, 50.3, 43.8, 32.9.

MS (ESI): *m*/z = 385 [M + H]^+^

HRMS (TOF ES^+^): *m*/*z* [M + H]^+^ calcd for C_25_H_24_N_2_${\text{O}}_{2}^{+}$: 385.1911; found: 385.1919.

#### *N*-Benzyl-2-(1-Methyl-1*H*-indol-3-yl)-2-Phenylacetamide (8d)

White solid; yield 88 mg (50%); mp 146–148°C; *R*_*f*_ = 0.29 (EtOAc–hexane, 1:3).

IR (film): 3256, 3050, 2940, 2098, 1736, 1634, 1542, 1475, 1451, 1372, 1335, 1216, 1152, 1111, 1029, 990, 745, 699, 584 cm^−1^.

^1^H NMR (600 MHz, CDCl_3_): δ = 7.44 (d, *J* = 8.1 Hz, 1H), 7.38 – 7.36 (m, 2H), 7.35 – 7.31 (m, 2H), 7.30 – 7.21 (m, 6H), 7.19 – 7.15 (m, 2H), 7.08 (ddd, *J* = 8.1, 7.0, 1.0 Hz, 1H), 6.77 (d, *J* = 1.0 Hz, 1H), 6.18 (dd, *J* = 6.1, 5.7 Hz, 1H), 5.16 (s, *J* = 6.8 Hz, 1H), 4.52 (dd, *J* = 15.5, 6.1 Hz, 1H), 4.42 (dd, *J* = 15.1, 5.7 Hz, 1H), 3.70 (s, 3H).

^13^C NMR (150 MHz, CDCl_3_): δ = 172.4, 139.7, 138.4, 137.3, 128.8 (2C), 128.7 (2C), 128.6 (2C), 128.6, 127.8 (2C), 127.5, 127.2, 127.1, 122.2, 119.6, 119.3, 113.1, 109.5, 51.2, 43.8, 32.9.

MS (ESI): *m*/z = 355 [M + H]^+^

HRMS (TOF ES^+^): *m*/*z* [M + H]^+^ calcd for C_24_H_23_N_2_O^+^: 355.1805; found: 355.1808.

#### *N*-Benzyl-2-(1-Benzyl-5-Bromo-1*H*-Indol-3-yl)-2-(*p*-tolyl)acetamide (8e)

White solid; yield 132 mg (51%); mp 184–186°C; *R*_*f*_ = 0.15 (EtOAc–hexane, 1:5).

IR (film): 3250, 3068, 3029, 2923, 1645, 1560, 1511, 1469, 1356, 1234, 1173, 1021, 869, 817, 802, 733, 697 cm^−1^.

^1^H NMR (600 MHz, CDCl_3_): δ = 7.55 (d, *J* = 2.0 Hz, 1H), 7.30 – 7.25 (m, 8H), 7.22 (dd, *J* = 8.6, 2.0 Hz, 1H) 7.17 (d, *J* = 7.1 Hz, 2H), 7.13 (d, *J* = 8.0 Hz, 2H), 7.06 (d, *J* = 8.6 Hz, 1H), 7.01 (dd, *J* = 7.6, 1.9 Hz, 2H), 7.00 (s, 1H), 6.07 – 6.02 (m, 1H), 5.21 (s, 2H), 5.05 (s, 1H), 4.52 – 4.43 (m, 2H), 2.32 (s, 3H).

^13^C NMR (150 MHz, CDCl_3_): δ = 172.1, 138.2, 137.2, 137.0, 136.0, 135.6, 129.7 (2C), 129.2, 129.1, 128.9 (2C), 128.8 (2C), 128.4 (2C), 127.9, 127.7 (2C), 127.5, 126.6 (2C), 125.2, 122.0, 113.5, 113.2, 111.6, 50.6, 50.5, 43.9, 21.2.

MS (ESI): *m*/z = 523 [M + H]^+^

HRMS (TOF ES^+^): *m*/*z* [M + H]^+^ calcd for C_32_H_28_BrN_2_O^+^: 523.1380; found: 523.1398.

#### *N*-Benzyl-2-(1-Benzyl-5-Methoxy-1*H*-Indol-3-yl)-2-(*p*-tolyl)acetamide (8f)

White solid; yield 135 mg (57%); mp 168–170°C; *R*_*f*_ = 0.13 (EtOAc–hexane, 1:5).

IR (film): 3283, 3031, 2923, 1649, 1622, 1489, 1453, 1352, 1228, 1171, 1032, 918, 822, 794, 742, 699 cm^−1^.

^1^H NMR (600 MHz, CDCl_3_): δ = 7.26 (d, *J* = 8.1 Hz, 2H), 7.25 – 7.21 (m, 6H), 7.16 (dd, *J* = 7.6, 2.0 Hz, 2H), 7.13 (d, *J* = 8.1 Hz, 2H), 7.09 (d, *J* = 8.6 Hz, 1H), 6.99 – 7.04 (m, 2H), 6.87 (s, 1H), 6.86 (d, *J* = 2.4 Hz, 1H), 6.80 (dd*, J* = 8.9, 2.4 Hz, 1H), 6.19 (dd, *J* = 6.1, 5.8 Hz, 1H), 5.19 (s, 2H), 5.11 (s, 1H), 4.51 (dd, *J* = 15.1, 6.1 Hz, 1H), 4.43 (dd, *J* = 15.1, 5.8 Hz, 1H), 3.72 (s, 3H), 2.33 (s, 3H).

^13^C NMR (150 MHz, CDCl_3_): δ = 172.5, 154.3, 138.4, 137.5, 136.9, 136.5, 132.2, 129.5 (2C), 128.8 (2C), 128.7 (2C), 128.6, 128.5 (2C), 127.8, 127.7 (2C), 127.7, 127.4, 126.6 (2C), 113.5, 112.7, 111.0, 101.0, 55.9, 50.9, 50.4, 43.8, 21.2.

MS (ESI): *m*/z = 475 [M + H]^+^

HRMS (TOF ES^+^): *m*/*z* [M + H]^+^ calcd for C_32_H_31_N_2_${\text{O}}_{2}^{+}$: 475.2380; found: 475.2399.

#### 2-(1-Benzyl-1*H*-Indol-3-yl)-*N*,2-bis(4-Methoxyphenyl)acetamide (8g)

White solid; yield 105 mg (44%); mp 208–210°C; *R*_*f*_ = 0.57 (EtOAc–hexane, 1:3).

IR (film): 3287, 3255, 3196, 3134, 3058, 3024, 2962, 2835, 1655, 1606, 1546, 1509, 1469, 1410, 1346, 1303, 1251, 1171, 1031, 969, 833, 794, 746, 730, 700 cm^−1^.

^1^H NMR (600 MHz, DMSO-*d*_6_): δ = 10.20 (s, 1H), 7.53 (d, *J* = 9.1 Hz, 2H), 7.44 (s, 1H), 7.43 – 7.34 (m, 4H), 7.32 – 7.27 (m, 2H), 7.25 – 7.21 (m, 1H), 7.18 (d*, J* = 7.1 Hz, 2H), 7.09 – 7.04 (m, 1H), 6.99 – 6.95 (m, 1H), 6.90 – 6.84 (m, 4H), 5.41 (s, 2H), 5.26 (s, 1H), 3.71 (s, 3H) 3.70 (s, 3H).

^13^C NMR (150 MHz, DMSO-*d*_6_): δ = 170.2, 158.1, 155.2, 138.4, 136.1, 132.4, 132.3, 129.3 (2C), 128.5 (2C), 127.4, 127.3, 127.1, 127.0 (2C), 121.4, 120.7 (2C), 118.84, 118.82, 113.9 (2C), 113.7, 113.6 (2C), 110.2, 55.2, 55.0, 49.0, 48.6.

MS (ESI): *m*/z = 477 [M + H]^+^

HRMS (TOF ES^+^): *m*/*z* [M + H]^+^ calcd for C_31_H_29_N_2_${\text{O}}_{3}^{+}$: 477.2173; found: 477.2193.

#### 2-(1-Benzyl-1*H*-Indol-3-yl)-*N*-(4-Chlorophenyl)-2-(4-Methoxyphenyl)acetamide (8h)

White solid; yield 132 mg (54%); mp 179–181°C; *R*_*f*_ = 0.30 (EtOAc–hexane, 1:4).

IR (film): 3282, 3061, 3030, 2928, 1658, 1593, 1510, 1493, 1467, 1397, 1299, 1248, 1173, 1093, 1030, 824, 786, 739, 697 cm^−1^.

^1^H NMR (600 MHz, CDCl_3_): δ = 7.65 (s, 1H), 7.44 (d, *J* = 8.0 Hz, 1H), 7.35 (d, *J* = 9.0 Hz, 2H), 7.34 – 7.25 (m, 6H), 7.20 (d, *J* = 8.6 Hz, 2H), 7.19 – 7.16 (m, 1H), 7.10 – 7.06 (m, 3H), 7.01 (s, 1H), 6.87 (d, *J* = 8.6 Hz, 2H), 5.27 (s, 2H), 5.21 (s, 1H), 3.78 (s, 3H).

^13^C NMR (150 MHz, CDCl_3_): δ = 171.0, 159.0, 137.3, 137.1, 136.4, 131.1, 129.7 (2C), 129.4, 129.0 (2C), 128.9 (2C), 128.0, 127.8, 127.2, 126.8 (2C), 122.6, 121.2 (2C), 120.1, 119.3, 114.4 (2C), 113.6, 110.3, 55.4, 51.3, 50.3.

MS (ESI): *m*/z = 481 [M + H]^+^

HRMS (TOF ES^+^): *m*/*z* [M + H]^+^ calcd for C_30_H_25_ClN_2_${\text{O}}_{2}^{+}$: 481.1677; found: 481.1683.

## Data Availability

The raw data supporting the conclusions of this manuscript will be made available by the authors, without undue reservation, to any qualified researcher.

## Author Contributions

NG and HN were responsible for designing and performing the experiments. NG, HN, AG and AV discussed the evolution of the project and revised the manuscript together. LV and EV directed the project and wrote the publication.

### Conflict of Interest Statement

The authors declare that the research was conducted in the absence of any commercial or financial relationships that could be construed as a potential conflict of interest.
