# Supplementary material for: Three-Component Reaction of 3-Arylidene-3H-Indolium Salts, Isocyanides, and Alcohols
Source: Front Chem. 2019 May 16;7:345. doi: 10.3389/fchem.2019.00345 (PMC6532454; doi:10.3389/fchem.2019.00345)
Supplement: Supplementary file 1 [file Data_Sheet_1.docx]

Supplementary Material

**Three-component reaction of 3-arylidene-3*H*-indolium salts, isocyanides and alcohols**

**Nikita E. Golantsov^1^, Hung M. Nguyen^1^, Alexandra S. Golubenkova^1^, Alexey V. Varlamov^1^, Erik V. Van der Eycken^1,2*^, Leonid G. Voskressensky^1*^**

^1^Department of Organic Chemistry, Faculty of Science, Peoples’ Friendship University of Russia (RUDN University), Moscow, Russia

^2^Laboratory for Organic & Microwave-Assisted Chemistry (LOMAC), Department of Chemistry, University of Leuven (KU Leuven), Leuven, Belgium

*** Correspondence:**Erik V. Van der Eycken
[erik.vandereycken@kuleuven.be](mailto:erik.vandereycken@kuleuven.be)

Leonid G. Voskressensky
[lvoskressensky@sci.pfu.edu.ru](mailto:lvoskressensky@sci.pfu.edu.ru)

1. ^1^H and ^13^C NMR spectra of 3-arylidene-3*H*-indolium salts **1a-f** 2-7

2. ^1^H and ^13^C NMR spectra of imidates **4a-r** 8-25

3. ^1^H and ^13^C NMR spectra of ether **5** 26

4**.** ^1^H and ^13^C NMR spectra of amides **6a-h** 27-34

1. ^1^H and ^13^C NMR spectra of 3-arylidene-3*H*-indolium salts **1a-f**


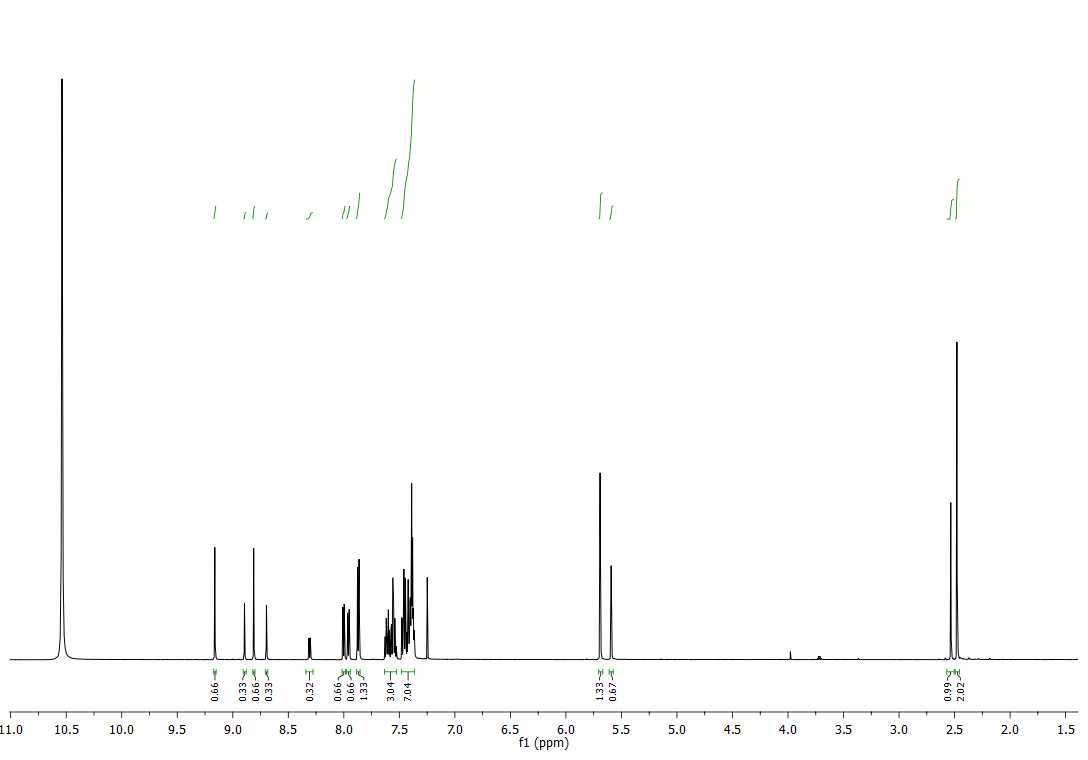


**Supplementary Figure 1.** ^1^H NMR spectrum of compound **1a** (600 MHz, CDCl_3_+TFA).


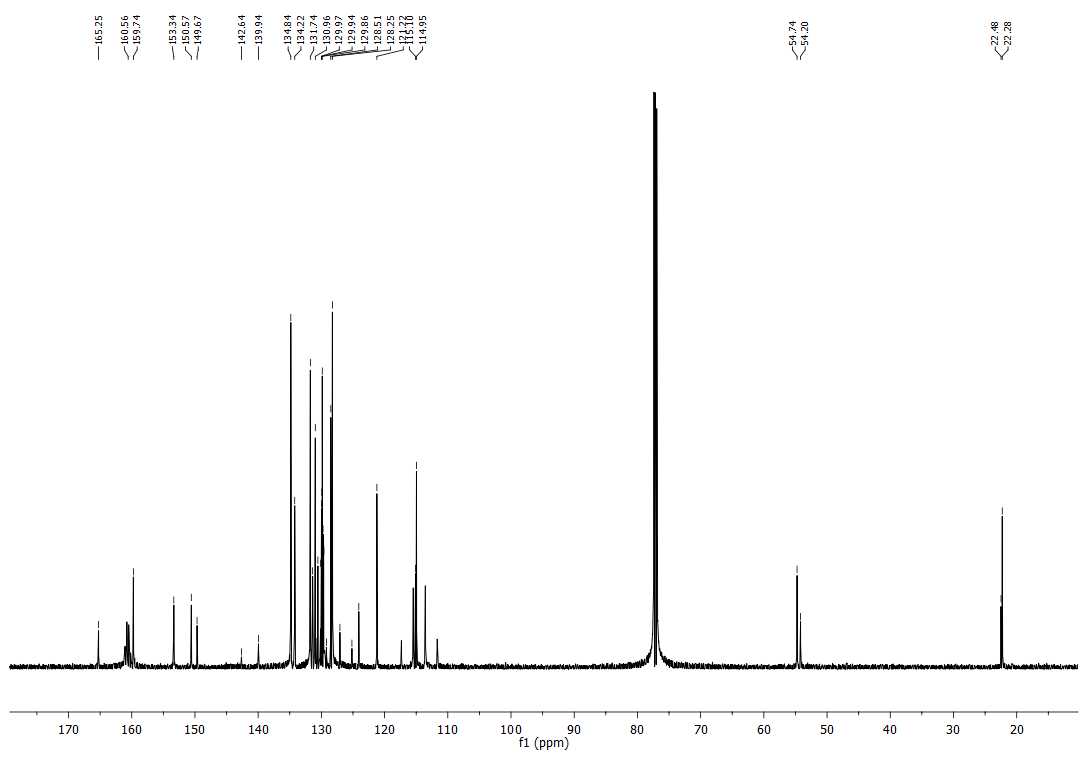


**Supplementary Figure 2.** ^13^C NMR spectrum of compound **1a** (150 MHz, CDCl_3_+TFA).


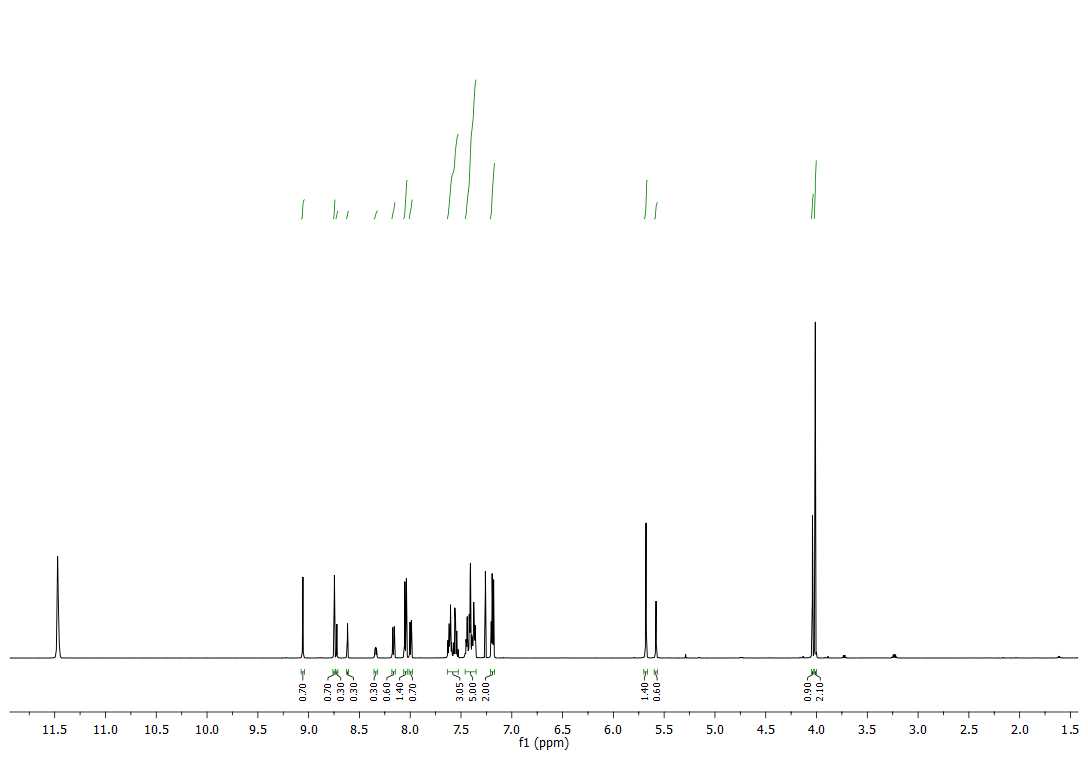


**Supplementary Figure 3.** ^1^H NMR spectrum of compound **1b** (600 MHz, CDCl_3_+TFA).

**
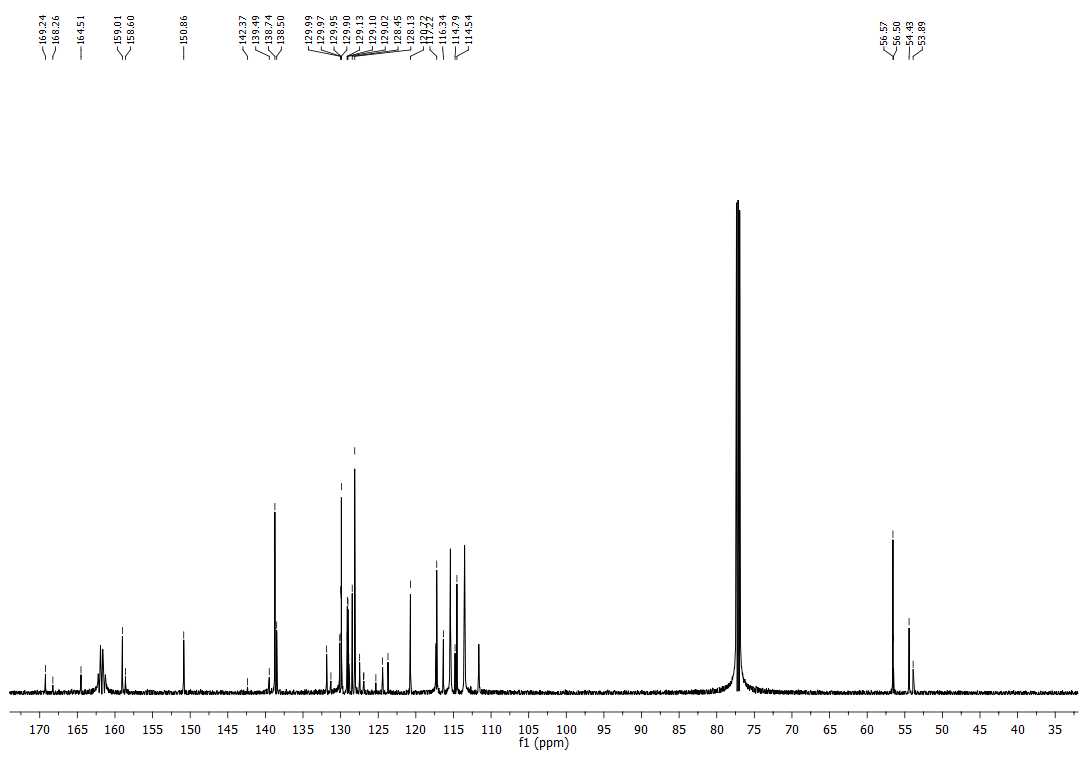
**

**Supplementary Figure 4.** ^13^C NMR spectrum of compound **1b** (150 MHz, CDCl_3_+TFA).

**
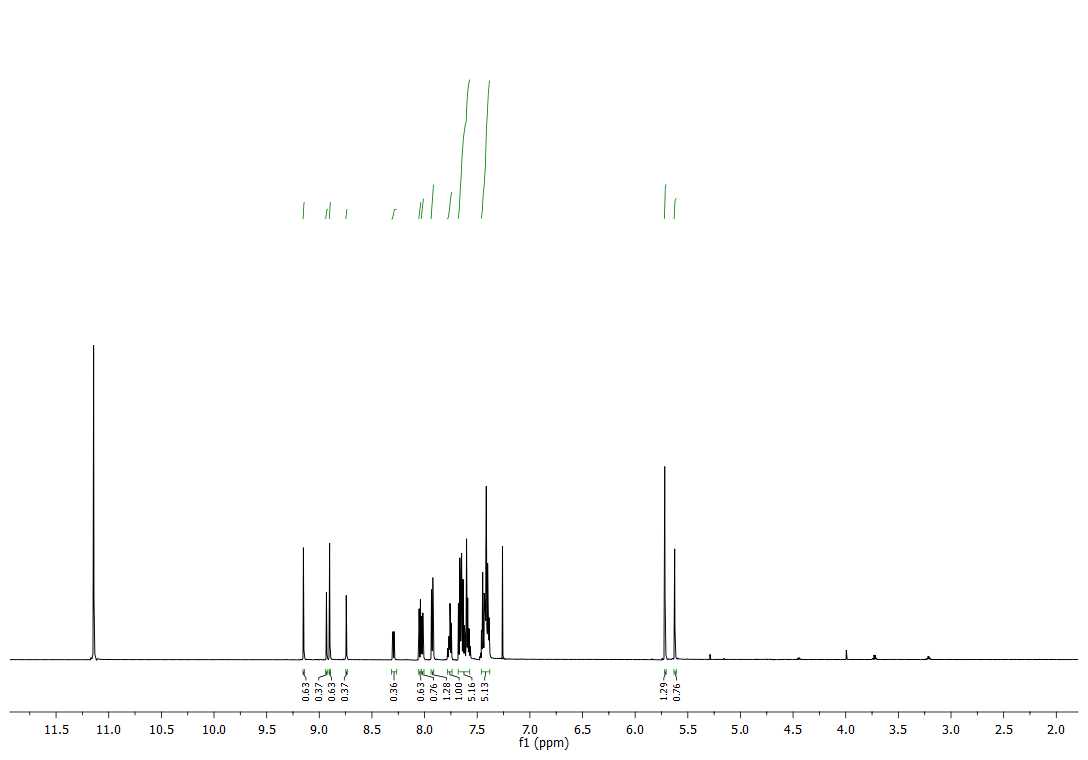
**

**Supplementary Figure 5.** ^1^H NMR spectrum of compound **1c** (600 MHz, CDCl_3_+TFA).

**
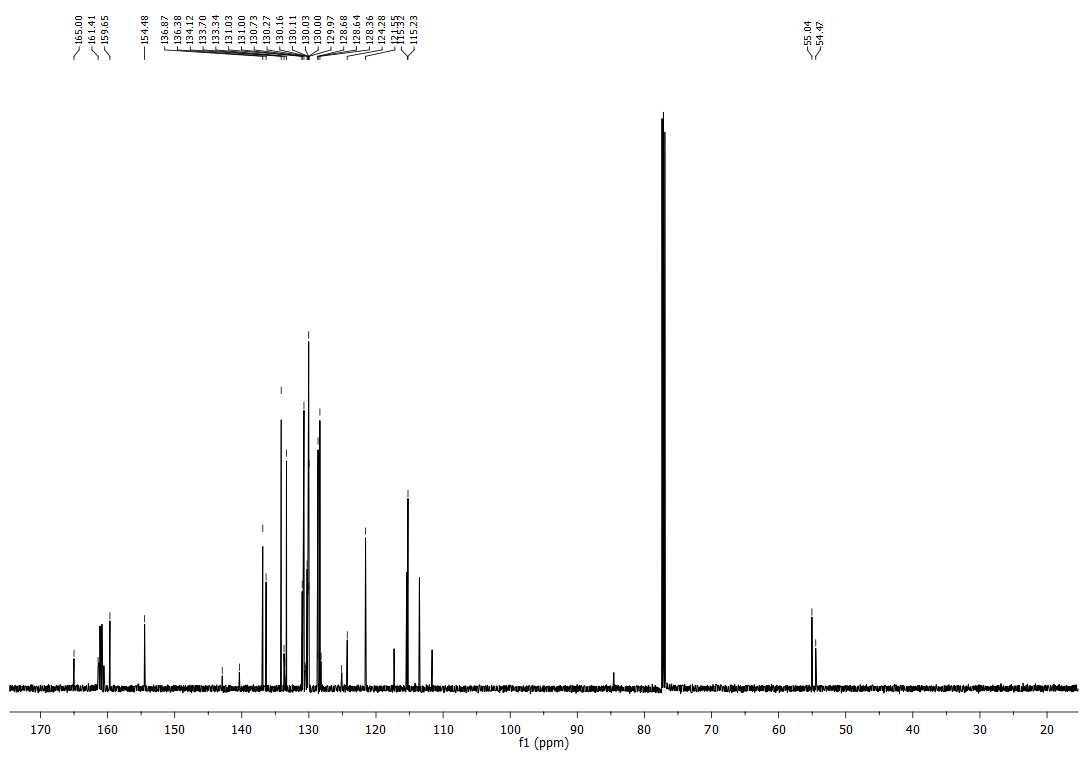
**

**Supplementary Figure 6.** ^13^C NMR spectrum of compound **1c** (150 MHz, CDCl_3_+TFA).

**
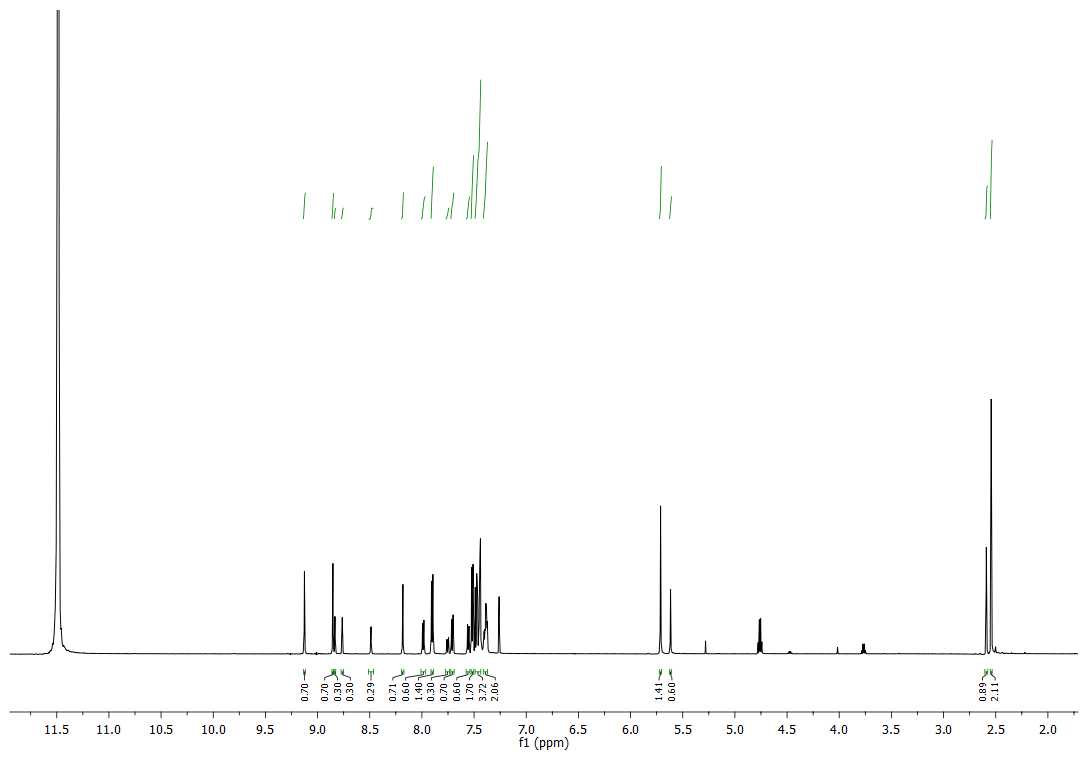
**

**Supplementary Figure 7.** ^1^H NMR spectrum of compound **1d** (600 MHz, CDCl_3_+TFA).


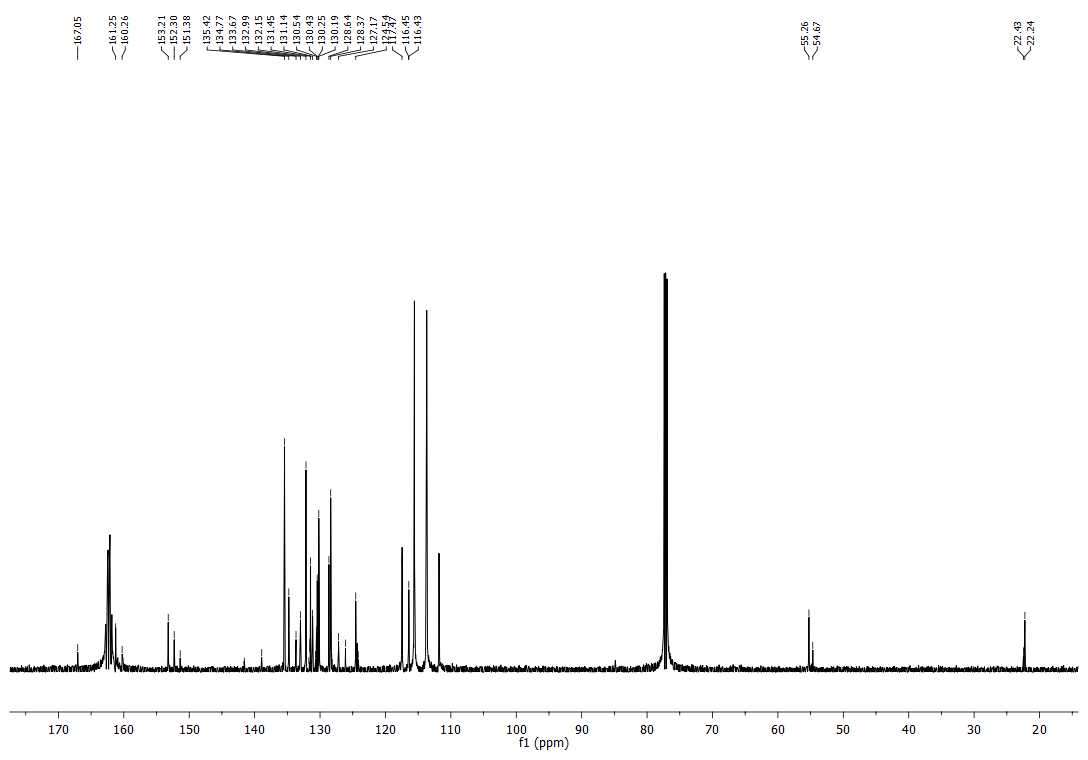


**Supplementary Figure 8.** ^13^C NMR spectrum of compound **1d** (150 MHz, CDCl_3_+TFA).

**
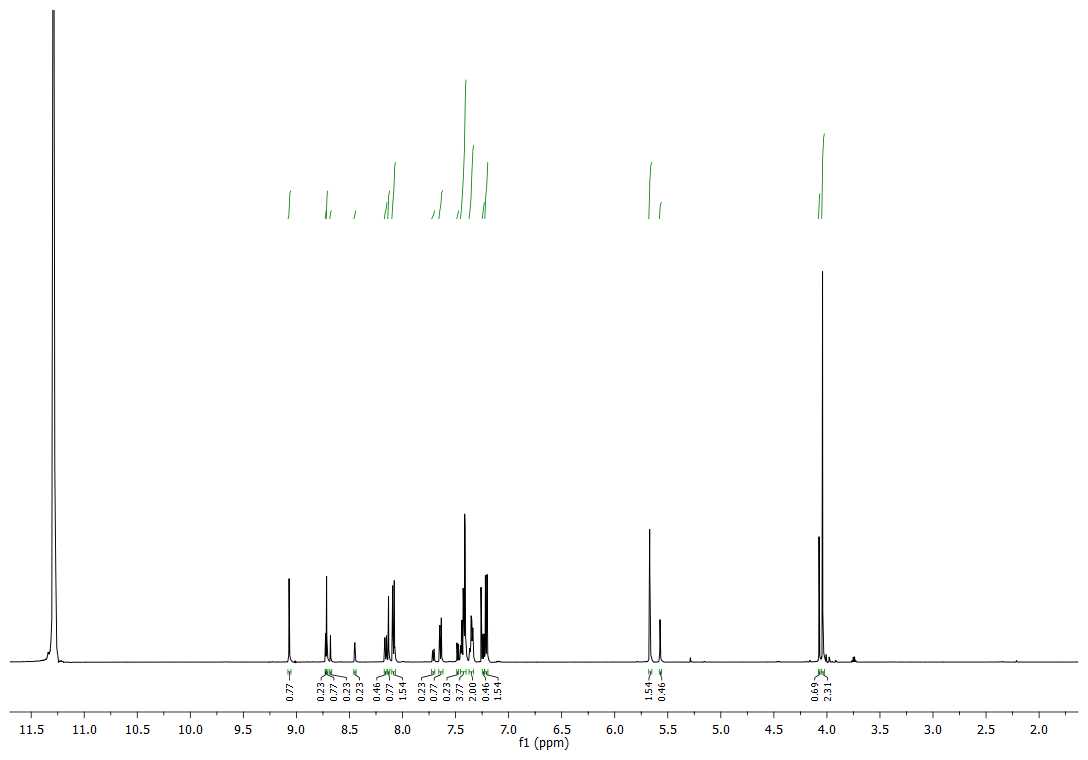
**

**Supplementary Figure 9.** ^1^H NMR spectrum of compound **1e** (600 MHz, CDCl_3_+TFA).


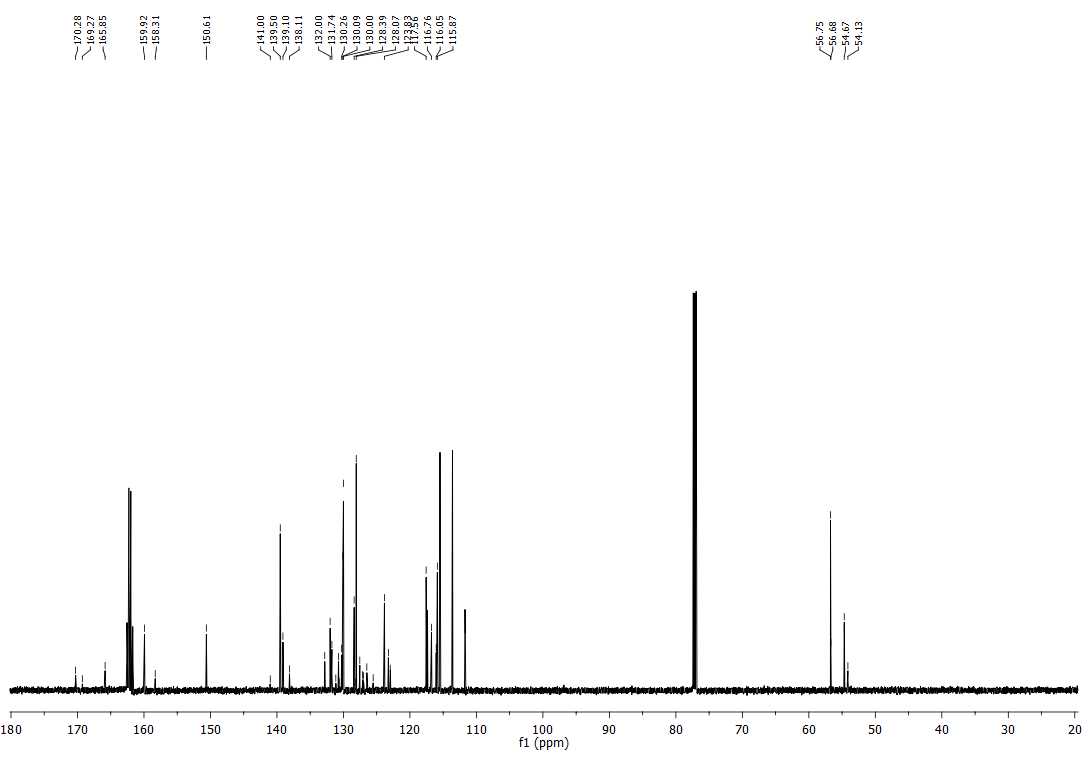


**Supplementary Figure 10.** ^13^C NMR spectrum of compound **1e** (150 MHz, CDCl_3_+TFA).

**
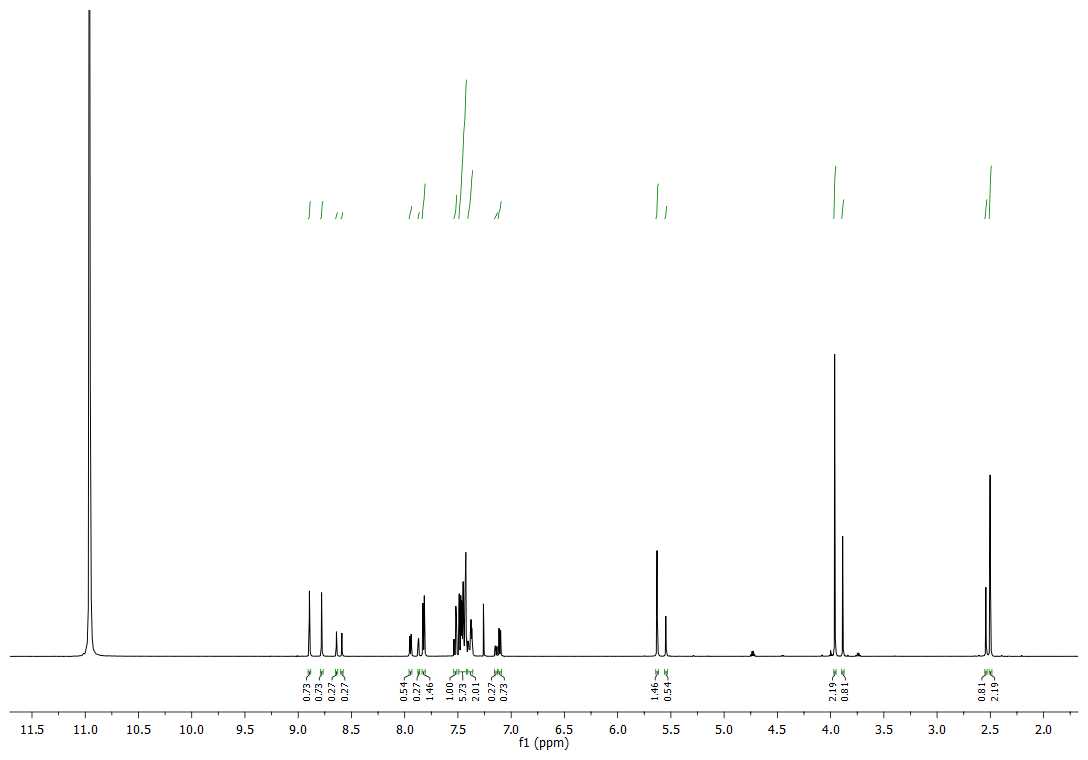
**

**Supplementary Figure 11.** ^1^H NMR spectrum of compound **1f** (600 MHz, CDCl_3_+TFA).


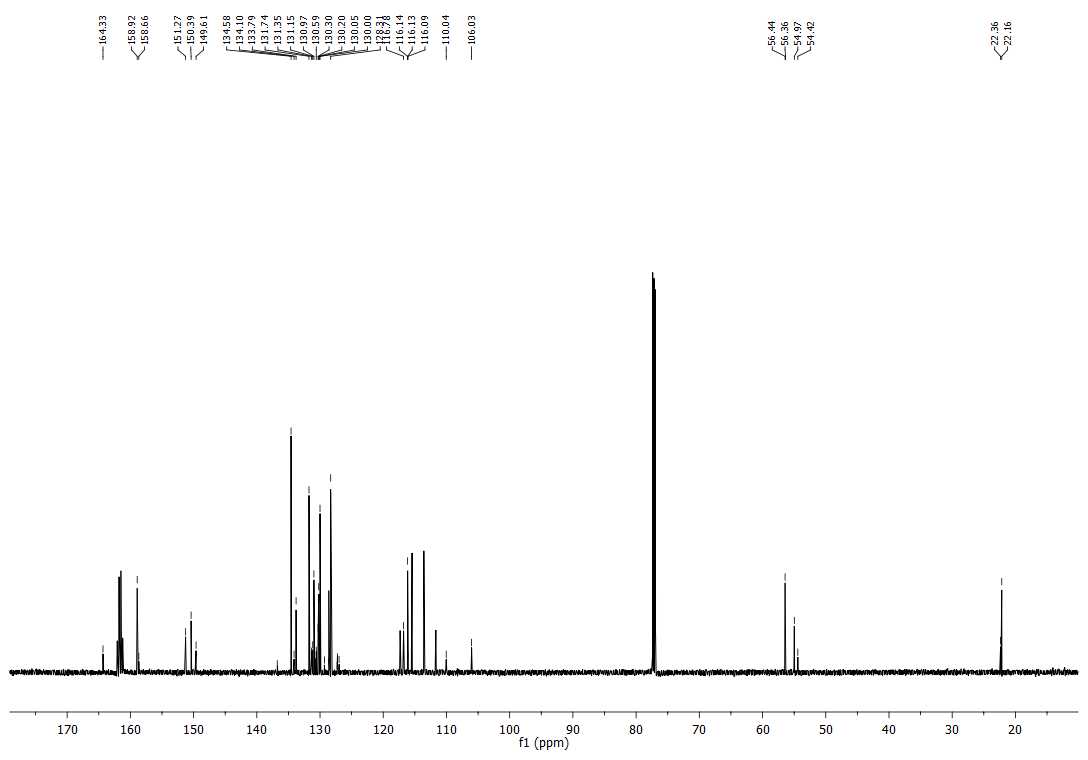


**Supplementary Figure 12.** ^13^C NMR spectrum of compound **1f** (150 MHz, CDCl_3_+TFA).

2. ^1^H and ^13^C NMR spectra of imidates **4a-r**

**
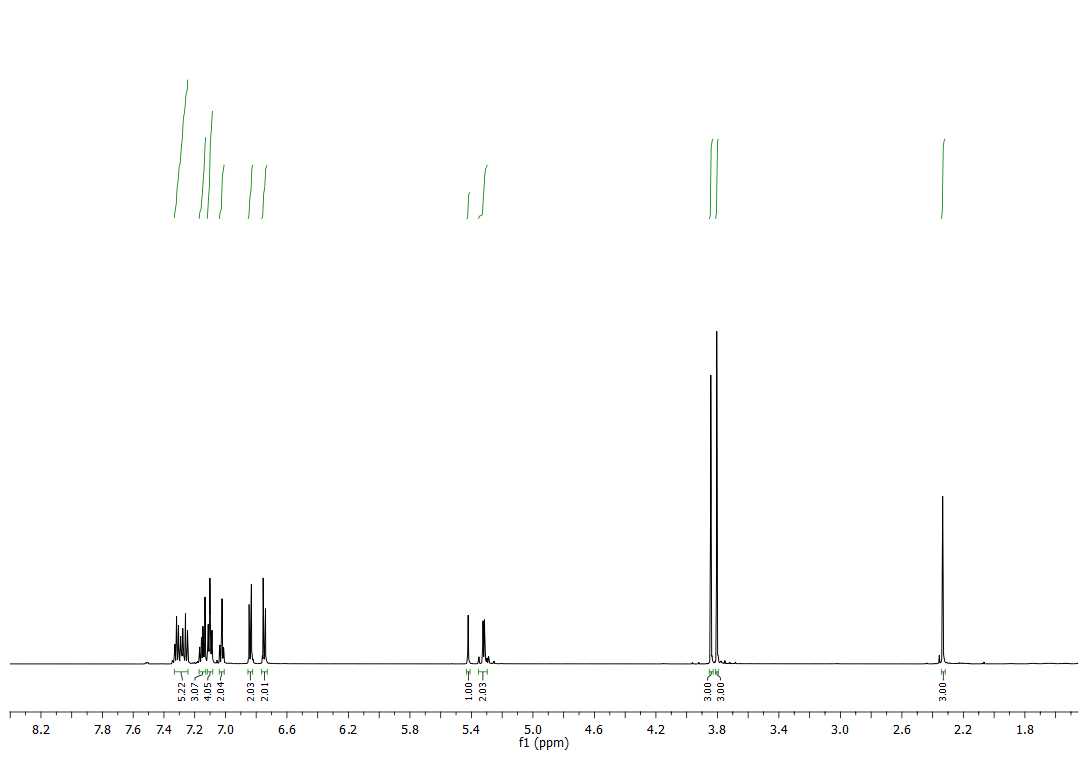
**

**Supplementary Figure 13.** ^1^H NMR spectrum of compound **4a** (600 MHz, CDCl_3_).


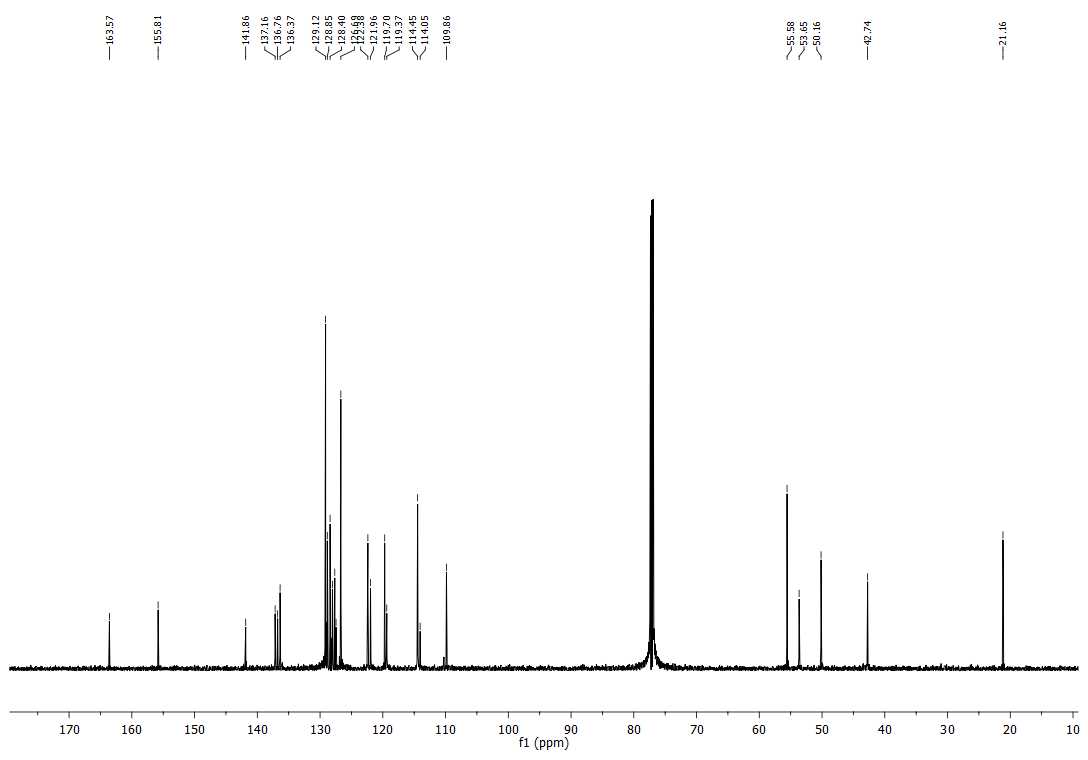


**Supplementary Figure 14.** ^13^C NMR spectrum of compound **4a** (150 MHz, CDCl_3_).

**
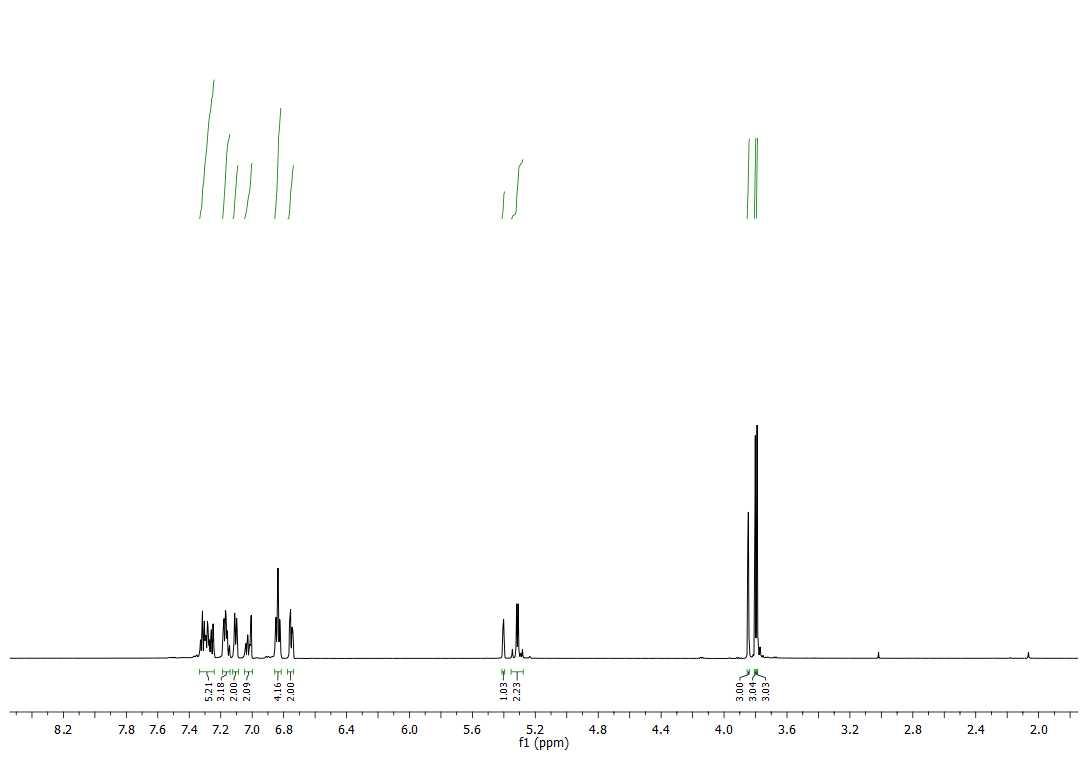
**

**Supplementary Figure 15.** ^1^H NMR spectrum of compound **4b** (600 MHz, CDCl_3_).


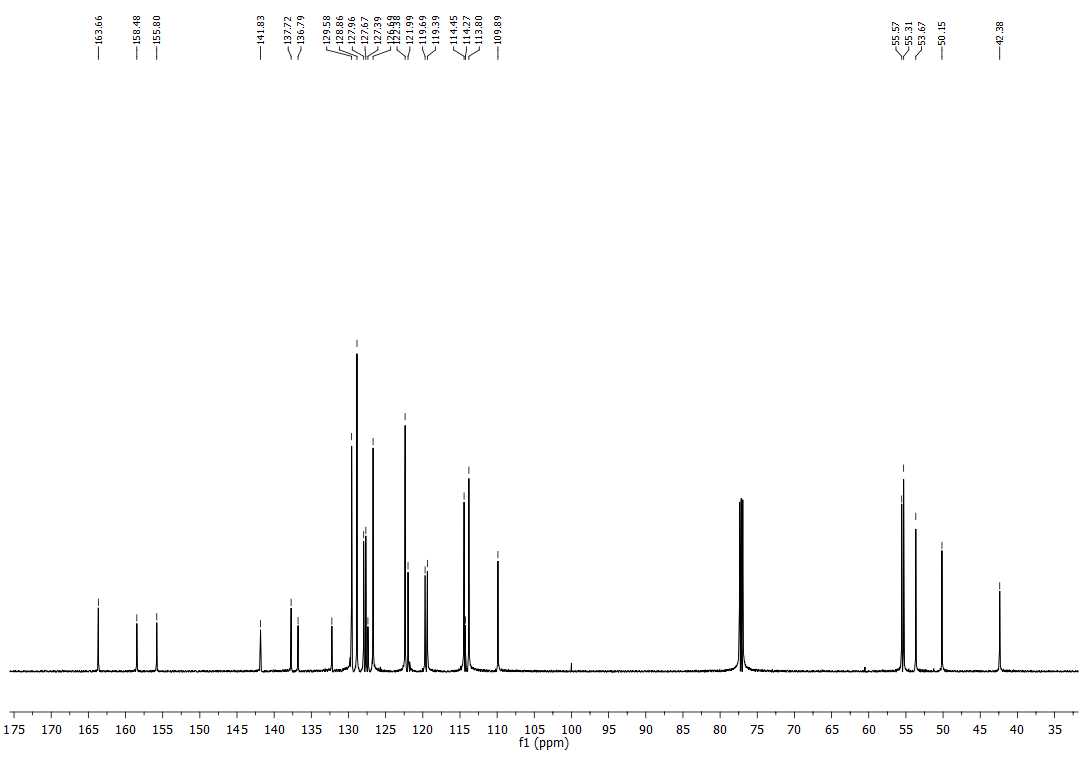


**Supplementary Figure 16.** ^13^C NMR spectrum of compound **4b** (150 MHz, CDCl_3_).

**
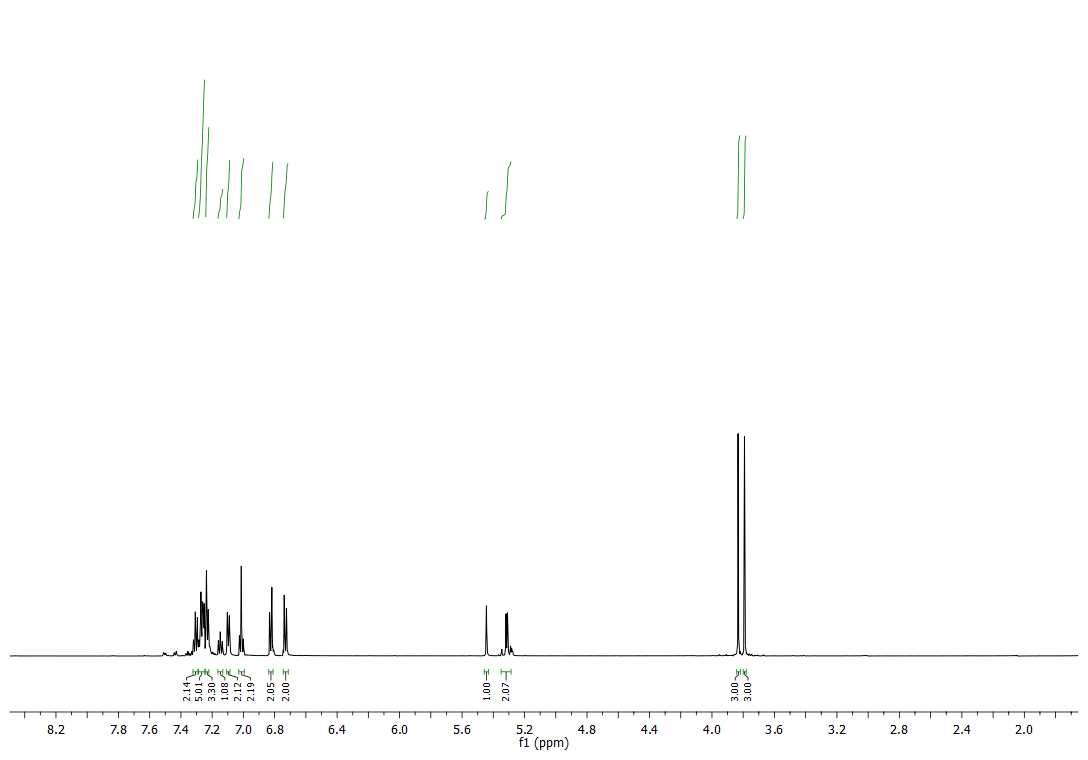
**

**Supplementary Figure 17.** ^1^H NMR spectrum of compound **4c** (600 MHz, CDCl_3_).


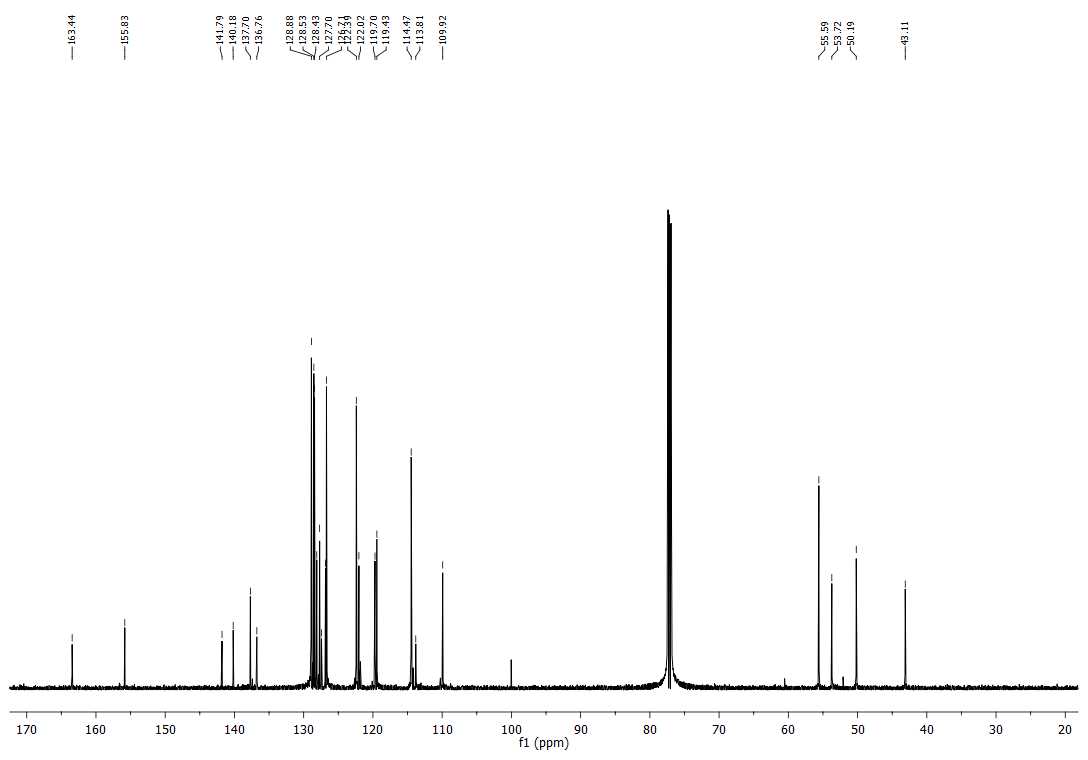


**Supplementary Figure 18.** ^13^C NMR spectrum of compound **4c** (150 MHz, CDCl_3_).

**
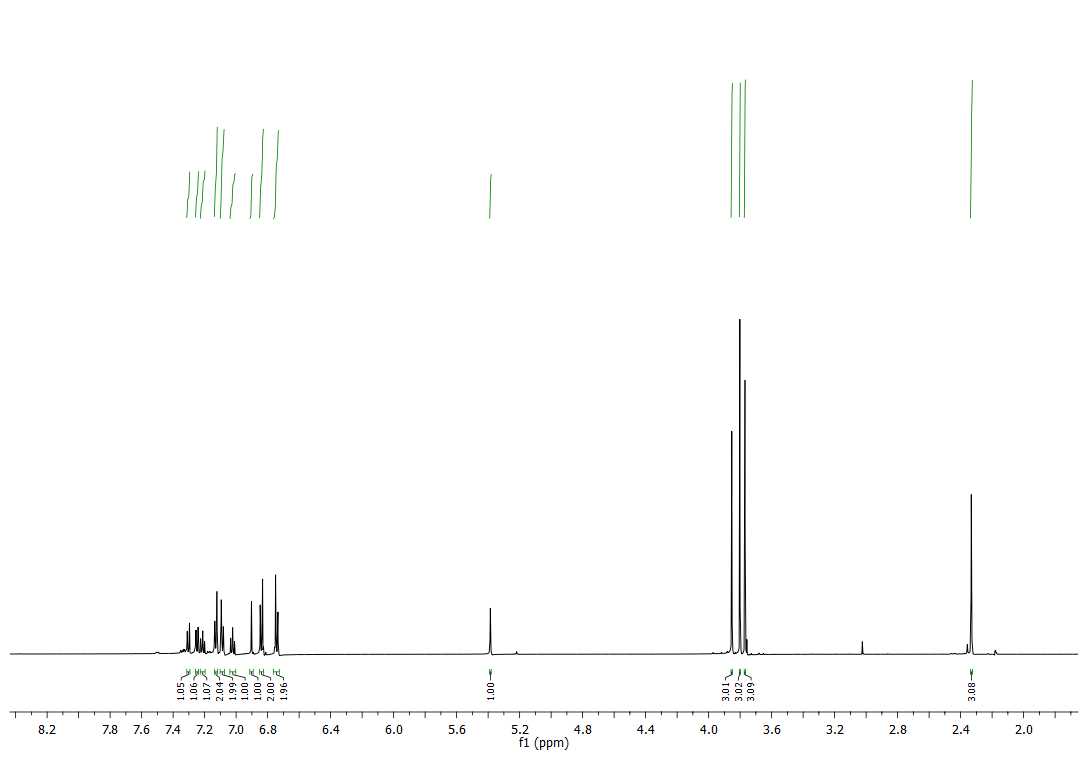
**

**Supplementary Figure 19.** ^1^H NMR spectrum of compound **4d** (600 MHz, CDCl_3_).


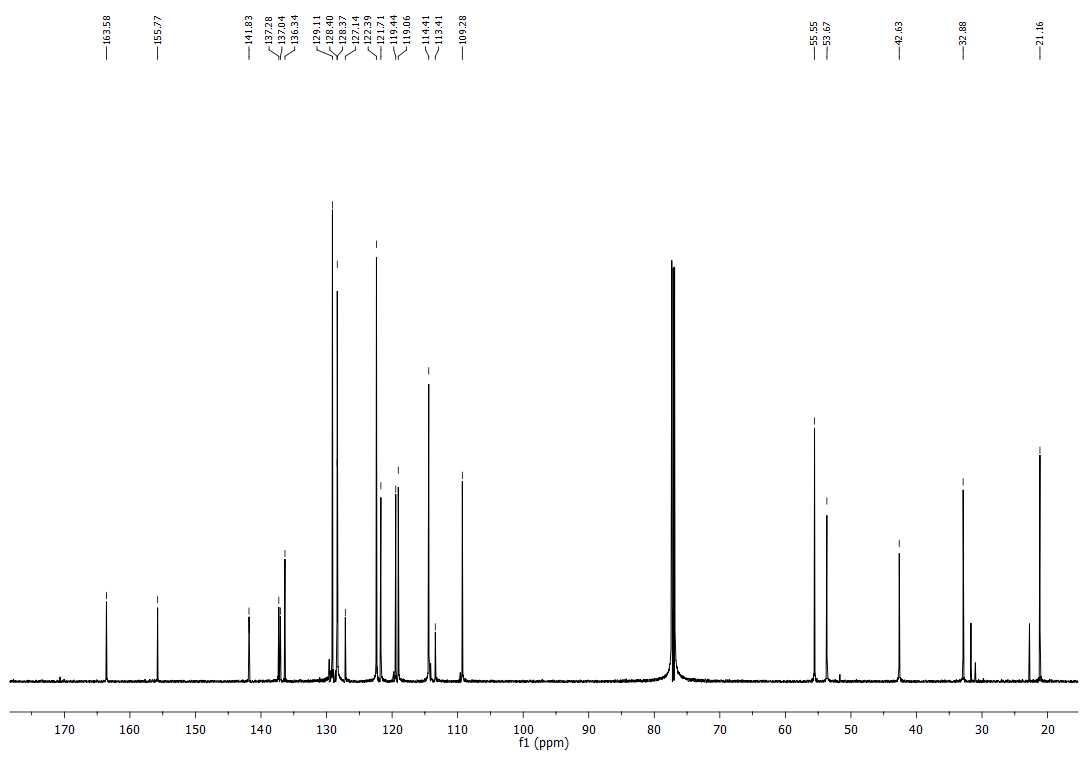


**Supplementary Figure 20.** ^13^C NMR spectrum of compound **4d** (150 MHz, CDCl_3_).

**
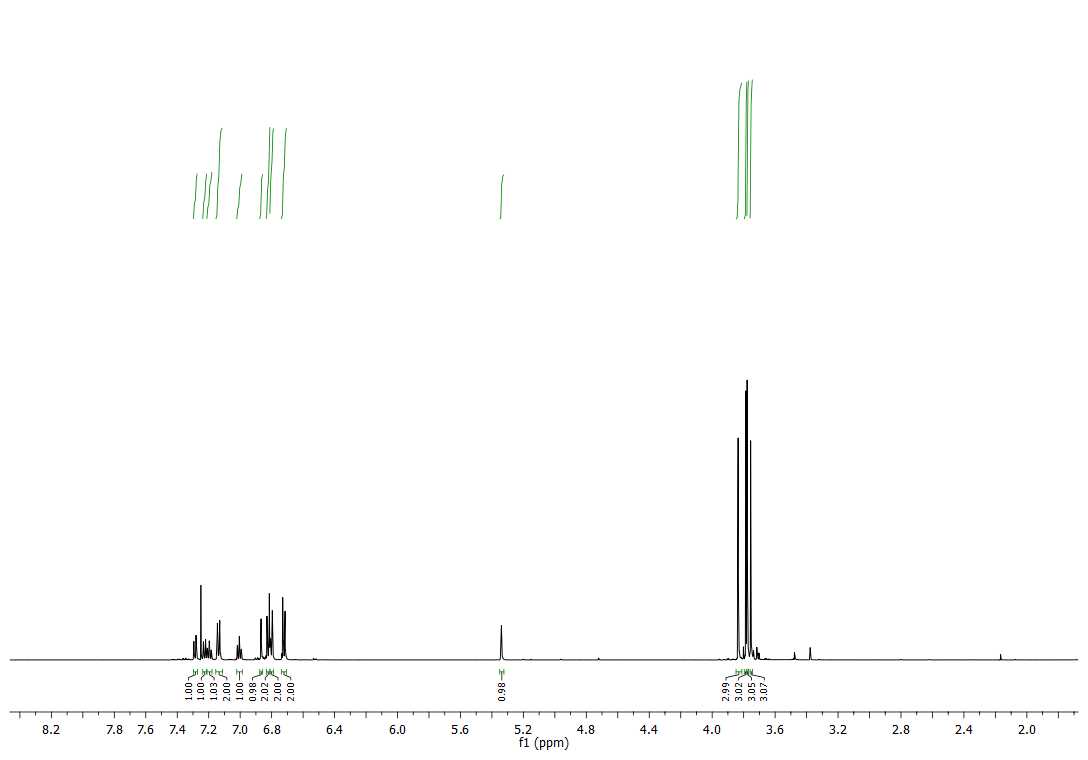
**

**Supplementary Figure 21.** ^1^H NMR spectrum of compound **4e** (600 MHz, CDCl_3_).


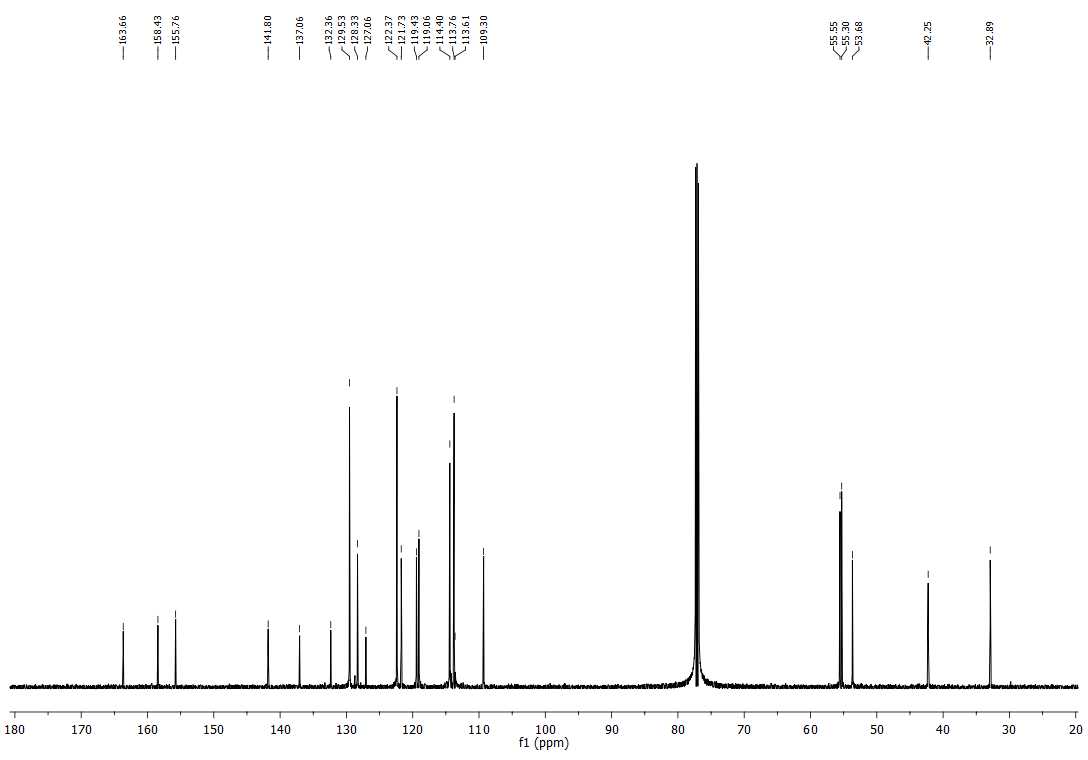


**Supplementary Figure 22.** ^13^C NMR spectrum of compound **4e** (150 MHz, CDCl_3_).

**
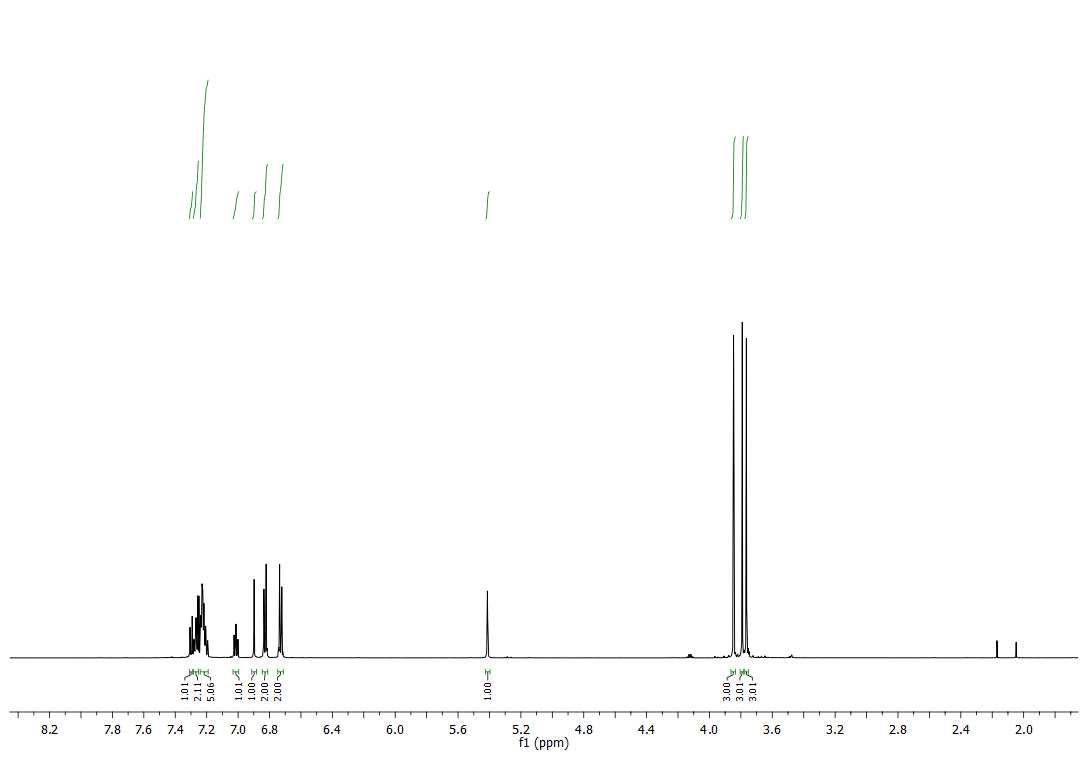
**

**Supplementary Figure 23.** ^1^H NMR spectrum of compound **4f** (600 MHz, CDCl_3_).


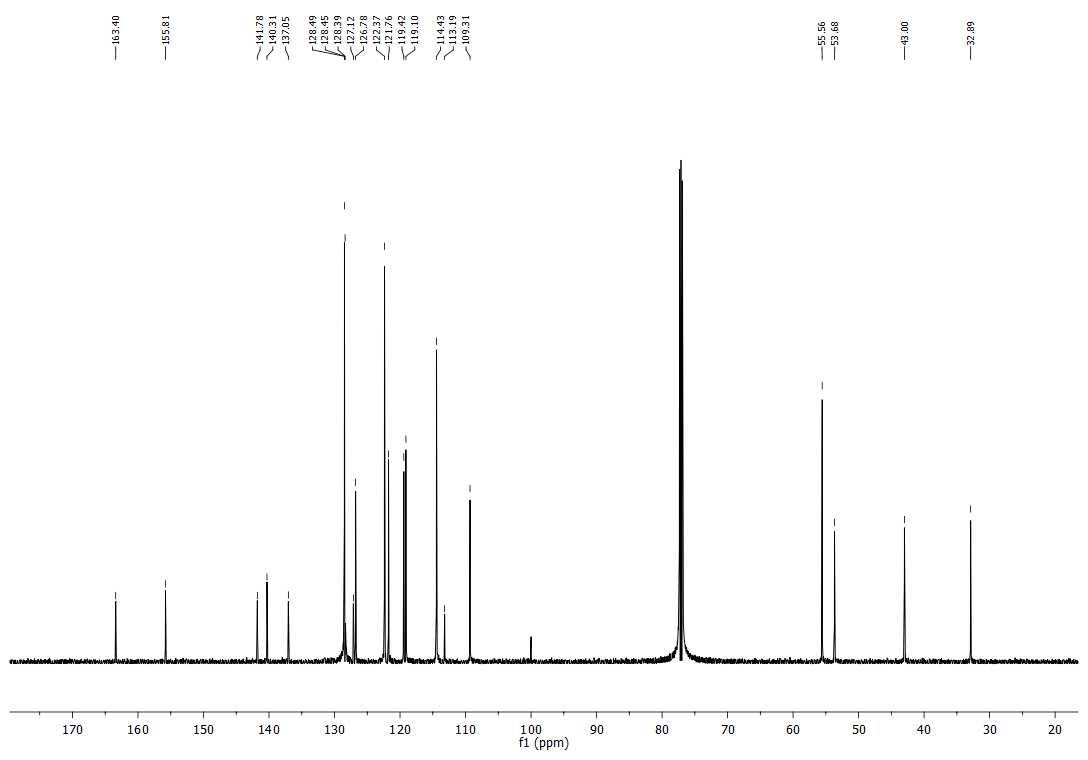


**Supplementary Figure 24.** ^13^C NMR spectrum of compound **4f** (150 MHz, CDCl_3_).

**
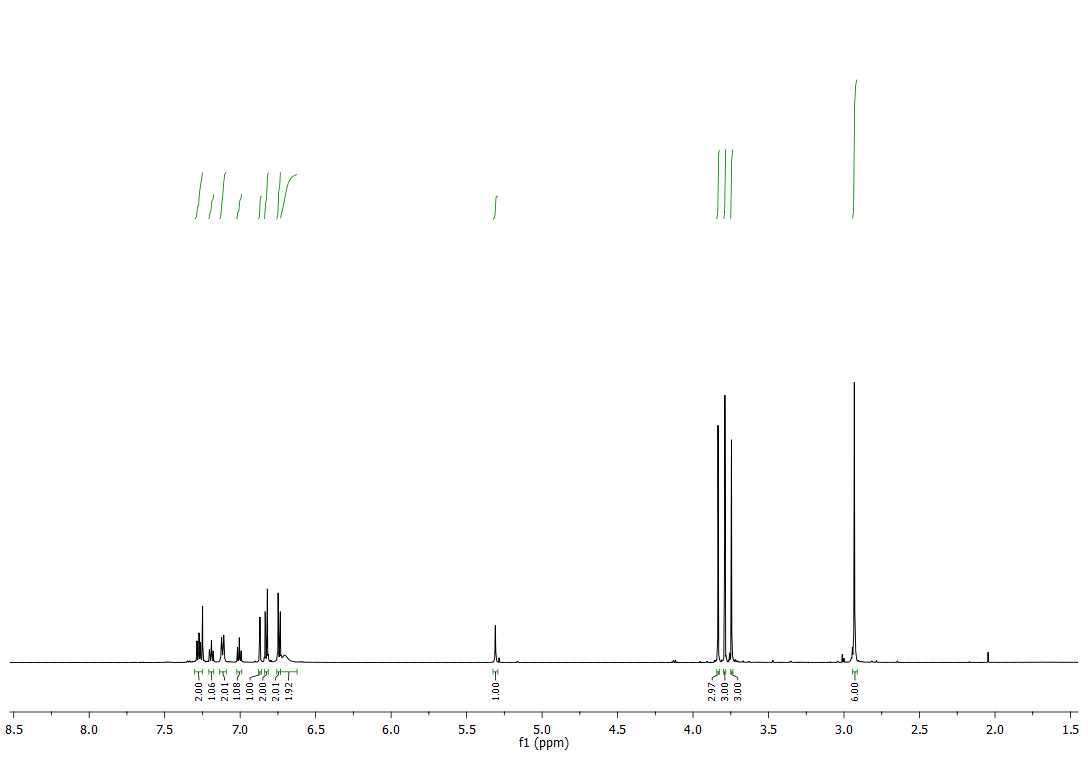
**

**Supplementary Figure 25.** ^1^H NMR spectrum of compound **4g** (600 MHz, CDCl_3_).


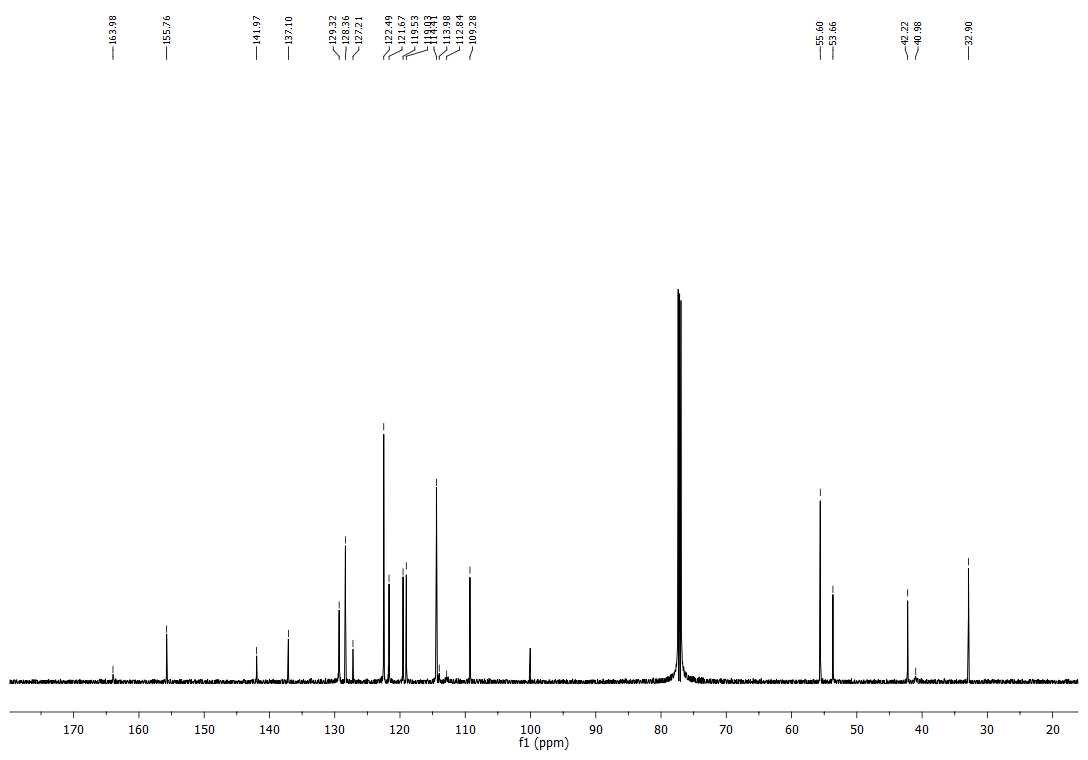


**Supplementary Figure 26.** ^13^C NMR spectrum of compound **4g** (150 MHz, CDCl_3_).

**
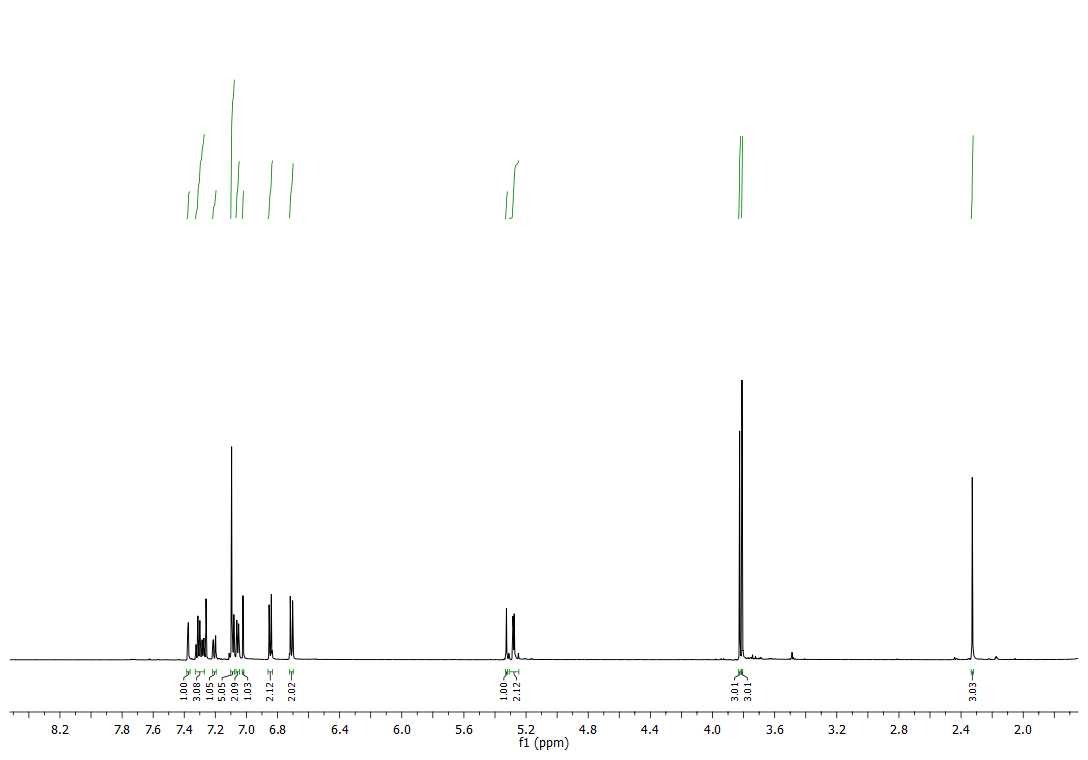
**

**Supplementary Figure 27.** ^1^H NMR spectrum of compound **4h** (600 MHz, CDCl_3_).


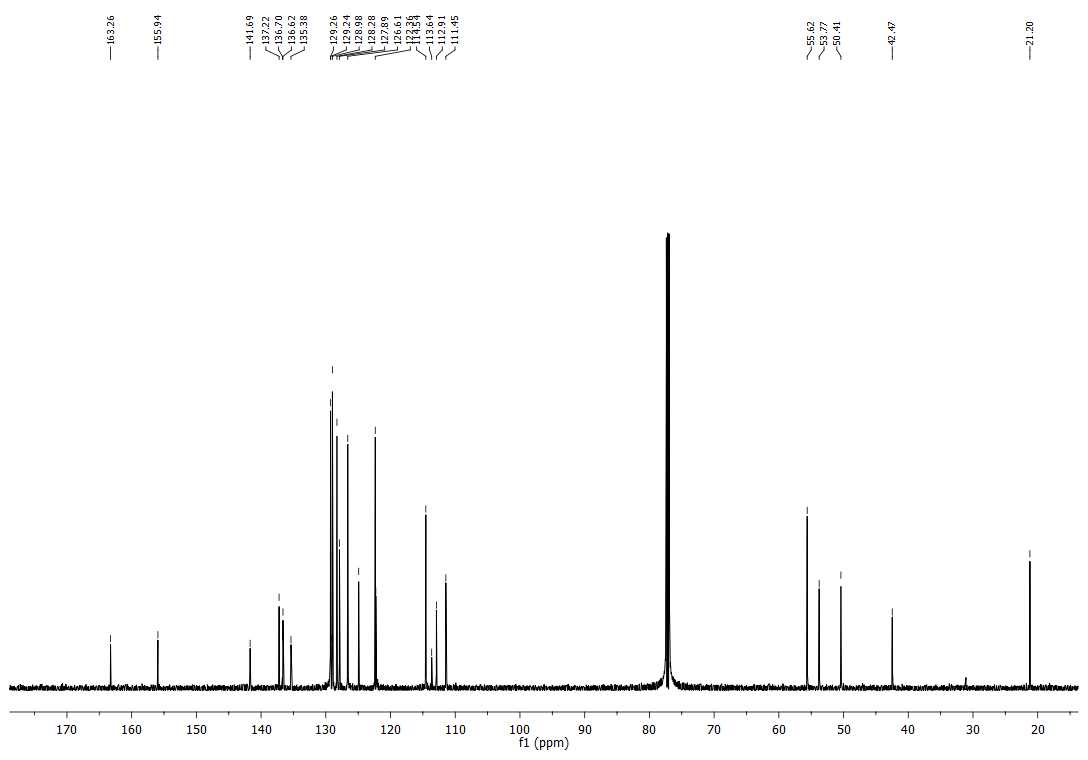


**Supplementary Figure 28.** ^13^C NMR spectrum of compound **4h** (150 MHz, CDCl_3_).

**
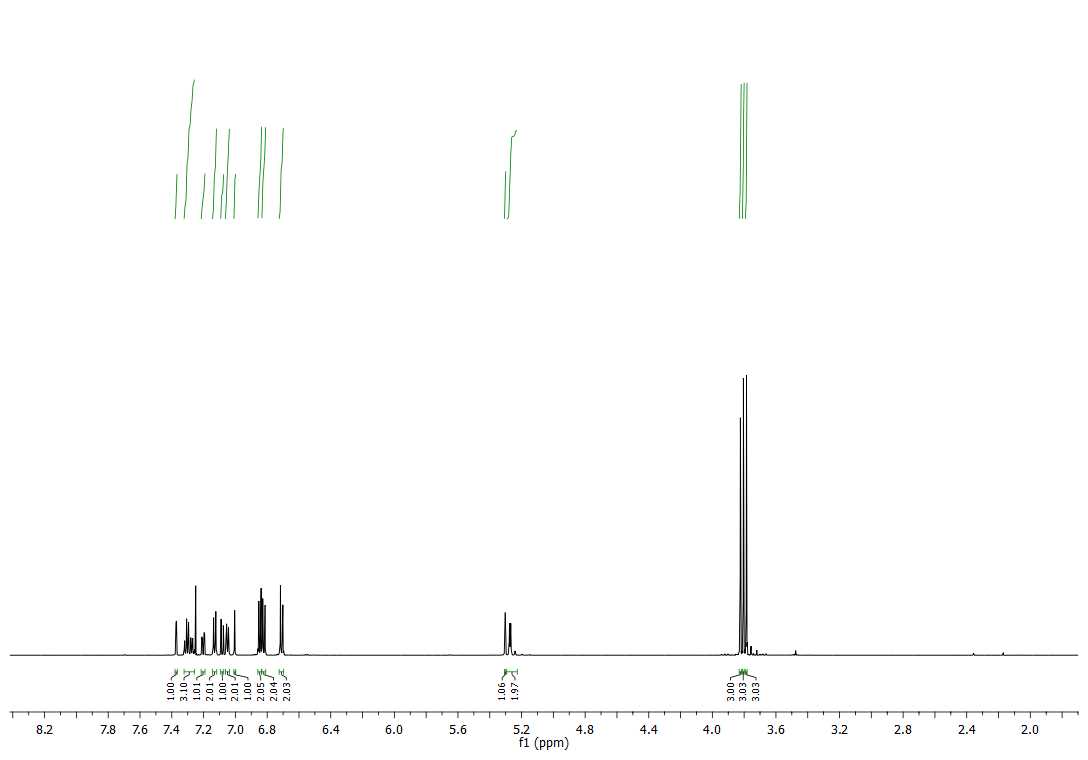
**

**Supplementary Figure 29.** ^1^H NMR spectrum of compound **4i** (600 MHz, CDCl_3_).


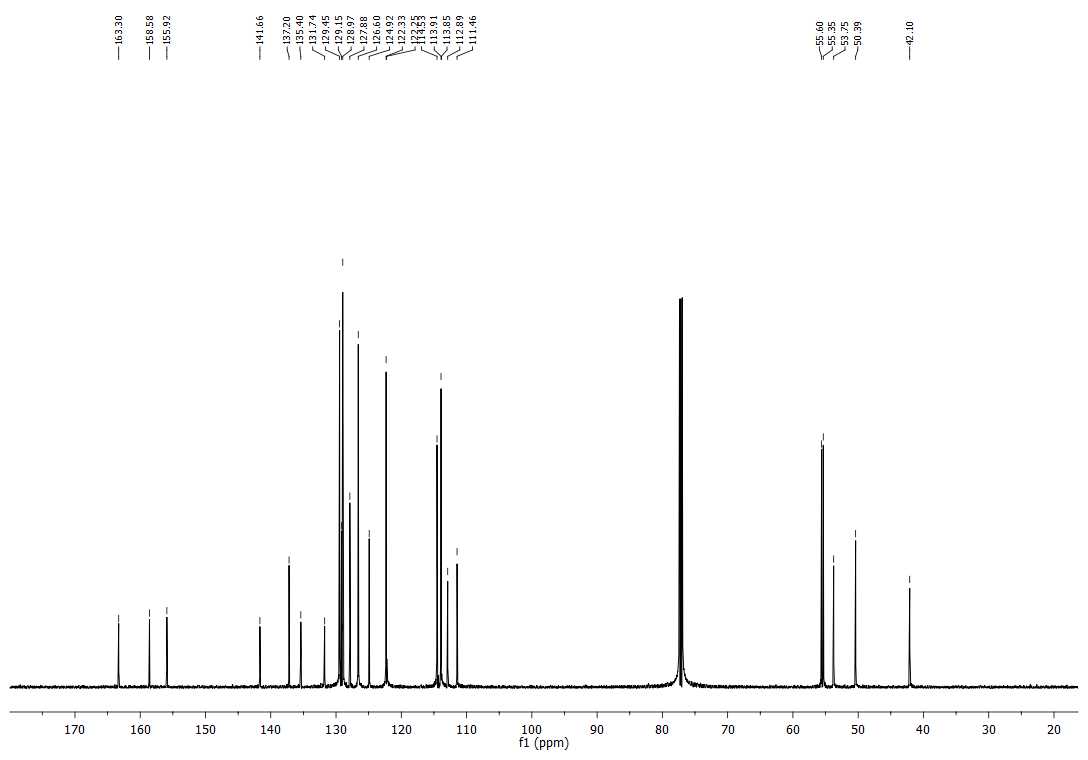


**Supplementary Figure 30.** ^13^C NMR spectrum of compound **4i** (150 MHz, CDCl_3_).

**
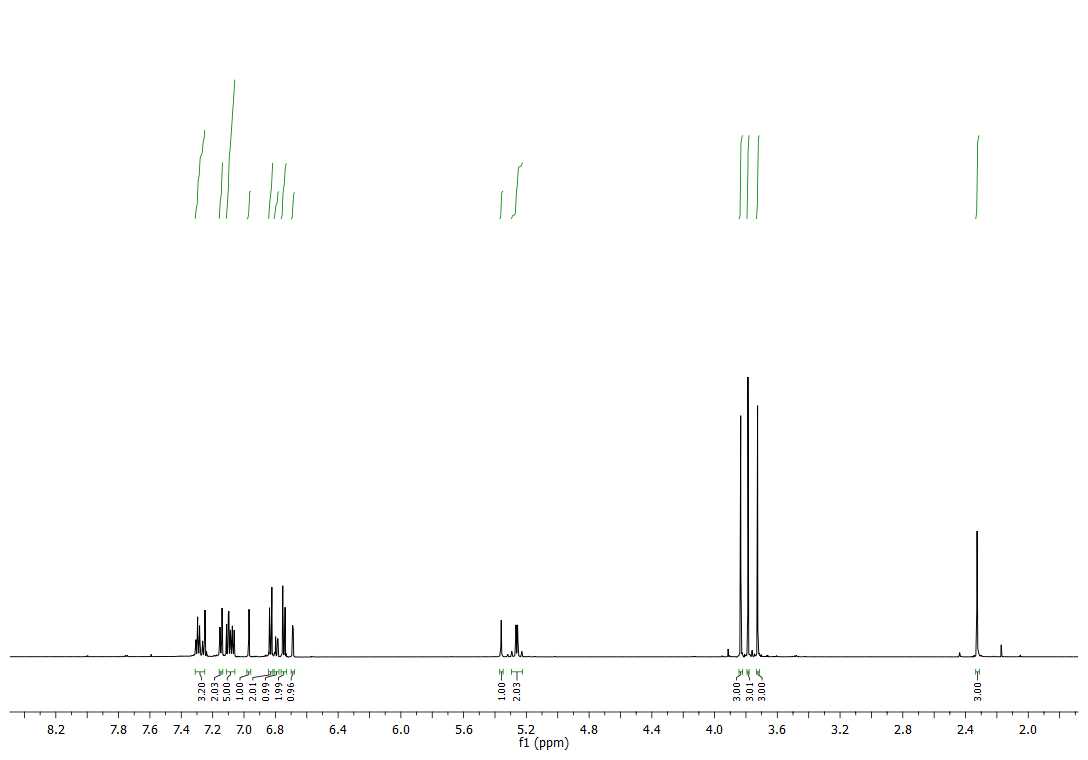
**

**Supplementary Figure 31.** ^1^H NMR spectrum of compound **4j** (600 MHz, CDCl_3_).


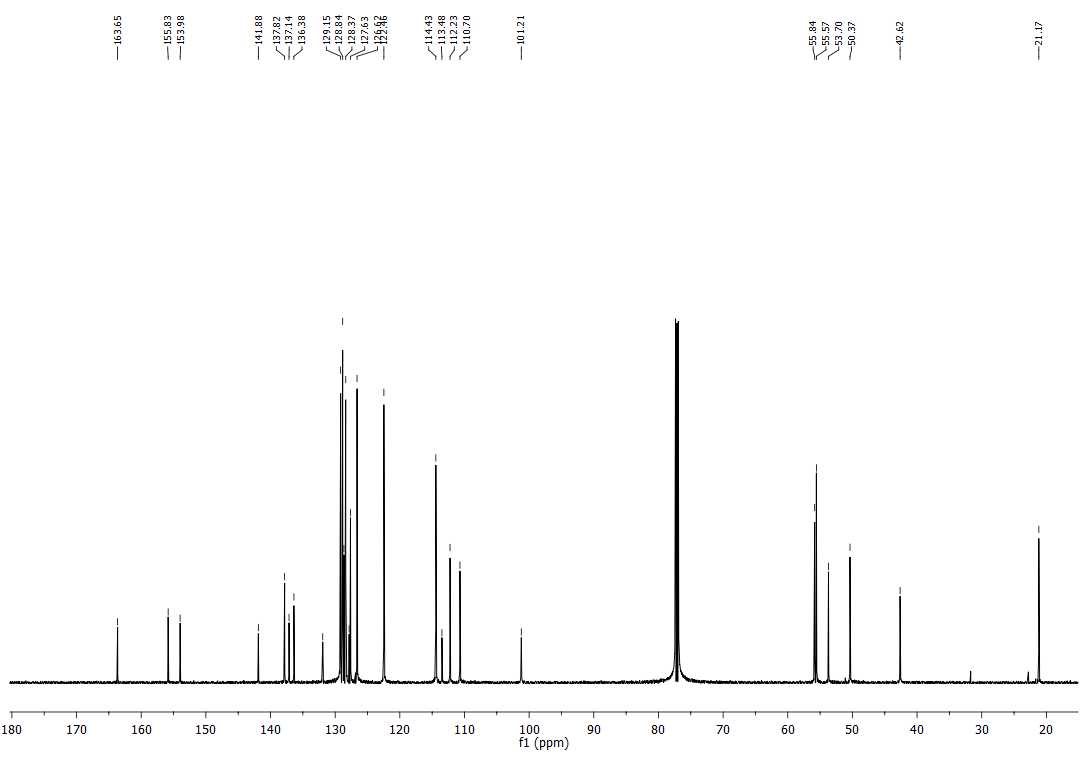


**Supplementary Figure 32.** ^13^C NMR spectrum of compound **4j** (150 MHz, CDCl_3_).

**
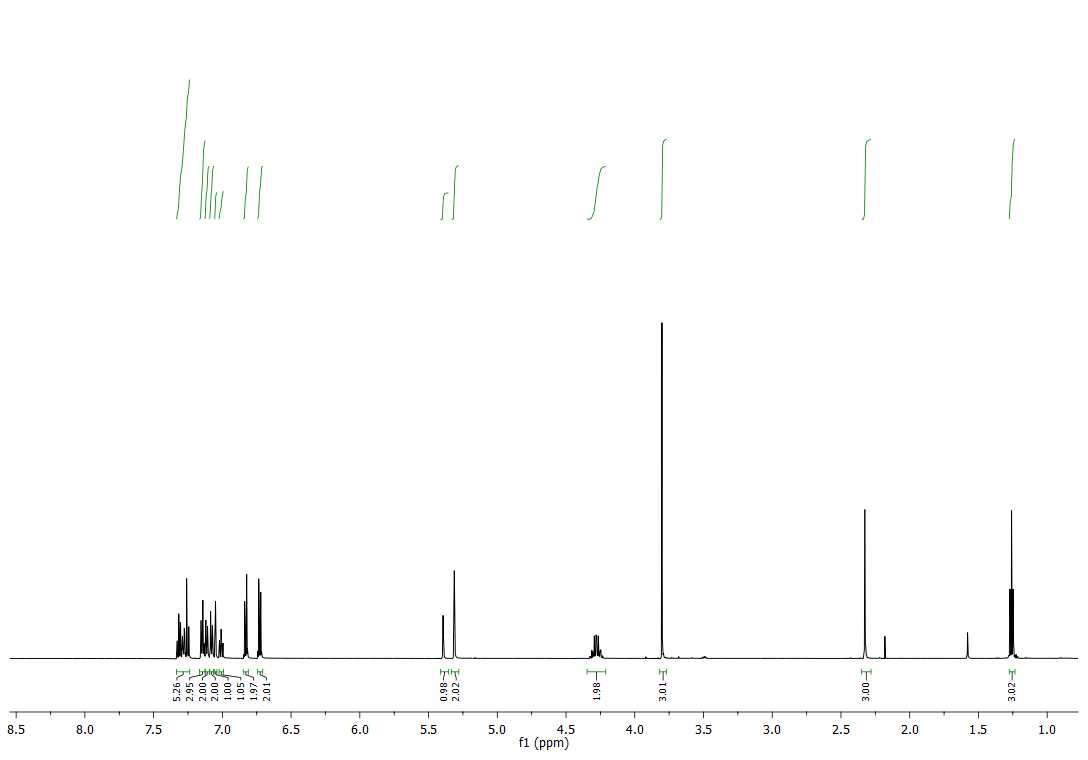
**

**Supplementary Figure 33.** ^1^H NMR spectrum of compound **4k** (600 MHz, CDCl_3_).


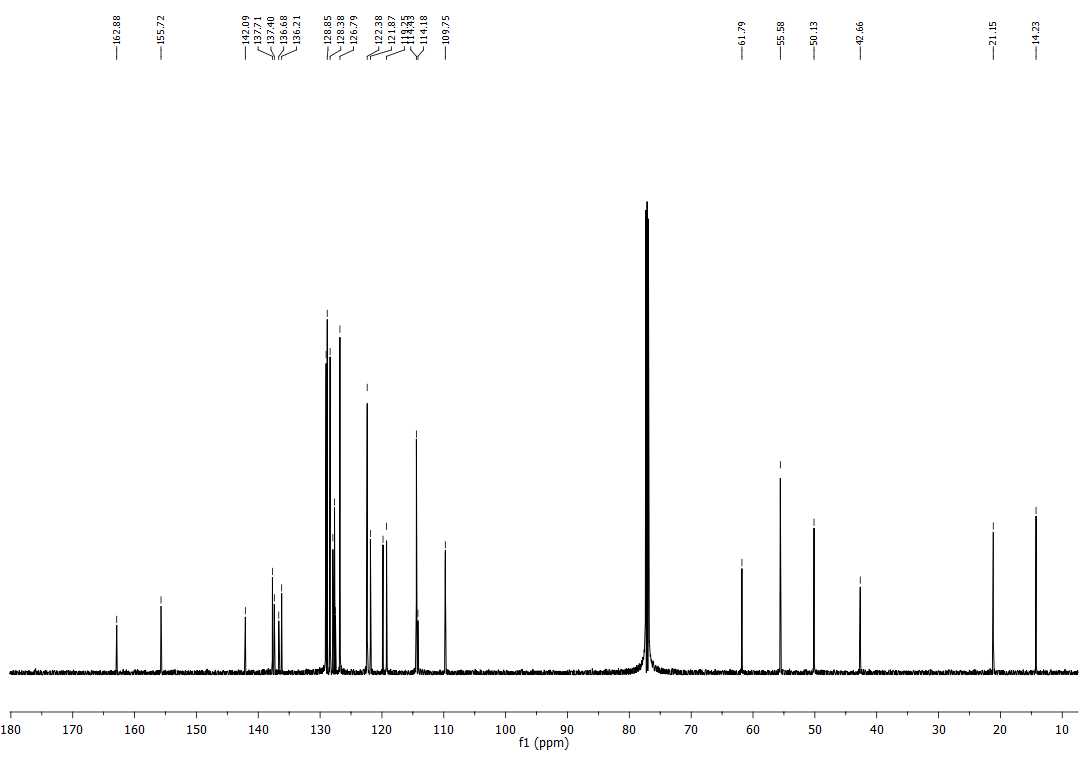


**Supplementary Figure 34.** ^13^C NMR spectrum of compound **4k** (150 MHz, CDCl_3_).

**
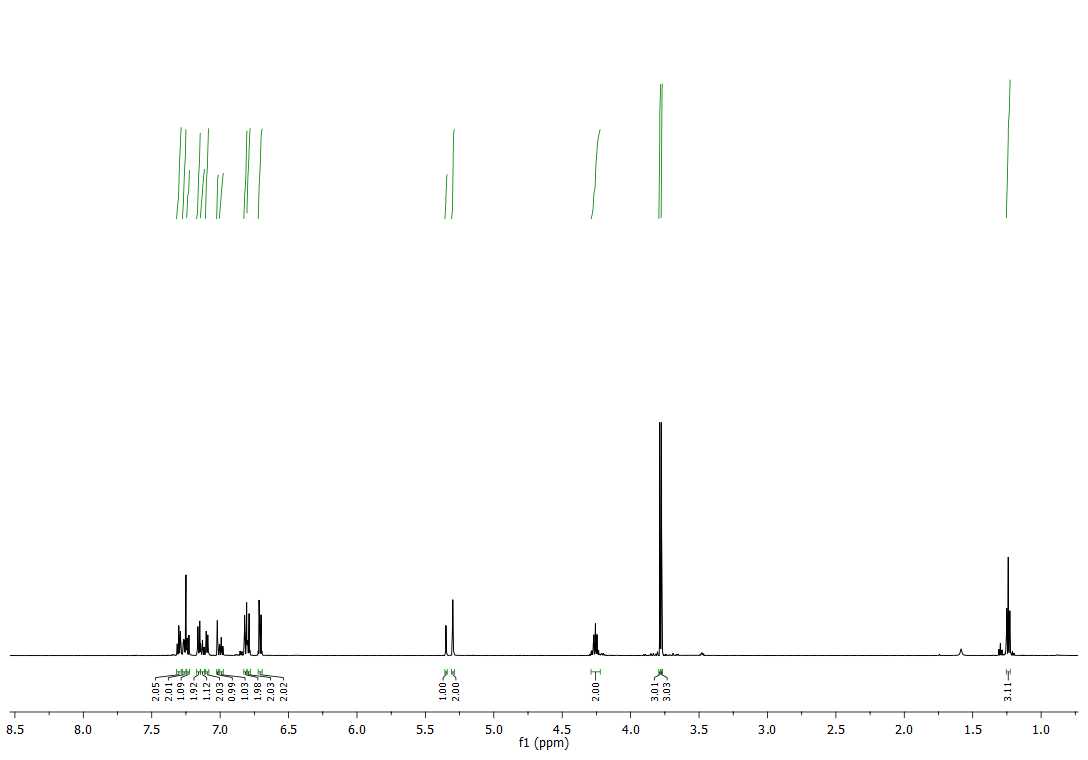
**

**Supplementary Figure 35.** ^1^H NMR spectrum of compound **4l** (600 MHz, CDCl_3_).


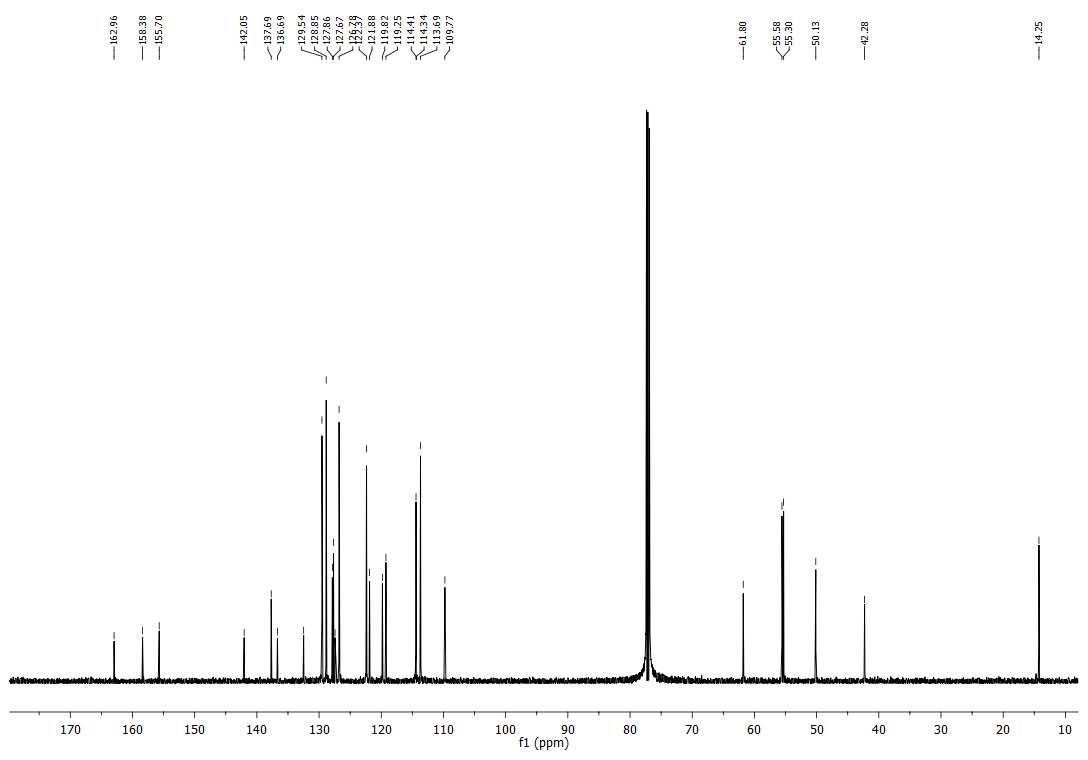


**Supplementary Figure 36.** ^13^C NMR spectrum of compound **4l** (150 MHz, CDCl_3_).

**
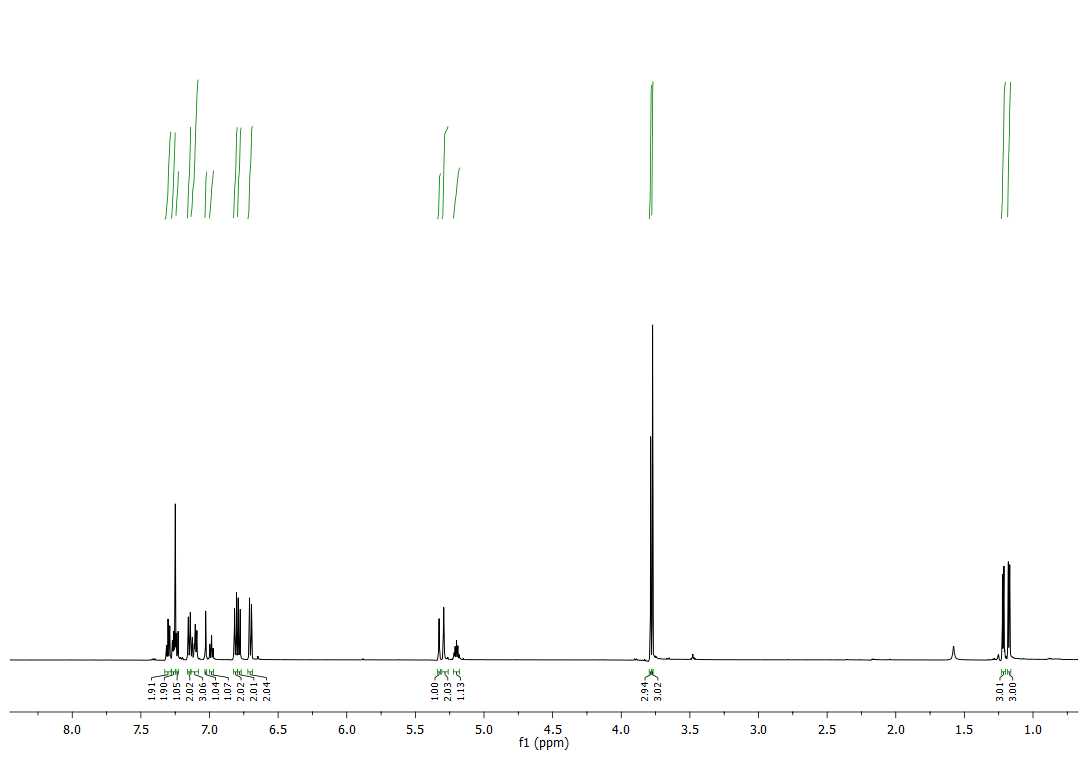
**

**Supplementary Figure 37.** ^1^H NMR spectrum of compound **4m** (600 MHz, CDCl_3_).


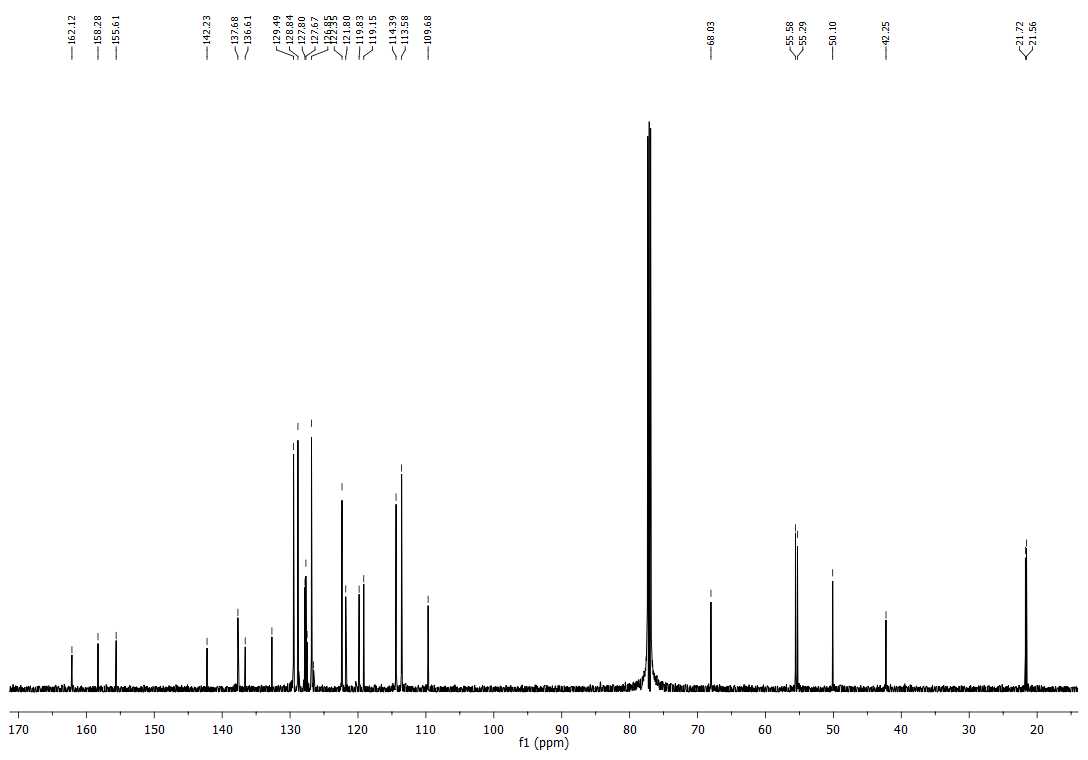


**Supplementary Figure 38.** ^13^C NMR spectrum of compound **4m** (150 MHz, CDCl_3_).

**
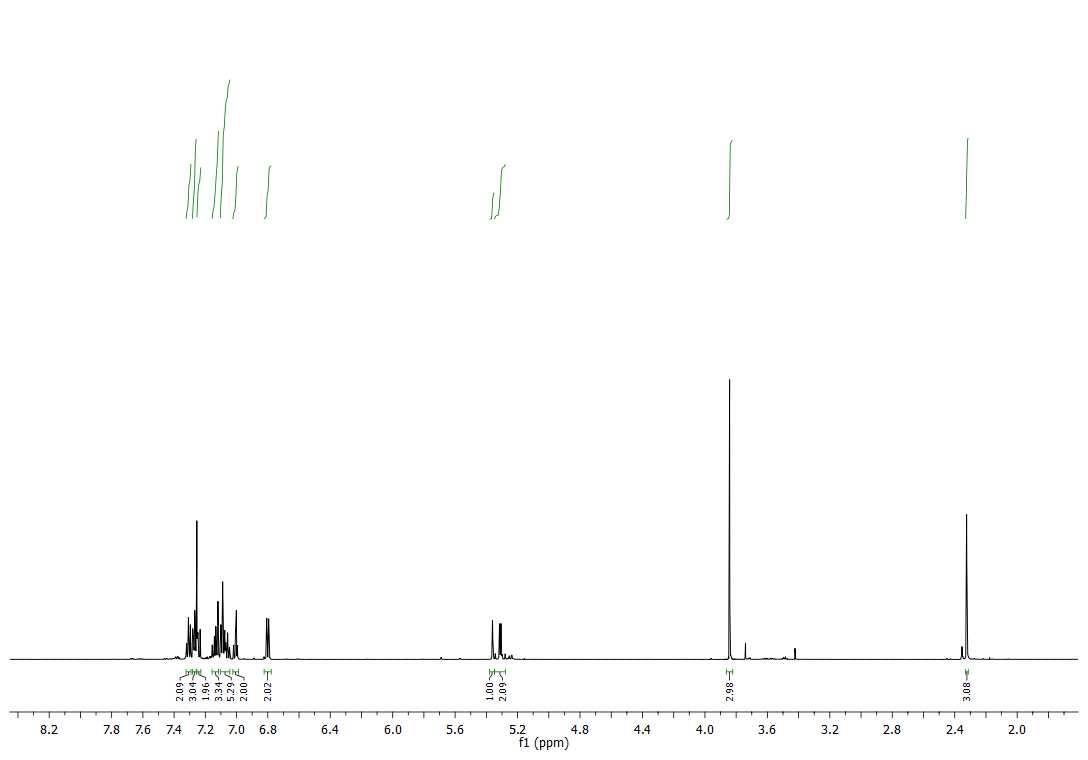
**

**Supplementary Figure 39.** ^1^H NMR spectrum of compound **4n** (600 MHz, CDCl_3_).


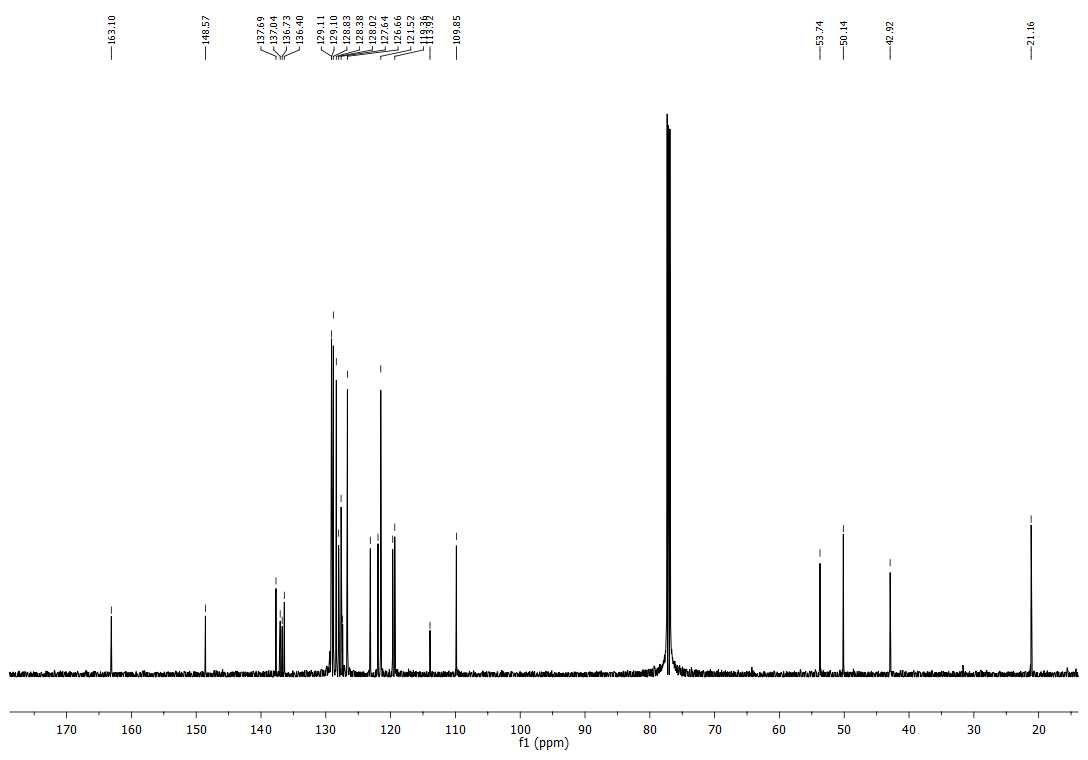


**Supplementary Figure 40.** ^13^C NMR spectrum of compound **4n** (150 MHz, CDCl_3_).

**
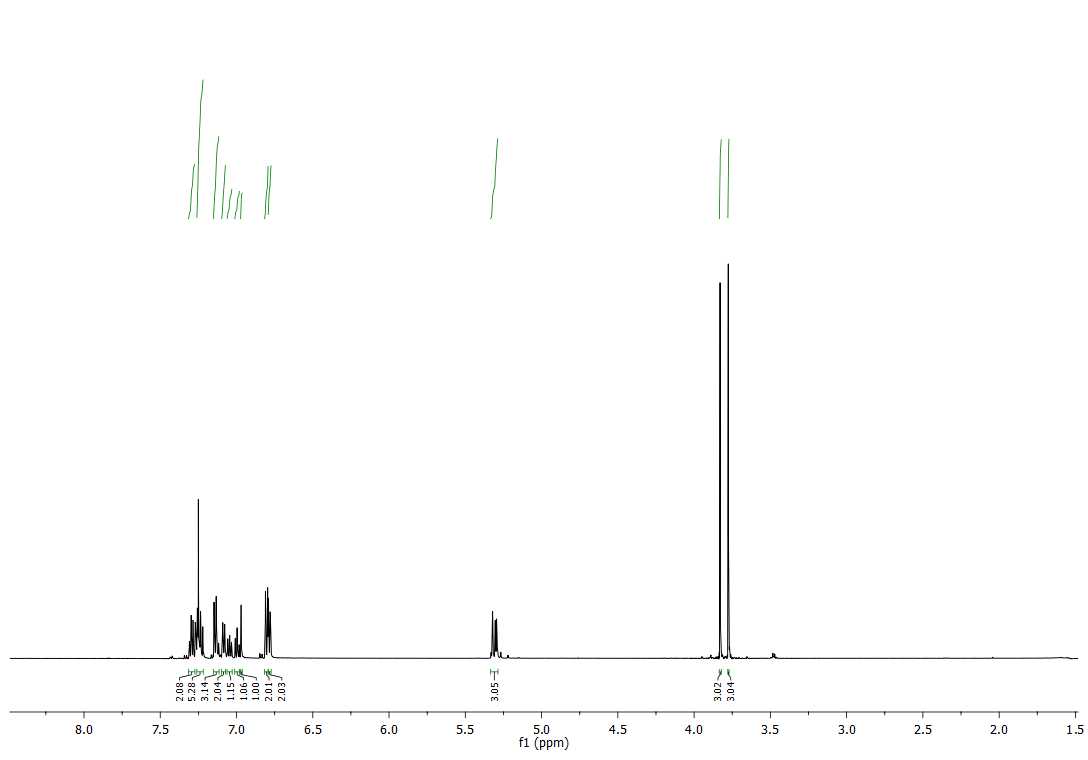
**

**Supplementary Figure 41.** ^1^H NMR spectrum of compound **4o** (600 MHz, CDCl_3_).


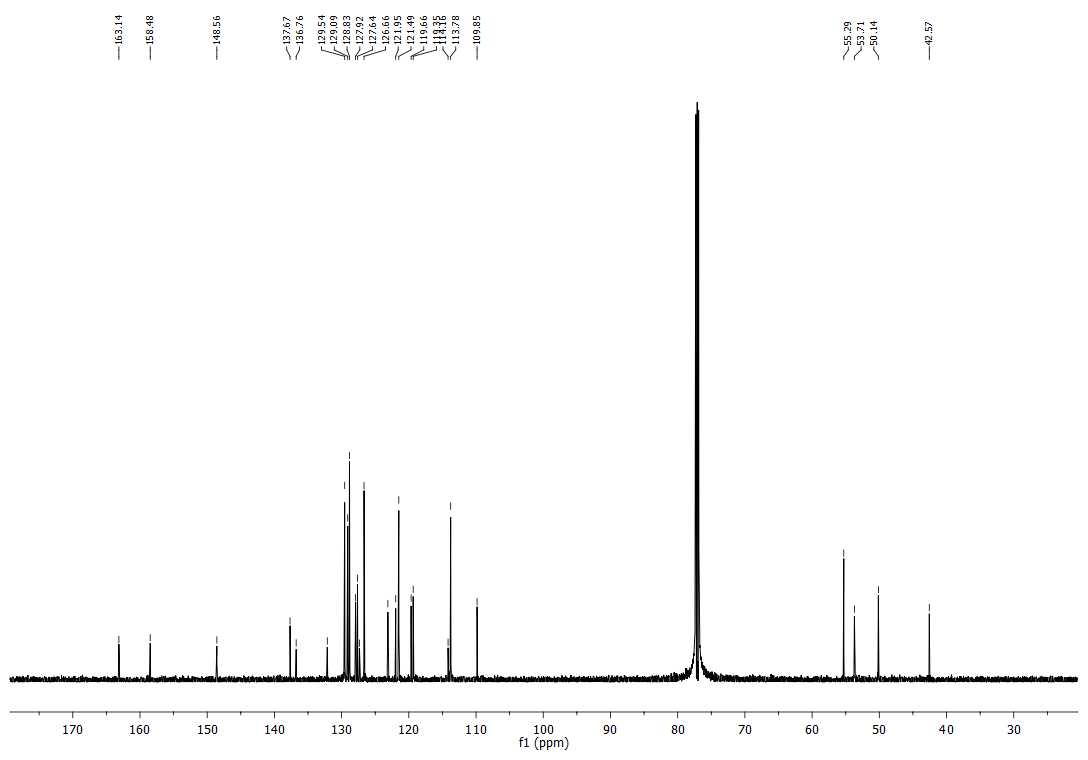


**Supplementary Figure 42.** ^13^C NMR spectrum of compound **4o** (150 MHz, CDCl_3_).

**
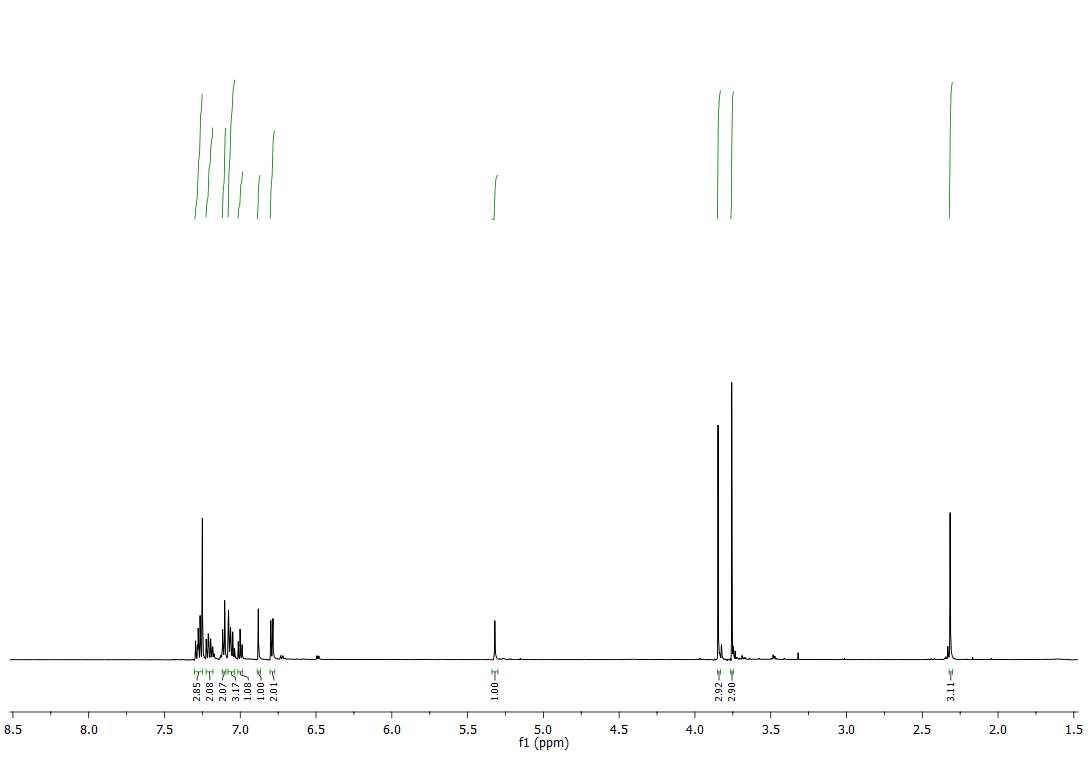
**

**Supplementary Figure 43.** ^1^H NMR spectrum of compound **4p** (600 MHz, CDCl_3_).


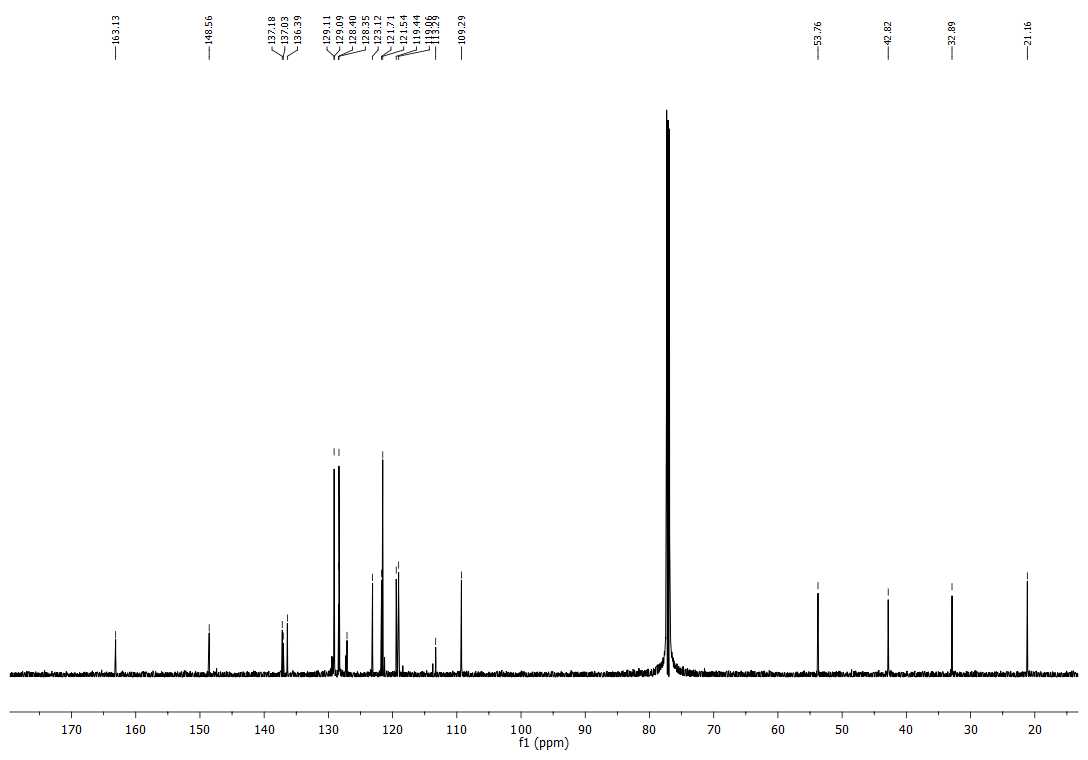


**Supplementary Figure 44.** ^13^C NMR spectrum of compound **4p** (150 MHz, CDCl_3_).

**
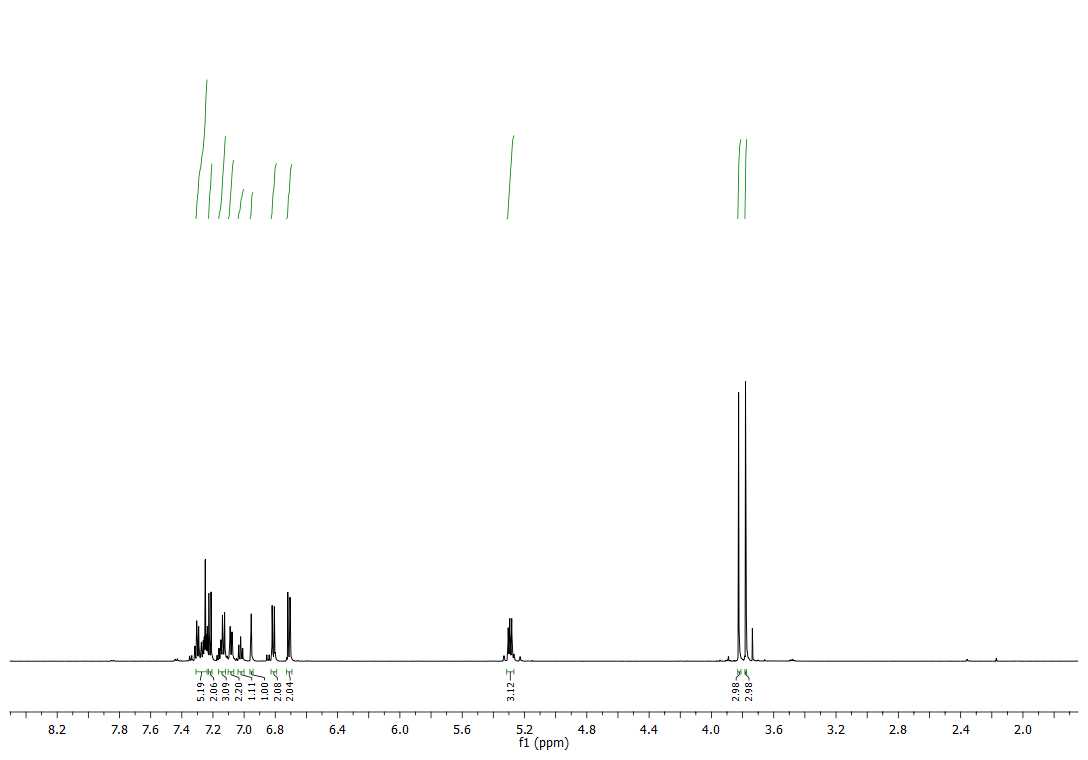
**

**Supplementary Figure 45.** ^1^H NMR spectrum of compound **4q** (600 MHz, CDCl_3_).


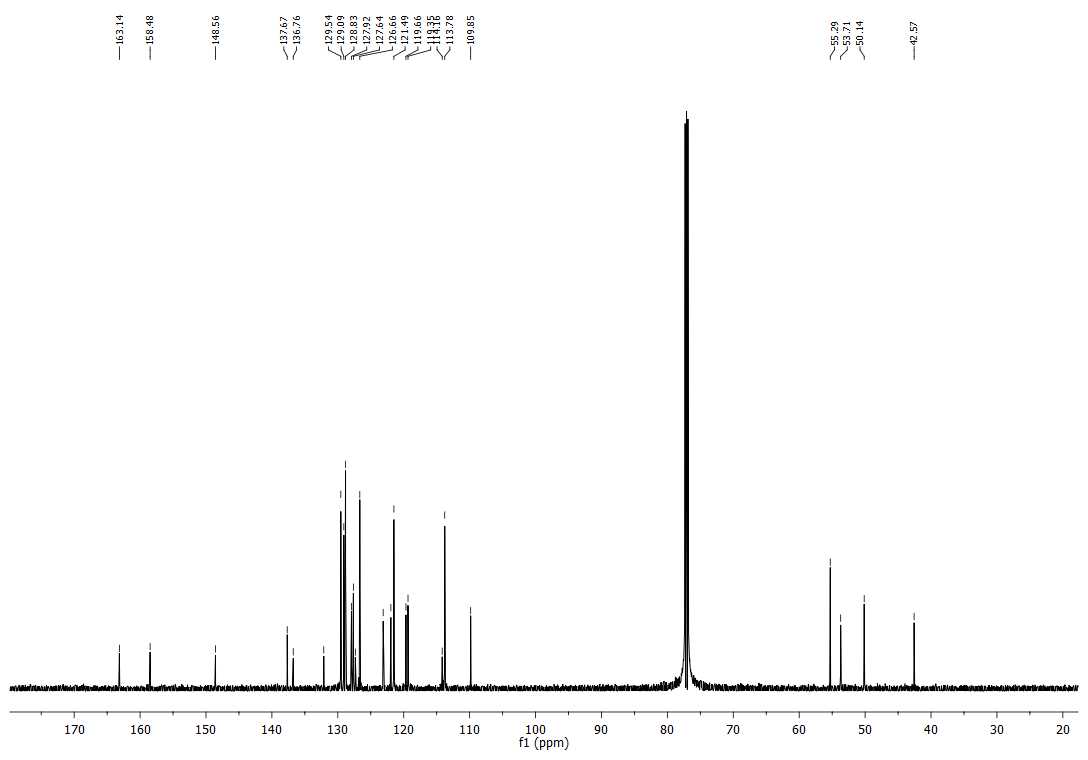


**Supplementary Figure 46.** ^13^C NMR spectrum of compound **4q** (150 MHz, CDCl_3_).

**
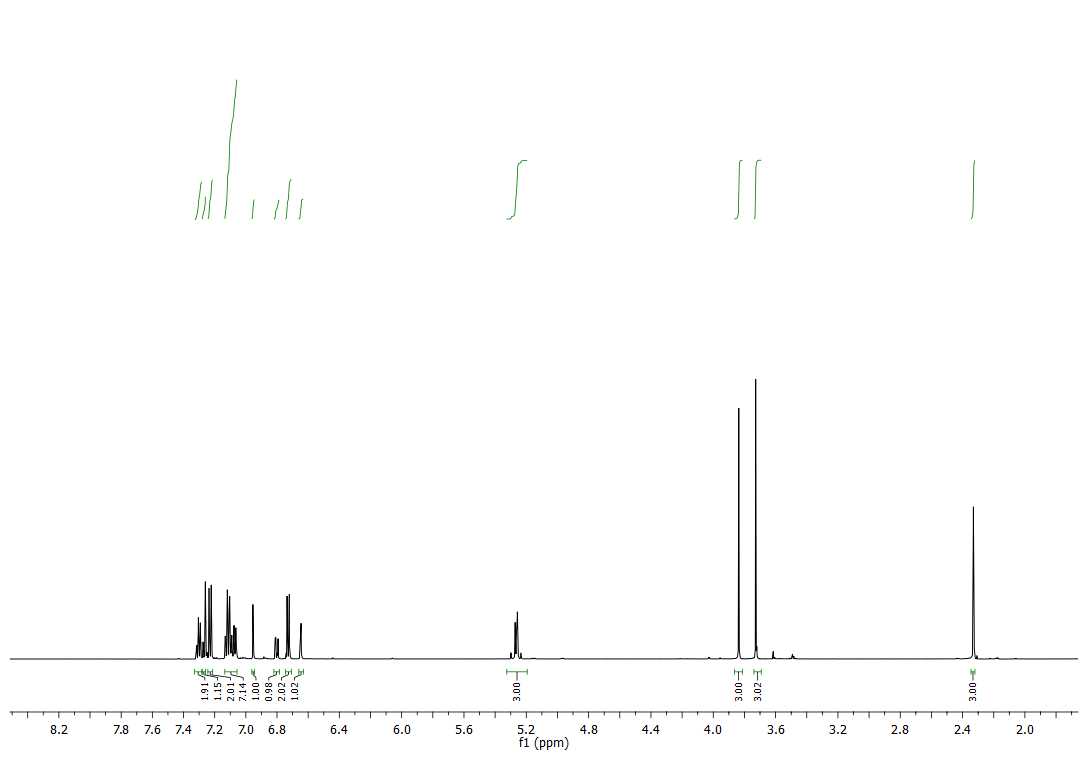
**

**Supplementary Figure 47.** ^1^H NMR spectrum of compound **4r** (600 MHz, CDCl_3_).


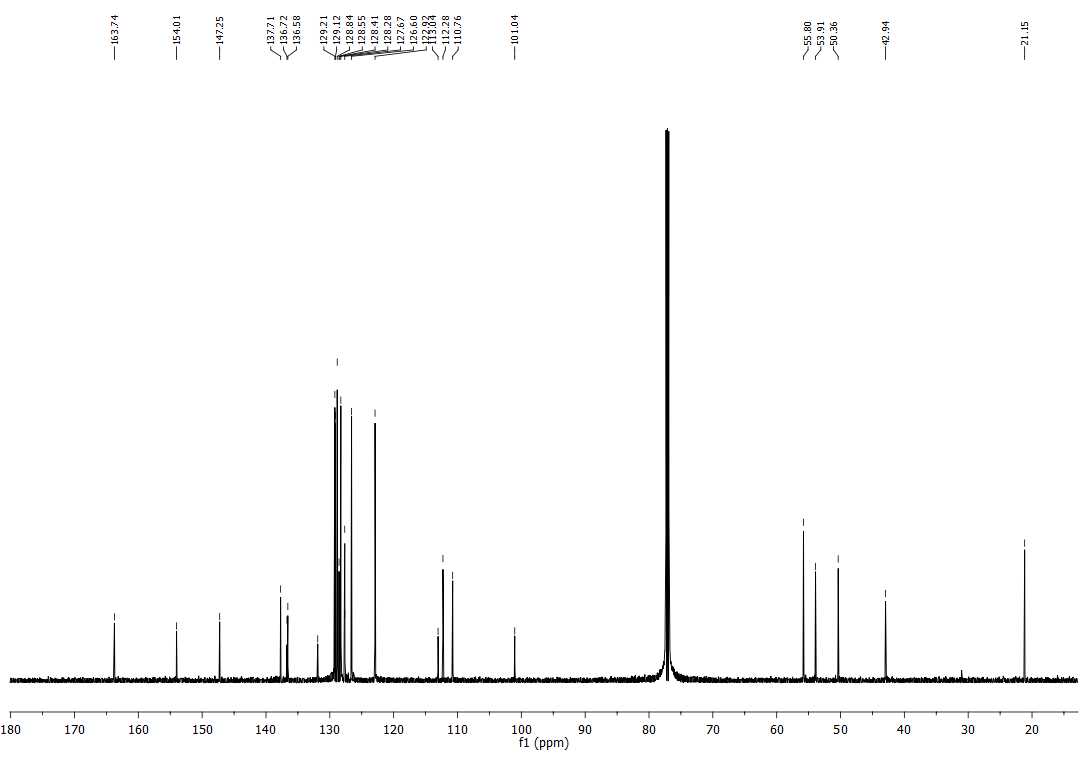


**Supplementary Figure 48.** ^13^C NMR spectrum of compound **4r** (150 MHz, CDCl_3_).

3. ^1^H and ^13^C NMR spectra of ether **5**

**
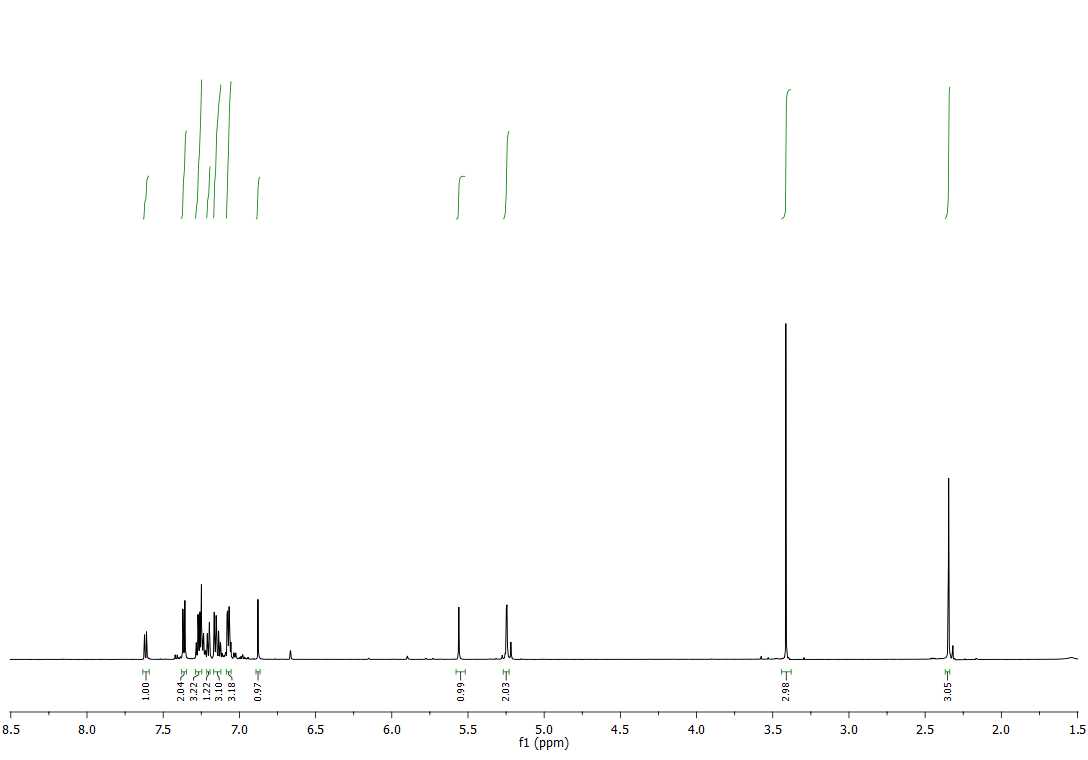
**

**Supplementary Figure 49.** ^1^H NMR spectrum of compound **5** (600 MHz, CDCl_3_).


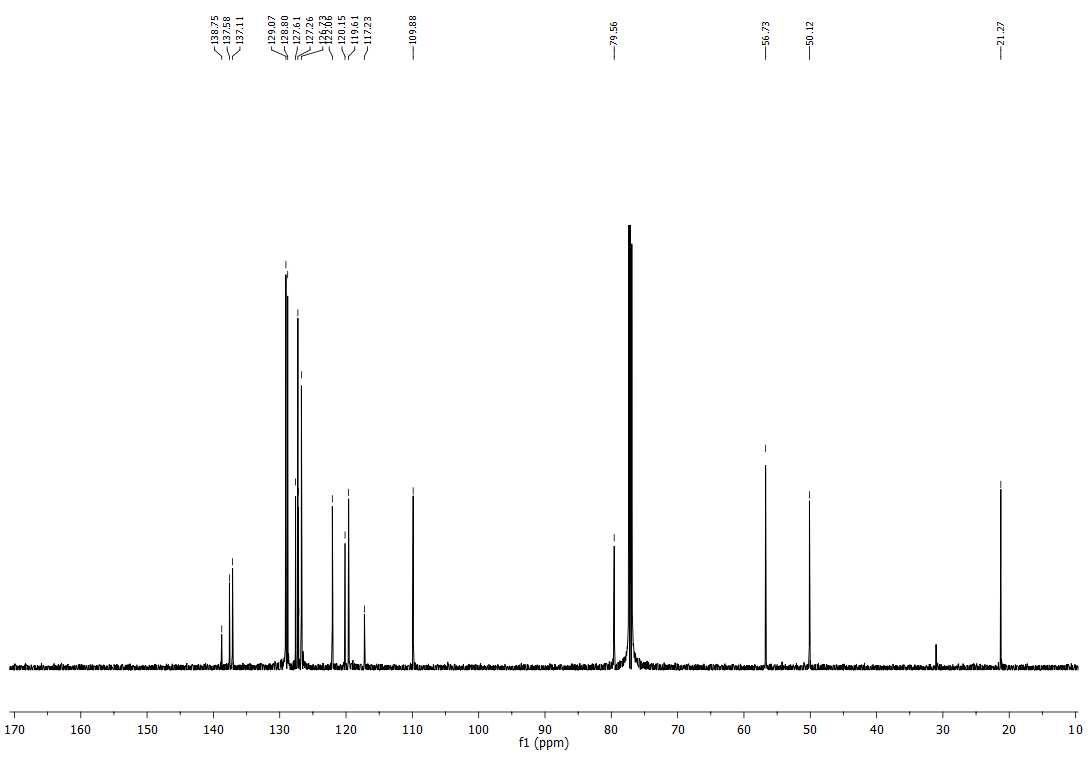


**Supplementary Figure 50.** ^13^C NMR spectrum of compound **5** (150 MHz, CDCl_3_).

4**.** ^1^H and ^13^C NMR spectra of amides **6a-h**

**
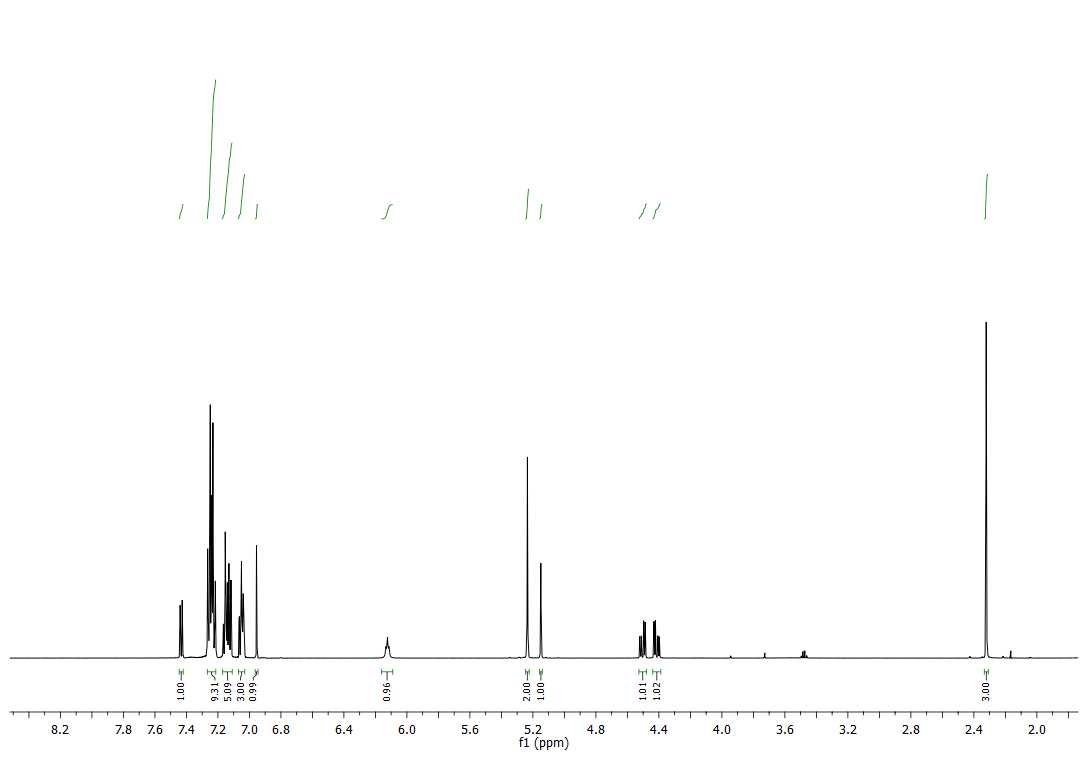
**

**Supplementary Figure 51.** ^1^H NMR spectrum of compound **6a** (600 MHz, CDCl_3_).


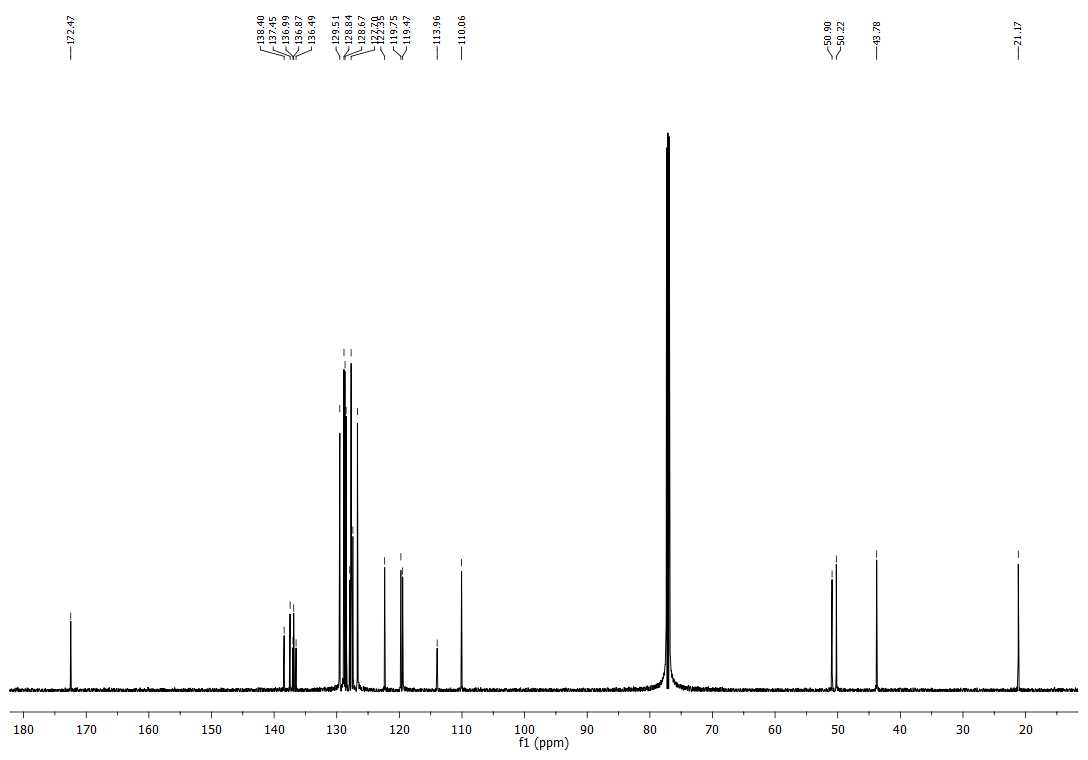


**Supplementary Figure 52.** ^13^C NMR spectrum of compound **6a** (150 MHz, CDCl_3_).

**
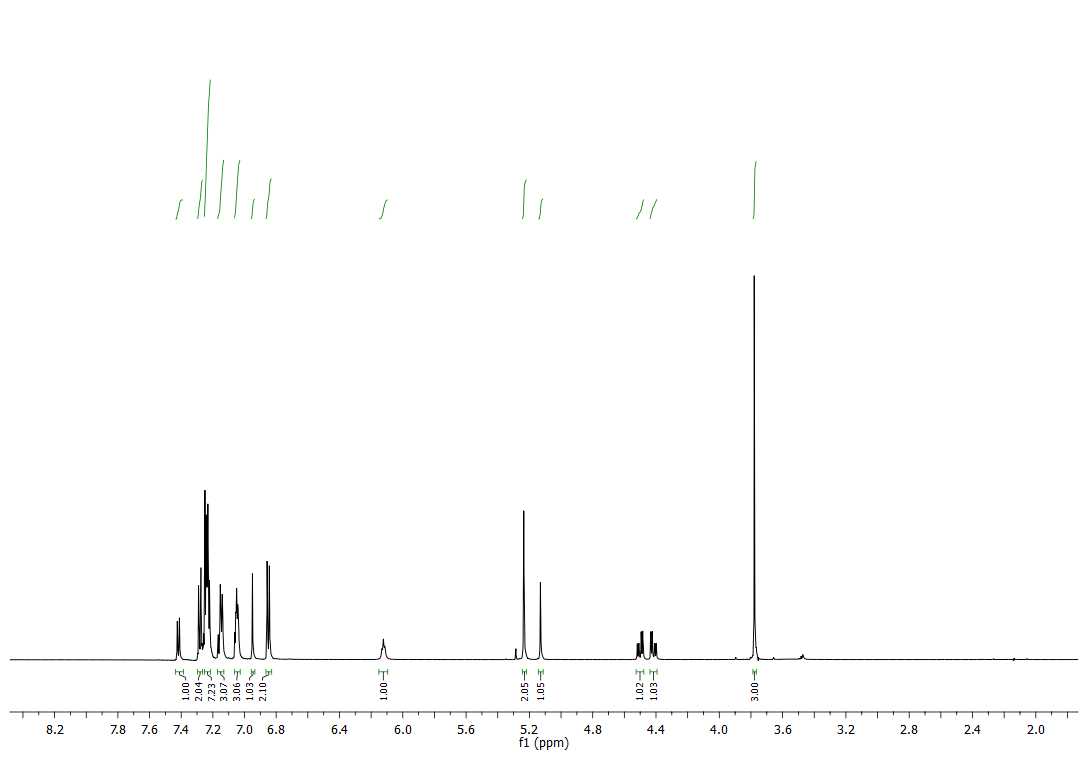
**

**Supplementary Figure 53.** ^1^H NMR spectrum of compound **6b** (600 MHz, CDCl_3_).


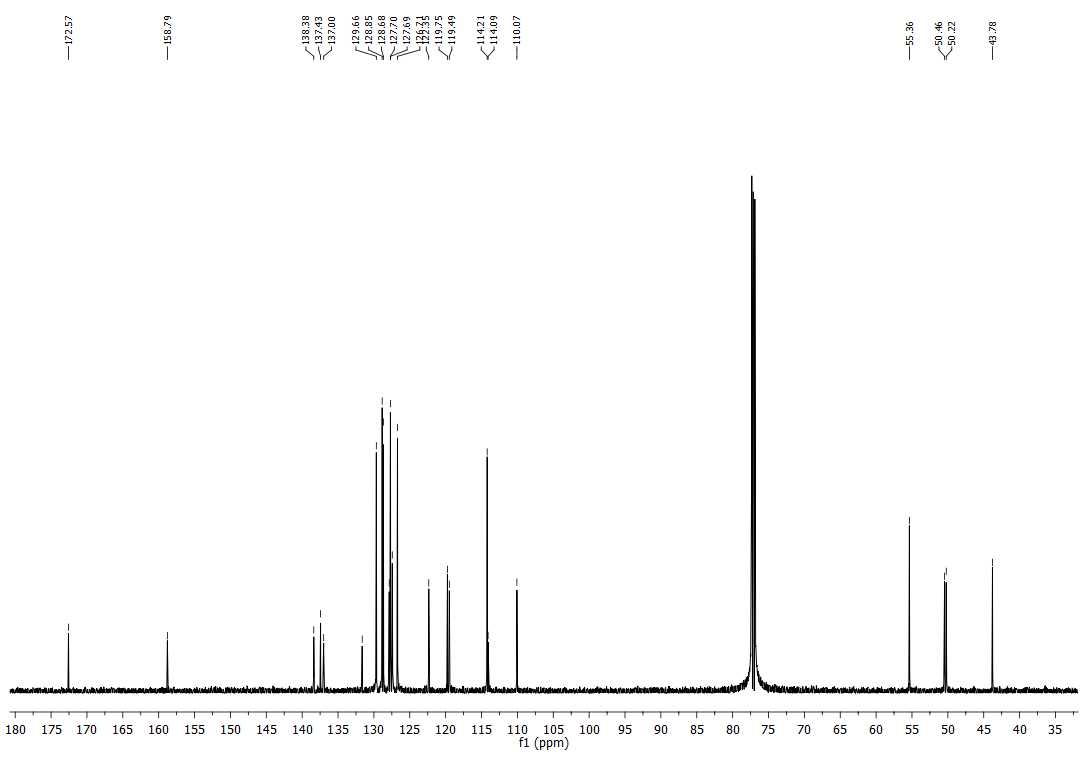


**Supplementary Figure 54.** ^13^C NMR spectrum of compound **6b** (150 MHz, CDCl_3_).

**
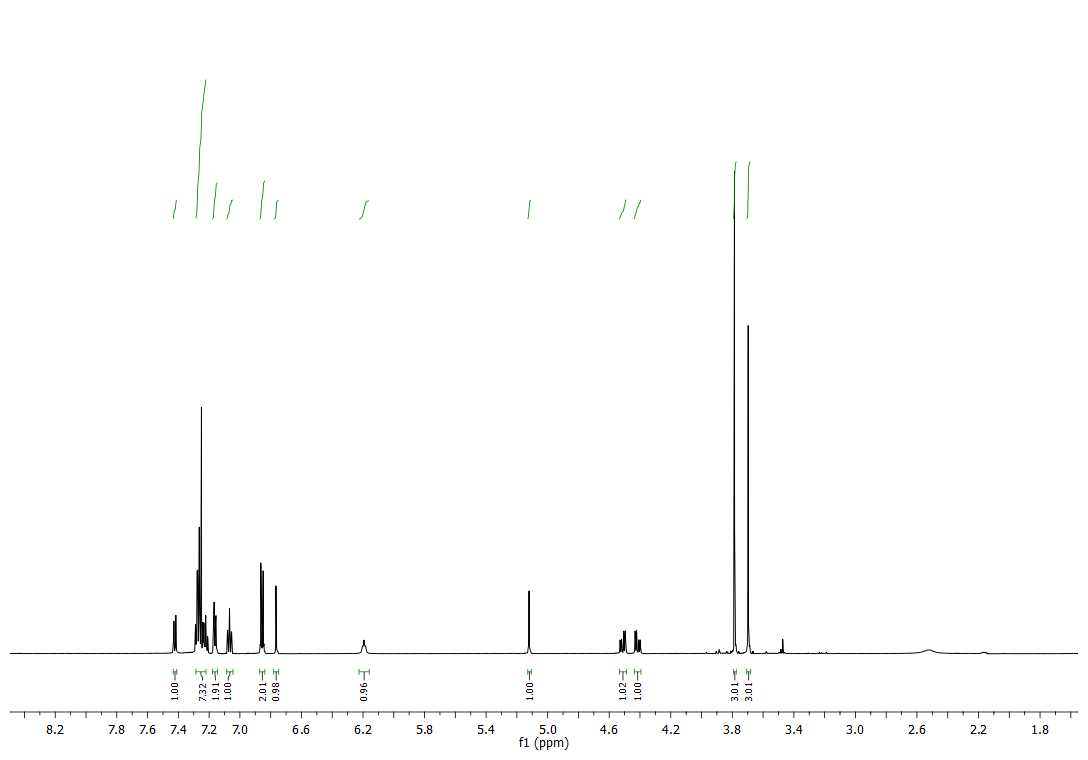
**

**Supplementary Figure 55.** ^1^H NMR spectrum of compound **6c** (600 MHz, CDCl_3_).


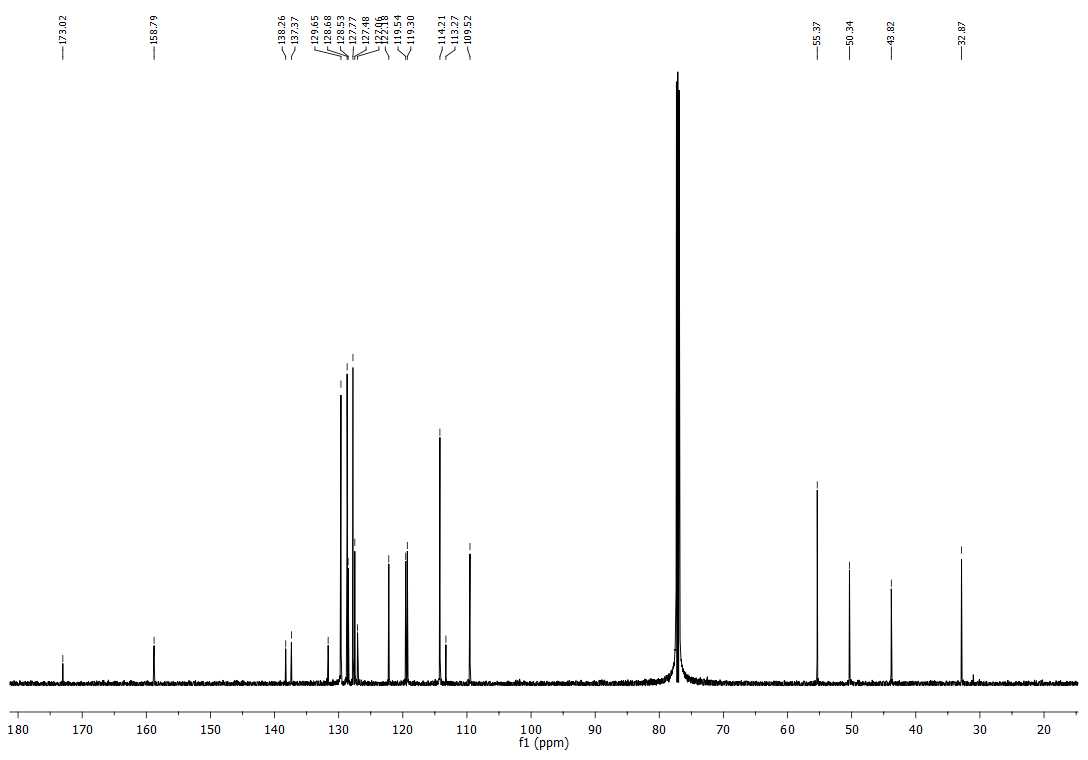


**Supplementary Figure 56.** ^13^C NMR spectrum of compound **6c** (150 MHz, CDCl_3_).

**
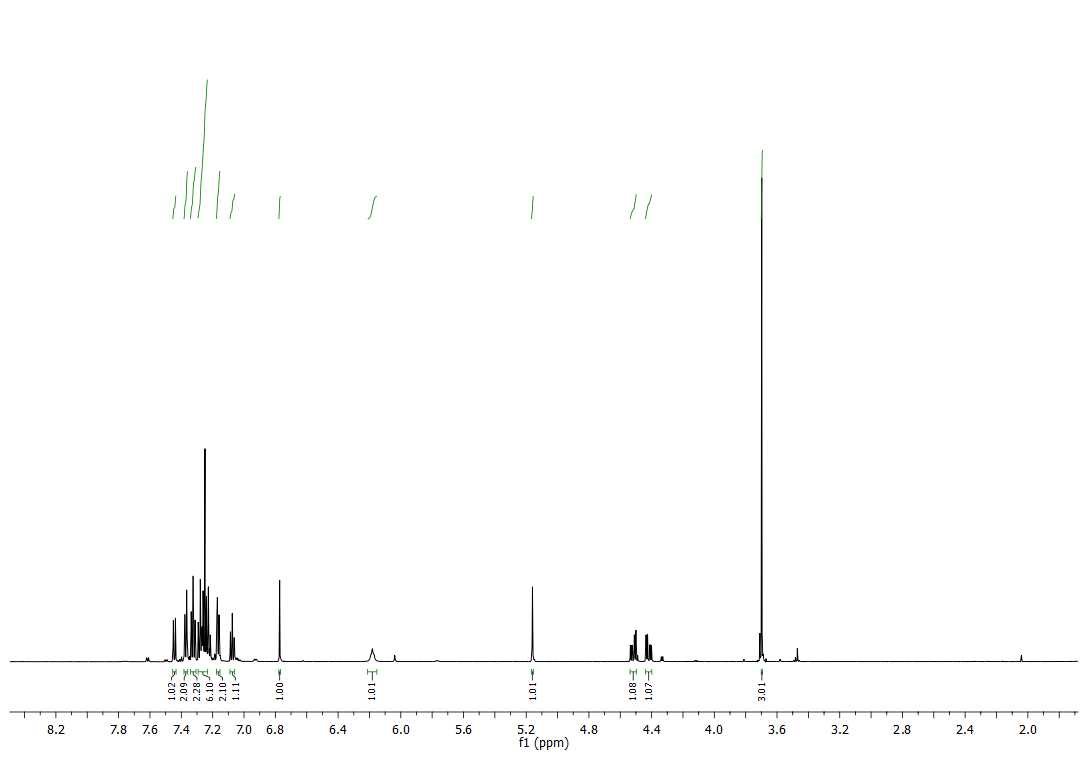
**

**Supplementary Figure 57.** ^1^H NMR spectrum of compound **6d** (600 MHz, CDCl_3_).


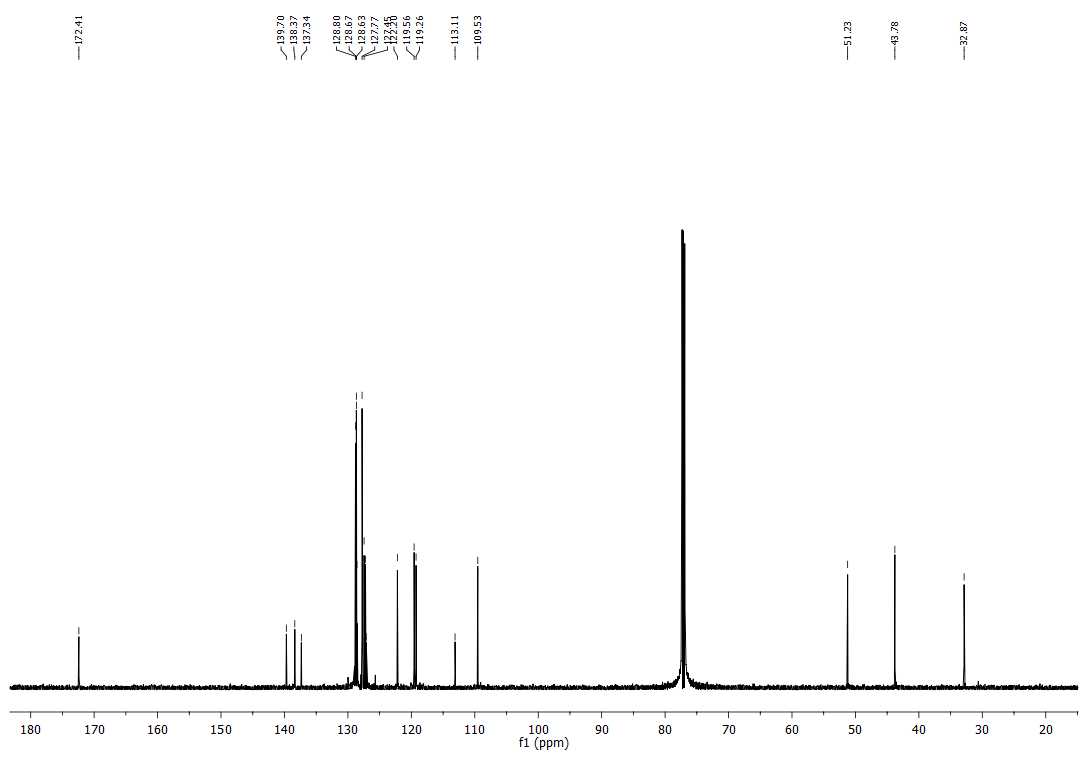


**Supplementary Figure 58.** ^13^C NMR spectrum of compound **6d** (150 MHz, CDCl_3_).

**
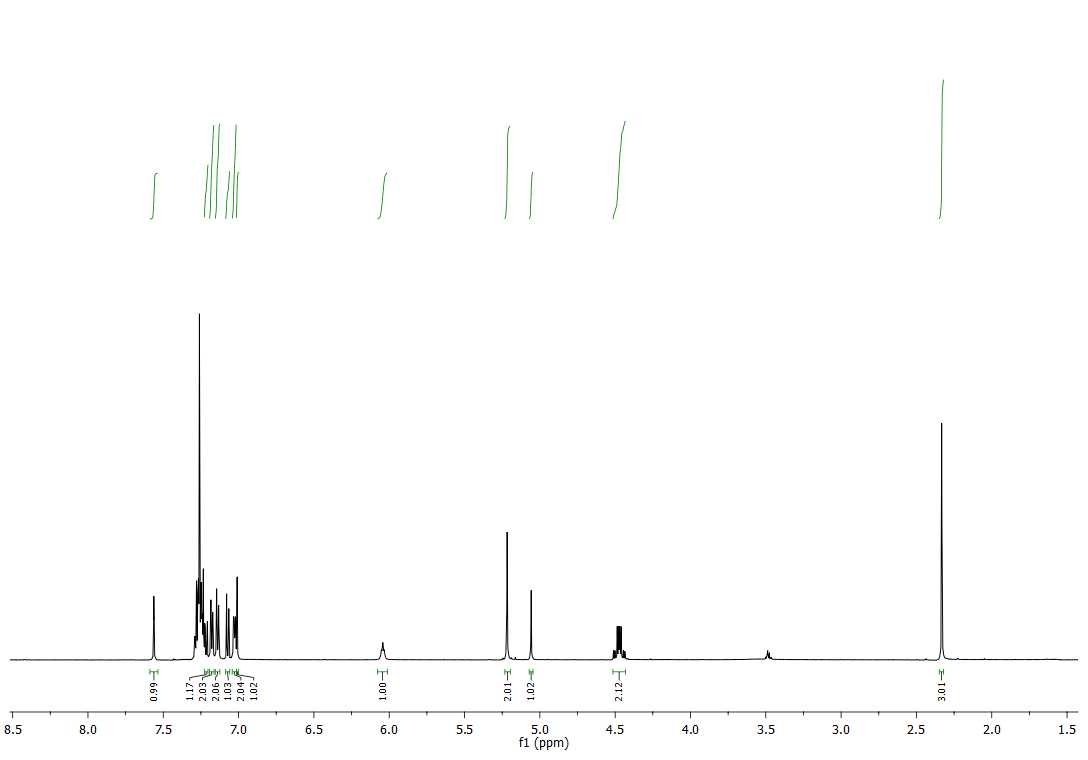
**

**Supplementary Figure 59.** ^1^H NMR spectrum of compound **6e** (600 MHz, CDCl_3_).


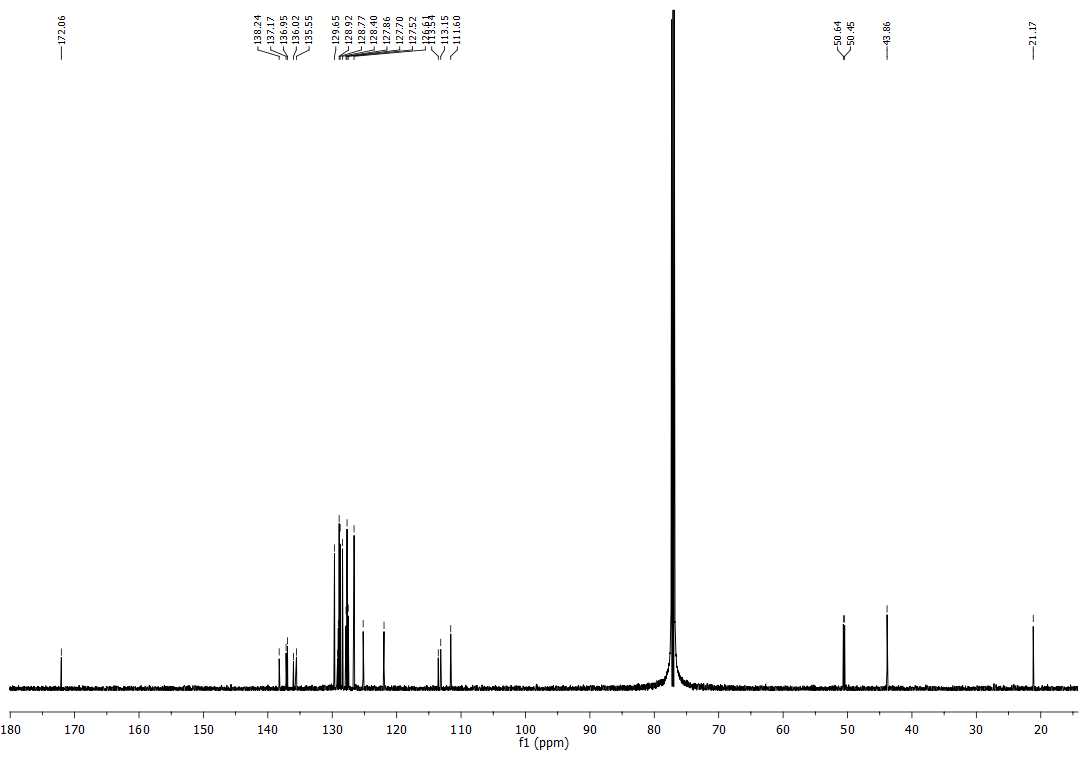


**Supplementary Figure 60.** ^13^C NMR spectrum of compound **6e** (150 MHz, CDCl_3_).

**
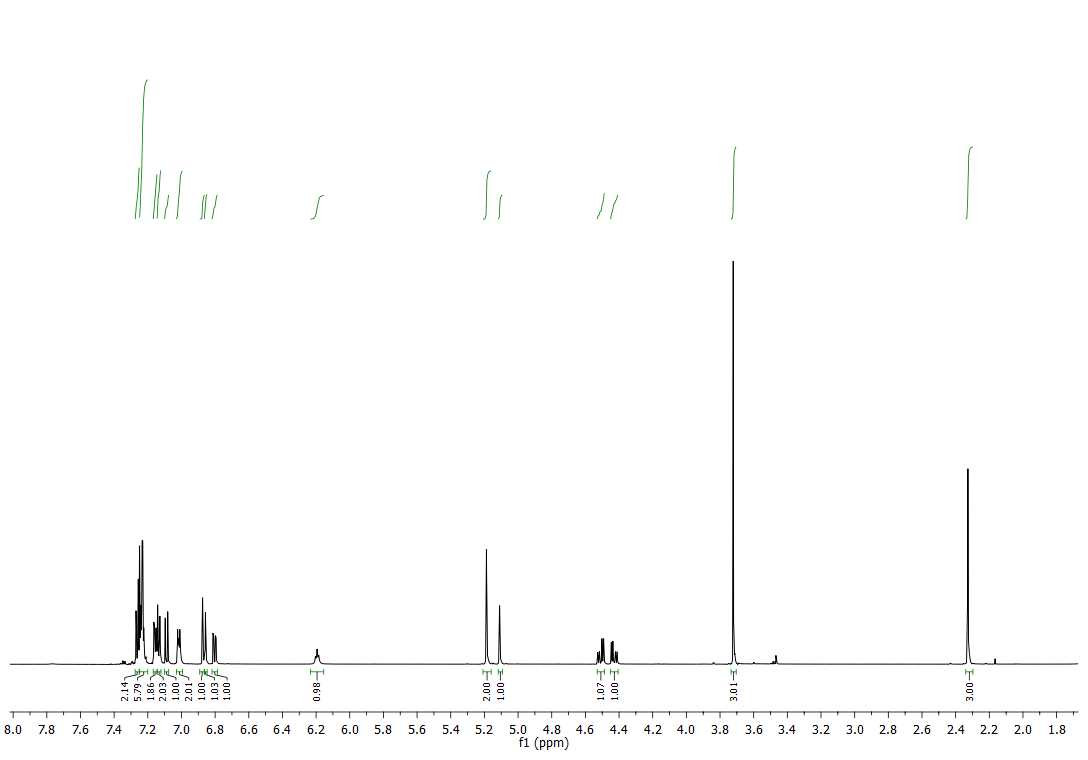
**

**Supplementary Figure 61.** ^1^H NMR spectrum of compound **6f** (600 MHz, CDCl_3_).


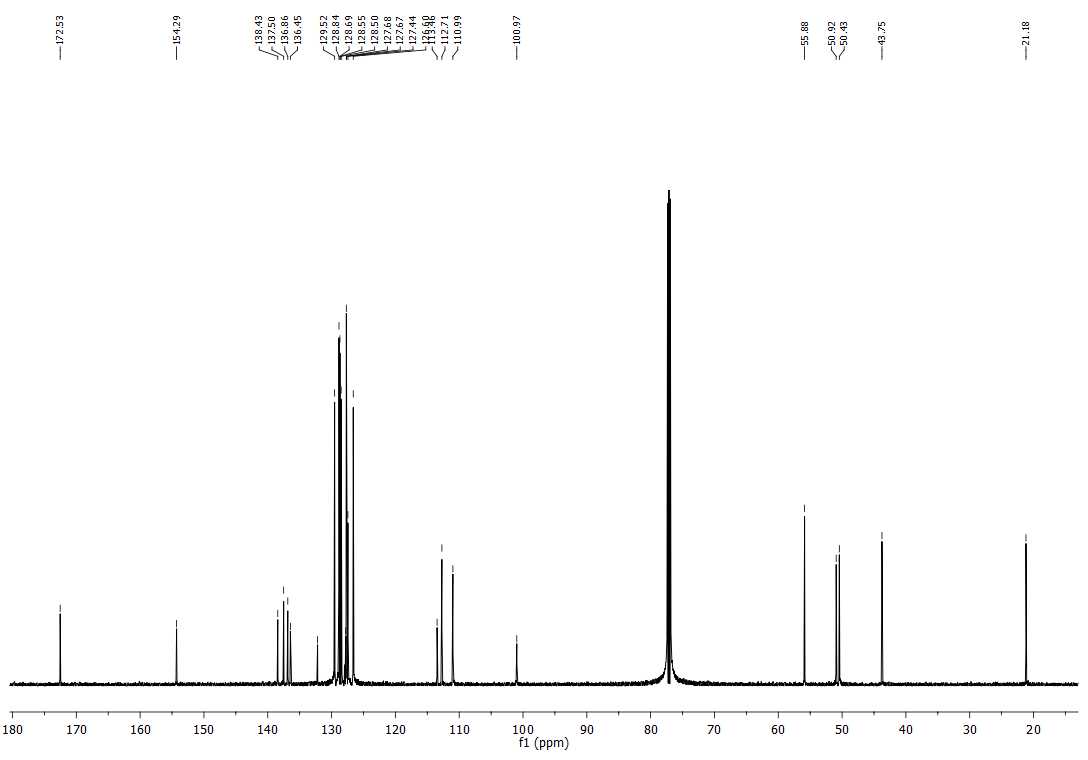


**Supplementary Figure 62.** ^13^C NMR spectrum of compound **6f** (150 MHz, CDCl_3_).

**
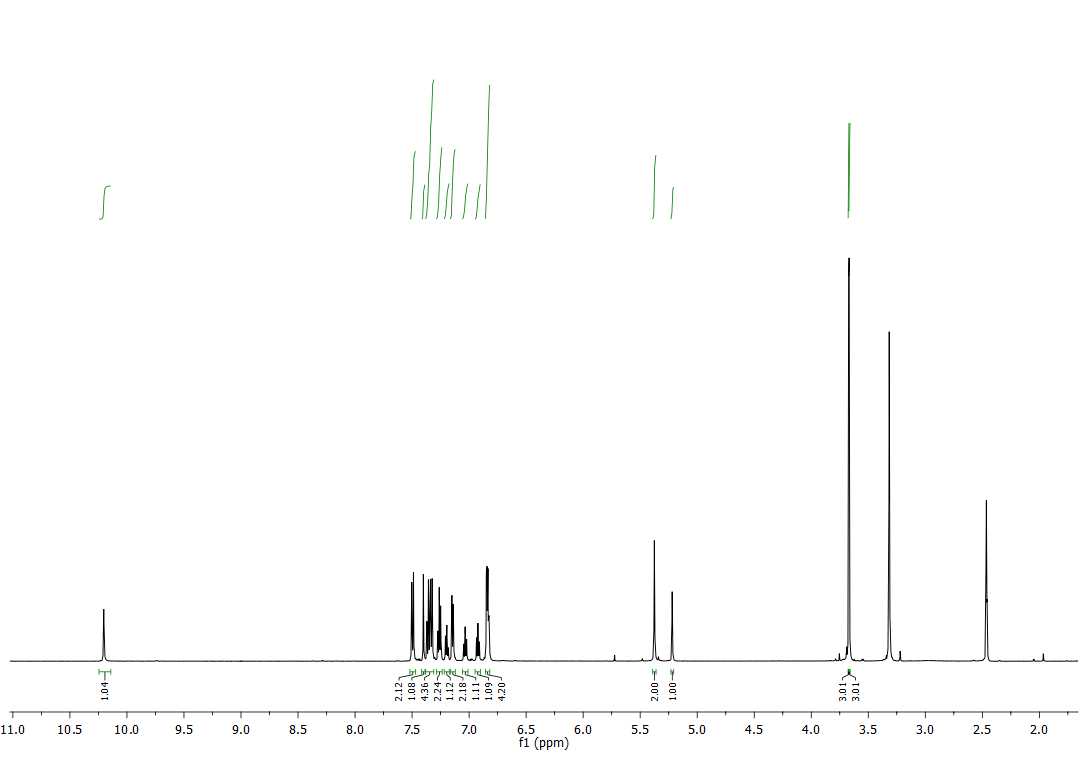
**

**Supplementary Figure 63.** ^1^H NMR spectrum of compound **6g** (600 MHz, DMSO_*d6*).


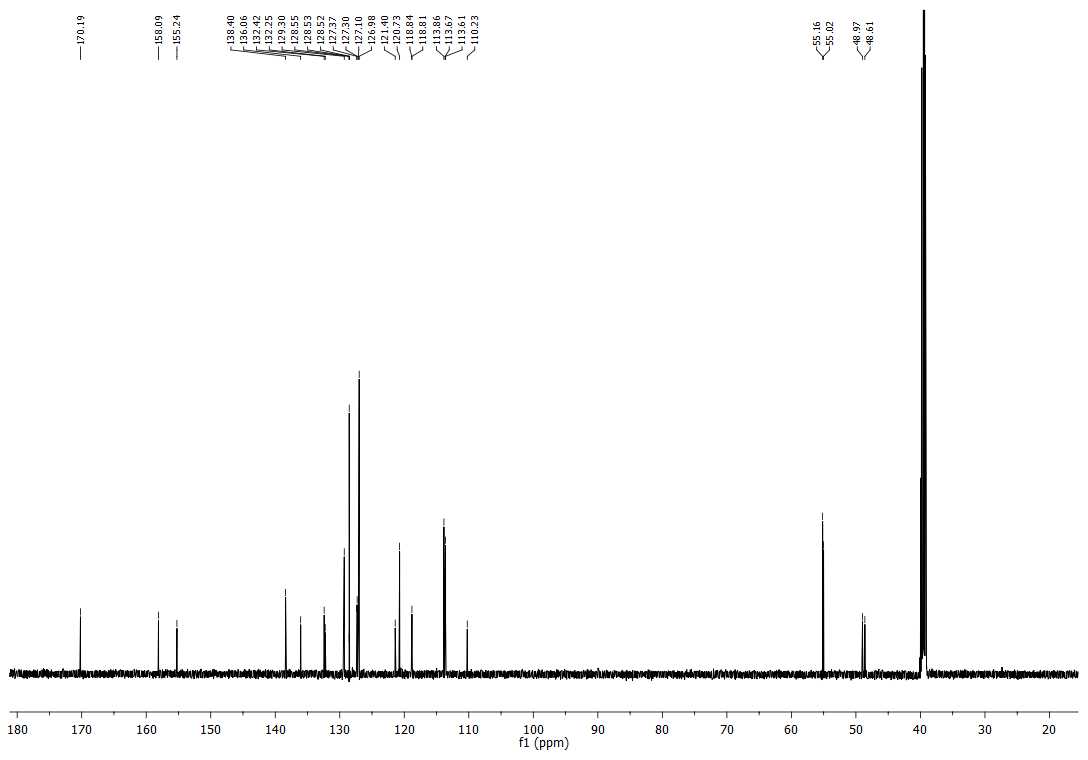


**Supplementary Figure 64.** ^13^C NMR spectrum of compound **6g** (150 MHz, DMSO_*d6*).

**
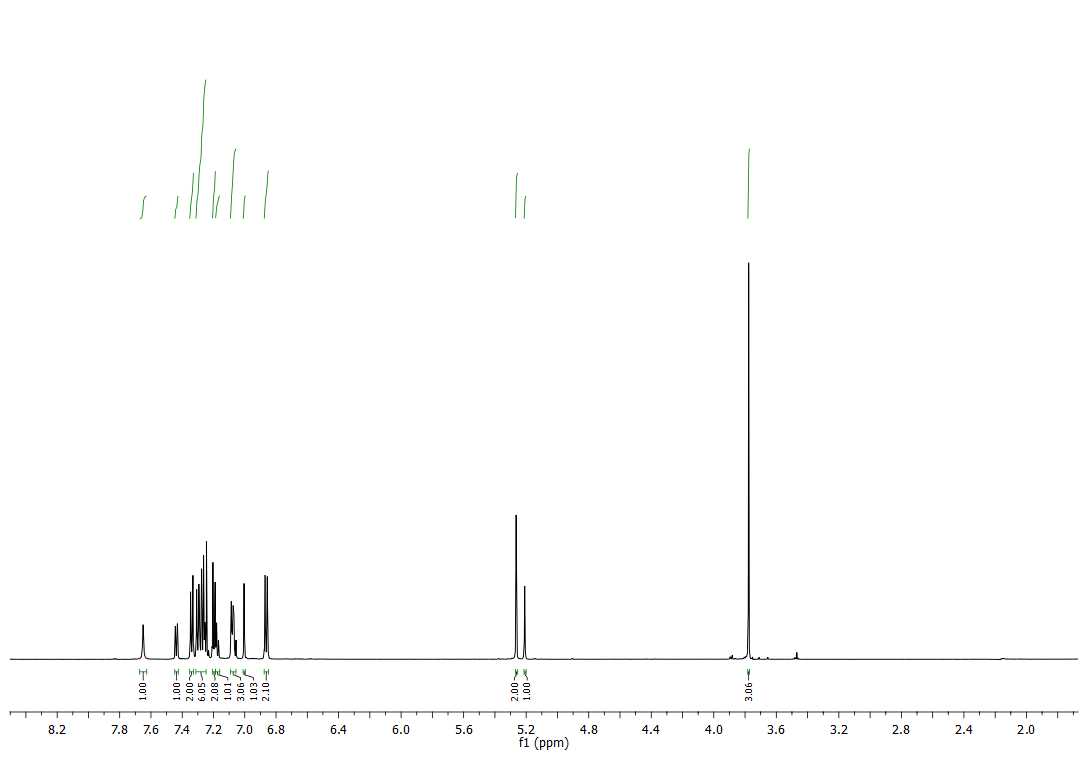
**

**Supplementary Figure 65.** ^1^H NMR spectrum of compound **6h** (600 MHz, CDCl_3_).


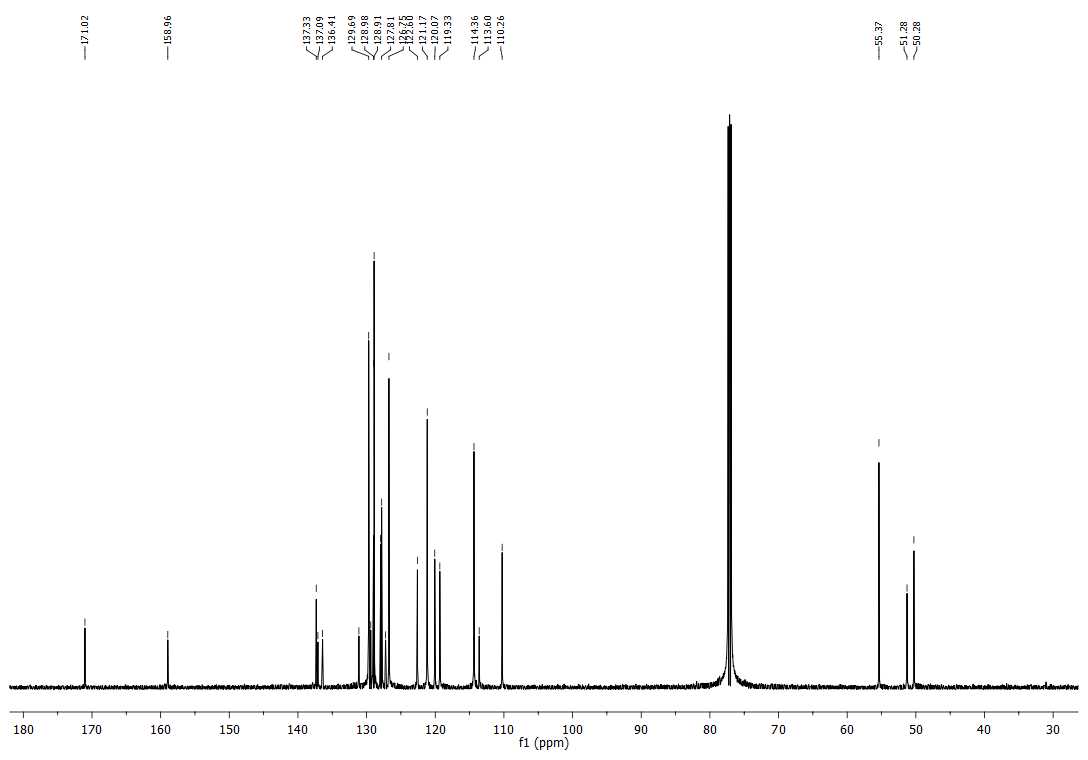


**Supplementary Figure 66.** ^13^C NMR spectrum of compound **6h** (150 MHz, CDCl_3_).
